# Measurements of the Higgs boson production and decay rates and coupling strengths using *pp* collision data at $\sqrt{s}=7$ and 8 TeV in the ATLAS experiment

**DOI:** 10.1140/epjc/s10052-015-3769-y

**Published:** 2016-01-05

**Authors:** G. Aad, B. Abbott, J. Abdallah, O. Abdinov, R. Aben, M. Abolins, O. S. AbouZeid, H. Abramowicz, H. Abreu, R. Abreu, Y. Abulaiti, B. S. Acharya, L. Adamczyk, D. L. Adams, J. Adelman, S. Adomeit, T. Adye, A. A. Affolder, T. Agatonovic-Jovin, J. A. Aguilar-Saavedra, S. P. Ahlen, F. Ahmadov, G. Aielli, H. Akerstedt, T. P. A. Åkesson, G. Akimoto, A. V. Akimov, G. L. Alberghi, J. Albert, S. Albrand, M. J. Alconada Verzini, M. Aleksa, I. N. Aleksandrov, C. Alexa, G. Alexander, T. Alexopoulos, M. Alhroob, G. Alimonti, L. Alio, J. Alison, S. P. Alkire, B. M. M. Allbrooke, P. P. Allport, A. Aloisio, A. Alonso, F. Alonso, C. Alpigiani, A. Altheimer, B. Alvarez Gonzalez, D. Álvarez Piqueras, M. G. Alviggi, B. T. Amadio, K. Amako, Y. Amaral Coutinho, C. Amelung, D. Amidei, S. P. Amor Dos Santos, A. Amorim, S. Amoroso, N. Amram, G. Amundsen, C. Anastopoulos, L. S. Ancu, N. Andari, T. Andeen, C. F. Anders, G. Anders, J. K. Anders, K. J. Anderson, A. Andreazza, V. Andrei, S. Angelidakis, I. Angelozzi, P. Anger, A. Angerami, F. Anghinolfi, A. V. Anisenkov, N. Anjos, A. Annovi, M. Antonelli, A. Antonov, J. Antos, F. Anulli, M. Aoki, L. Aperio Bella, G. Arabidze, Y. Arai, J. P. Araque, A. T. H. Arce, F. A. Arduh, J-F. Arguin, S. Argyropoulos, M. Arik, A. J. Armbruster, O. Arnaez, V. Arnal, H. Arnold, M. Arratia, O. Arslan, A. Artamonov, G. Artoni, S. Asai, N. Asbah, A. Ashkenazi, B. Åsman, L. Asquith, K. Assamagan, R. Astalos, M. Atkinson, N. B. Atlay, B. Auerbach, K. Augsten, M. Aurousseau, G. Avolio, B. Axen, M. K. Ayoub, G. Azuelos, M. A. Baak, A. E. Baas, C. Bacci, H. Bachacou, K. Bachas, M. Backes, M. Backhaus, P. Bagiacchi, P. Bagnaia, Y. Bai, T. Bain, J. T. Baines, O. K. Baker, P. Balek, T. Balestri, F. Balli, E. Banas, Sw. Banerjee, A. A. E. Bannoura, H. S. Bansil, L. Barak, E. L. Barberio, D. Barberis, M. Barbero, T. Barillari, M. Barisonzi, T. Barklow, N. Barlow, S. L. Barnes, B. M. Barnett, R. M. Barnett, Z. Barnovska, A. Baroncelli, G. Barone, A. J. Barr, F. Barreiro, J. Barreiro Guimarães da Costa, R. Bartoldus, A. E. Barton, P. Bartos, A. Basalaev, A. Bassalat, A. Basye, R. L. Bates, S. J. Batista, J. R. Batley, M. Battaglia, M. Bauce, F. Bauer, H. S. Bawa, J. B. Beacham, M. D. Beattie, T. Beau, P. H. Beauchemin, R. Beccherle, P. Bechtle, H. P. Beck, K. Becker, M. Becker, S. Becker, M. Beckingham, C. Becot, A. J. Beddall, A. Beddall, V. A. Bednyakov, C. P. Bee, L. J. Beemster, T. A. Beermann, M. Begel, J. K. Behr, C. Belanger-Champagne, W. H. Bell, G. Bella, L. Bellagamba, A. Bellerive, M. Bellomo, K. Belotskiy, O. Beltramello, O. Benary, D. Benchekroun, M. Bender, K. Bendtz, N. Benekos, Y. Benhammou, E. Benhar Noccioli, J. A. Benitez Garcia, D. P. Benjamin, J. R. Bensinger, S. Bentvelsen, L. Beresford, M. Beretta, D. Berge, E. Bergeaas Kuutmann, N. Berger, F. Berghaus, J. Beringer, C. Bernard, N. R. Bernard, C. Bernius, F. U. Bernlochner, T. Berry, P. Berta, C. Bertella, G. Bertoli, F. Bertolucci, C. Bertsche, D. Bertsche, M. I. Besana, G. J. Besjes, O. Bessidskaia Bylund, M. Bessner, N. Besson, C. Betancourt, S. Bethke, A. J. Bevan, W. Bhimji, R. M. Bianchi, L. Bianchini, M. Bianco, O. Biebel, S. P. Bieniek, M. Biglietti, J. Bilbao De Mendizabal, H. Bilokon, M. Bindi, S. Binet, A. Bingul, C. Bini, C. W. Black, J. E. Black, K. M. Black, D. Blackburn, R. E. Blair, J.-B. Blanchard, J. E. Blanco, T. Blazek, I. Bloch, C. Blocker, W. Blum, U. Blumenschein, G. J. Bobbink, V. S. Bobrovnikov, S. S. Bocchetta, A. Bocci, C. Bock, M. Boehler, J. A. Bogaerts, A. G. Bogdanchikov, C. Bohm, V. Boisvert, T. Bold, V. Boldea, A. S. Boldyrev, M. Bomben, M. Bona, M. Boonekamp, A. Borisov, G. Borissov, S. Borroni, J. Bortfeldt, V. Bortolotto, K. Bos, D. Boscherini, M. Bosman, J. Boudreau, J. Bouffard, E. V. Bouhova-Thacker, D. Boumediene, C. Bourdarios, N. Bousson, A. Boveia, J. Boyd, I. R. Boyko, I. Bozic, J. Bracinik, A. Brandt, G. Brandt, O. Brandt, U. Bratzler, B. Brau, J. E. Brau, H. M. Braun, S. F. Brazzale, K. Brendlinger, A. J. Brennan, L. Brenner, R. Brenner, S. Bressler, K. Bristow, T. M. Bristow, D. Britton, D. Britzger, F. M. Brochu, I. Brock, R. Brock, J. Bronner, G. Brooijmans, T. Brooks, W. K. Brooks, J. Brosamer, E. Brost, J. Brown, P. A. Bruckman de Renstrom, D. Bruncko, R. Bruneliere, A. Bruni, G. Bruni, M. Bruschi, L. Bryngemark, T. Buanes, Q. Buat, P. Buchholz, A. G. Buckley, S. I. Buda, I. A. Budagov, F. Buehrer, L. Bugge, M. K. Bugge, O. Bulekov, D. Bullock, H. Burckhart, S. Burdin, B. Burghgrave, S. Burke, I. Burmeister, E. Busato, D. Büscher, V. Büscher, P. Bussey, J. M. Butler, A. I. Butt, C. M. Buttar, J. M. Butterworth, P. Butti, W. Buttinger, A. Buzatu, A. R. Buzykaev, S. Cabrera Urbán, D. Caforio, V. M. Cairo, O. Cakir, P. Calafiura, A. Calandri, G. Calderini, P. Calfayan, L. P. Caloba, D. Calvet, S. Calvet, R. Camacho Toro, S. Camarda, P. Camarri, D. Cameron, L. M. Caminada, R. Caminal Armadans, S. Campana, M. Campanelli, A. Campoverde, V. Canale, A. Canepa, M. Cano Bret, J. Cantero, R. Cantrill, T. Cao, M. D. M. Capeans Garrido, I. Caprini, M. Caprini, M. Capua, R. Caputo, R. Cardarelli, T. Carli, G. Carlino, L. Carminati, S. Caron, E. Carquin, G. D. Carrillo-Montoya, J. R. Carter, J. Carvalho, D. Casadei, M. P. Casado, M. Casolino, E. Castaneda-Miranda, A. Castelli, V. Castillo Gimenez, N. F. Castro, P. Catastini, A. Catinaccio, J. R. Catmore, A. Cattai, J. Caudron, V. Cavaliere, D. Cavalli, M. Cavalli-Sforza, V. Cavasinni, F. Ceradini, B. C. Cerio, K. Cerny, A. S. Cerqueira, A. Cerri, L. Cerrito, F. Cerutti, M. Cerv, A. Cervelli, S. A. Cetin, A. Chafaq, D. Chakraborty, I. Chalupkova, P. Chang, B. Chapleau, J. D. Chapman, D. G. Charlton, C. C. Chau, C. A. Chavez Barajas, S. Cheatham, A. Chegwidden, S. Chekanov, S. V. Chekulaev, G. A. Chelkov, M. A. Chelstowska, C. Chen, H. Chen, K. Chen, L. Chen, S. Chen, X. Chen, Y. Chen, H. C. Cheng, Y. Cheng, A. Cheplakov, E. Cheremushkina, R. Cherkaoui El Moursli, V. Chernyatin, E. Cheu, L. Chevalier, V. Chiarella, J. T. Childers, G. Chiodini, A. S. Chisholm, R. T. Chislett, A. Chitan, M. V. Chizhov, K. Choi, S. Chouridou, B. K. B. Chow, V. Christodoulou, D. Chromek-Burckhart, M. L. Chu, J. Chudoba, A. J. Chuinard, J. J. Chwastowski, L. Chytka, G. Ciapetti, A. K. Ciftci, D. Cinca, V. Cindro, I. A. Cioara, A. Ciocio, Z. H. Citron, M. Ciubancan, A. Clark, B. L. Clark, P. J. Clark, R. N. Clarke, W. Cleland, C. Clement, Y. Coadou, M. Cobal, A. Coccaro, J. Cochran, L. Coffey, J. G. Cogan, B. Cole, S. Cole, A. P. Colijn, J. Collot, T. Colombo, G. Compostella, P. Conde Muiño, E. Coniavitis, S. H. Connell, I. A. Connelly, S. M. Consonni, V. Consorti, S. Constantinescu, C. Conta, G. Conti, F. Conventi, M. Cooke, B. D. Cooper, A. M. Cooper-Sarkar, T. Cornelissen, M. Corradi, F. Corriveau, A. Corso-Radu, A. Cortes-Gonzalez, G. Cortiana, G. Costa, M. J. Costa, D. Costanzo, D. Côté, G. Cottin, G. Cowan, B. E. Cox, K. Cranmer, G. Cree, S. Crépé-Renaudin, F. Crescioli, W. A. Cribbs, M. Crispin Ortuzar, M. Cristinziani, V. Croft, G. Crosetti, T. Cuhadar Donszelmann, J. Cummings, M. Curatolo, C. Cuthbert, H. Czirr, P. Czodrowski, S. D’Auria, M. D’Onofrio, M. J. Da Cunha Sargedas De Sousa, C. Da Via, W. Dabrowski, A. Dafinca, T. Dai, O. Dale, F. Dallaire, C. Dallapiccola, M. Dam, J. R. Dandoy, N. P. Dang, A. C. Daniells, M. Danninger, M. Dano Hoffmann, V. Dao, G. Darbo, S. Darmora, J. Dassoulas, A. Dattagupta, W. Davey, C. David, T. Davidek, E. Davies, M. Davies, P. Davison, Y. Davygora, E. Dawe, I. Dawson, R. K. Daya-Ishmukhametova, K. De, R. de Asmundis, S. De Castro, S. De Cecco, N. De Groot, P. de Jong, H. De la Torre, F. De Lorenzi, L. De Nooij, D. De Pedis, A. De Salvo, U. De Sanctis, A. De Santo, J. B. De Vivie De Regie, W. J. Dearnaley, R. Debbe, C. Debenedetti, D. V. Dedovich, I. Deigaard, J. Del Peso, T. Del Prete, D. Delgove, F. Deliot, C. M. Delitzsch, M. Deliyergiyev, A. Dell’Acqua, L. Dell’Asta, M. Dell’Orso, M. Della Pietra, D. della Volpe, M. Delmastro, P. A. Delsart, C. Deluca, D. A. DeMarco, S. Demers, M. Demichev, A. Demilly, S. P. Denisov, D. Derendarz, J. E. Derkaoui, F. Derue, P. Dervan, K. Desch, C. Deterre, P. O. Deviveiros, A. Dewhurst, S. Dhaliwal, A. Di Ciaccio, L. Di Ciaccio, A. Di Domenico, C. Di Donato, A. Di Girolamo, B. Di Girolamo, A. Di Mattia, B. Di Micco, R. Di Nardo, A. Di Simone, R. Di Sipio, D. Di Valentino, C. Diaconu, M. Diamond, F. A. Dias, M. A. Diaz, E. B. Diehl, J. Dietrich, S. Diglio, A. Dimitrievska, J. Dingfelder, P. Dita, S. Dita, F. Dittus, F. Djama, T. Djobava, J. I. Djuvsland, M. A. B. do Vale, D. Dobos, M. Dobre, C. Doglioni, T. Dohmae, J. Dolejsi, Z. Dolezal, B. A. Dolgoshein, M. Donadelli, S. Donati, P. Dondero, J. Donini, J. Dopke, A. Doria, M. T. Dova, A. T. Doyle, E. Drechsler, M. Dris, E. Dubreuil, E. Duchovni, G. Duckeck, O. A. Ducu, D. Duda, A. Dudarev, L. Duflot, L. Duguid, M. Dührssen, M. Dunford, H. Duran Yildiz, M. Düren, A. Durglishvili, D. Duschinger, M. Dyndal, C. Eckardt, K. M. Ecker, R. C. Edgar, W. Edson, N. C. Edwards, W. Ehrenfeld, T. Eifert, G. Eigen, K. Einsweiler, T. Ekelof, M. El Kacimi, M. Ellert, S. Elles, F. Ellinghaus, A. A. Elliot, N. Ellis, J. Elmsheuser, M. Elsing, D. Emeliyanov, Y. Enari, O. C. Endner, M. Endo, J. Erdmann, A. Ereditato, G. Ernis, J. Ernst, M. Ernst, S. Errede, E. Ertel, M. Escalier, H. Esch, C. Escobar, B. Esposito, A. I. Etienvre, E. Etzion, H. Evans, A. Ezhilov, L. Fabbri, G. Facini, R. M. Fakhrutdinov, S. Falciano, R. J. Falla, J. Faltova, Y. Fang, M. Fanti, A. Farbin, A. Farilla, T. Farooque, S. Farrell, S. M. Farrington, P. Farthouat, F. Fassi, P. Fassnacht, D. Fassouliotis, M. Faucci Giannelli, A. Favareto, L. Fayard, P. Federic, O. L. Fedin, W. Fedorko, S. Feigl, L. Feligioni, C. Feng, E. J. Feng, H. Feng, A. B. Fenyuk, P. Fernandez Martinez, S. Fernandez Perez, J. Ferrando, A. Ferrari, P. Ferrari, R. Ferrari, D. E. Ferreira de Lima, A. Ferrer, D. Ferrere, C. Ferretti, A. Ferretto Parodi, M. Fiascaris, F. Fiedler, A. Filipčič, M. Filipuzzi, F. Filthaut, M. Fincke-Keeler, K. D. Finelli, M. C. N. Fiolhais, L. Fiorini, A. Firan, A. Fischer, C. Fischer, J. Fischer, W. C. Fisher, E. A. Fitzgerald, M. Flechl, I. Fleck, P. Fleischmann, S. Fleischmann, G. T. Fletcher, G. Fletcher, T. Flick, A. Floderus, L. R. Flores Castillo, M. J. Flowerdew, A. Formica, A. Forti, D. Fournier, H. Fox, S. Fracchia, P. Francavilla, M. Franchini, D. Francis, L. Franconi, M. Franklin, M. Fraternali, D. Freeborn, S. T. French, F. Friedrich, D. Froidevaux, J. A. Frost, C. Fukunaga, E. Fullana Torregrosa, B. G. Fulsom, J. Fuster, C. Gabaldon, O. Gabizon, A. Gabrielli, A. Gabrielli, S. Gadatsch, S. Gadomski, G. Gagliardi, P. Gagnon, C. Galea, B. Galhardo, E. J. Gallas, B. J. Gallop, P. Gallus, G. Galster, K. K. Gan, J. Gao, Y. Gao, Y. S. Gao, F. M. Garay Walls, F. Garberson, C. García, J. E. García Navarro, M. Garcia-Sciveres, R. W. Gardner, N. Garelli, V. Garonne, C. Gatti, A. Gaudiello, G. Gaudio, B. Gaur, L. Gauthier, P. Gauzzi, I. L. Gavrilenko, C. Gay, G. Gaycken, E. N. Gazis, P. Ge, Z. Gecse, C. N. P. Gee, D. A. A. Geerts, Ch. Geich-Gimbel, M. P. Geisler, C. Gemme, M. H. Genest, S. Gentile, M. George, S. George, D. Gerbaudo, A. Gershon, H. Ghazlane, B. Giacobbe, S. Giagu, V. Giangiobbe, P. Giannetti, B. Gibbard, S. M. Gibson, M. Gilchriese, T. P. S. Gillam, D. Gillberg, G. Gilles, D. M. Gingrich, N. Giokaris, M. P. Giordani, F. M. Giorgi, F. M. Giorgi, P. F. Giraud, P. Giromini, D. Giugni, C. Giuliani, M. Giulini, B. K. Gjelsten, S. Gkaitatzis, I. Gkialas, E. L. Gkougkousis, L. K. Gladilin, C. Glasman, J. Glatzer, P. C. F. Glaysher, A. Glazov, M. Goblirsch-Kolb, J. R. Goddard, J. Godlewski, S. Goldfarb, T. Golling, D. Golubkov, A. Gomes, R. Gonçalo, J. Goncalves Pinto Firmino Da Costa, L. Gonella, S. González de la Hoz, G. Gonzalez Parra, S. Gonzalez-Sevilla, L. Goossens, P. A. Gorbounov, H. A. Gordon, I. Gorelov, B. Gorini, E. Gorini, A. Gorišek, E. Gornicki, A. T. Goshaw, C. Gössling, M. I. Gostkin, D. Goujdami, A. G. Goussiou, N. Govender, H. M. X. Grabas, L. Graber, I. Grabowska-Bold, P. Grafström, K-J. Grahn, J. Gramling, E. Gramstad, S. Grancagnolo, V. Grassi, V. Gratchev, H. M. Gray, E. Graziani, Z. D. Greenwood, K. Gregersen, I. M. Gregor, P. Grenier, J. Griffiths, A. A. Grillo, K. Grimm, S. Grinstein, Ph. Gris, J.-F. Grivaz, J. P. Grohs, A. Grohsjean, E. Gross, J. Grosse-Knetter, G. C. Grossi, Z. J. Grout, L. Guan, J. Guenther, F. Guescini, D. Guest, O. Gueta, E. Guido, T. Guillemin, S. Guindon, U. Gul, C. Gumpert, J. Guo, S. Gupta, P. Gutierrez, N. G. Gutierrez Ortiz, C. Gutschow, C. Guyot, C. Gwenlan, C. B. Gwilliam, A. Haas, C. Haber, H. K. Hadavand, N. Haddad, P. Haefner, S. Hageböck, Z. Hajduk, H. Hakobyan, M. Haleem, J. Haley, D. Hall, G. Halladjian, G. D. Hallewell, K. Hamacher, P. Hamal, K. Hamano, M. Hamer, A. Hamilton, G. N. Hamity, P. G. Hamnett, L. Han, K. Hanagaki, K. Hanawa, M. Hance, P. Hanke, R. Hanna, J. B. Hansen, J. D. Hansen, M. C. Hansen, P. H. Hansen, K. Hara, A. S. Hard, T. Harenberg, F. Hariri, S. Harkusha, R. D. Harrington, P. F. Harrison, F. Hartjes, M. Hasegawa, S. Hasegawa, Y. Hasegawa, A. Hasib, S. Hassani, S. Haug, R. Hauser, L. Hauswald, M. Havranek, C. M. Hawkes, R. J. Hawkings, A. D. Hawkins, T. Hayashi, D. Hayden, C. P. Hays, J. M. Hays, H. S. Hayward, S. J. Haywood, S. J. Head, T. Heck, V. Hedberg, L. Heelan, S. Heim, T. Heim, B. Heinemann, L. Heinrich, J. Hejbal, L. Helary, S. Hellman, D. Hellmich, C. Helsens, J. Henderson, R. C. W. Henderson, Y. Heng, C. Hengler, A. Henrichs, A. M. Henriques Correia, S. Henrot-Versille, G. H. Herbert, Y. Hernández Jiménez, R. Herrberg-Schubert, G. Herten, R. Hertenberger, L. Hervas, G. G. Hesketh, N. P. Hessey, J. W. Hetherly, R. Hickling, E. Higón-Rodriguez, E. Hill, J. C. Hill, K. H. Hiller, S. J. Hillier, I. Hinchliffe, E. Hines, R. R. Hinman, M. Hirose, D. Hirschbuehl, J. Hobbs, N. Hod, M. C. Hodgkinson, P. Hodgson, A. Hoecker, M. R. Hoeferkamp, F. Hoenig, M. Hohlfeld, D. Hohn, T. R. Holmes, M. Homann, T. M. Hong, L. Hooft van Huysduynen, W. H. Hopkins, Y. Horii, A. J. Horton, J-Y. Hostachy, S. Hou, A. Hoummada, J. Howard, J. Howarth, M. Hrabovsky, I. Hristova, J. Hrivnac, T. Hryn’ova, A. Hrynevich, C. Hsu, P. J. Hsu, S.-C. Hsu, D. Hu, Q. Hu, X. Hu, Y. Huang, Z. Hubacek, F. Hubaut, F. Huegging, T. B. Huffman, E. W. Hughes, G. Hughes, M. Huhtinen, T. A. Hülsing, N. Huseynov, J. Huston, J. Huth, G. Iacobucci, G. Iakovidis, I. Ibragimov, L. Iconomidou-Fayard, E. Ideal, Z. Idrissi, P. Iengo, O. Igonkina, T. Iizawa, Y. Ikegami, K. Ikematsu, M. Ikeno, Y. Ilchenko, D. Iliadis, N. Ilic, Y. Inamaru, T. Ince, P. Ioannou, M. Iodice, K. Iordanidou, V. Ippolito, A. Irles Quiles, C. Isaksson, M. Ishino, M. Ishitsuka, R. Ishmukhametov, C. Issever, S. Istin, J. M. Iturbe Ponce, R. Iuppa, J. Ivarsson, W. Iwanski, H. Iwasaki, J. M. Izen, V. Izzo, S. Jabbar, B. Jackson, M. Jackson, P. Jackson, M. R. Jaekel, V. Jain, K. Jakobs, S. Jakobsen, T. Jakoubek, J. Jakubek, D. O. Jamin, D. K. Jana, E. Jansen, R. W. Jansky, J. Janssen, M. Janus, G. Jarlskog, N. Javadov, T. Javůrek, L. Jeanty, J. Jejelava, G.-Y. Jeng, D. Jennens, P. Jenni, J. Jentzsch, C. Jeske, S. Jézéquel, H. Ji, J. Jia, Y. Jiang, S. Jiggins, J. Jimenez Pena, S. Jin, A. Jinaru, O. Jinnouchi, M. D. Joergensen, P. Johansson, K. A. Johns, K. Jon-And, G. Jones, R. W. L. Jones, T. J. Jones, J. Jongmanns, P. M. Jorge, K. D. Joshi, J. Jovicevic, X. Ju, C. A. Jung, P. Jussel, A. Juste Rozas, M. Kaci, A. Kaczmarska, M. Kado, H. Kagan, M. Kagan, S. J. Kahn, E. Kajomovitz, C. W. Kalderon, S. Kama, A. Kamenshchikov, N. Kanaya, M. Kaneda, S. Kaneti, V. A. Kantserov, J. Kanzaki, B. Kaplan, A. Kapliy, D. Kar, K. Karakostas, A. Karamaoun, N. Karastathis, M. J. Kareem, M. Karnevskiy, S. N. Karpov, Z. M. Karpova, K. Karthik, V. Kartvelishvili, A. N. Karyukhin, L. Kashif, R. D. Kass, A. Kastanas, Y. Kataoka, A. Katre, J. Katzy, K. Kawagoe, T. Kawamoto, G. Kawamura, S. Kazama, V. F. Kazanin, M. Y. Kazarinov, R. Keeler, R. Kehoe, J. S. Keller, J. J. Kempster, H. Keoshkerian, O. Kepka, B. P. Kerševan, S. Kersten, R. A. Keyes, F. Khalil-zada, H. Khandanyan, A. Khanov, A. G. Kharlamov, T. J. Khoo, V. Khovanskiy, E. Khramov, J. Khubua, H. Y. Kim, H. Kim, S. H. Kim, Y. Kim, N. Kimura, O. M. Kind, B. T. King, M. King, R. S. B. King, S. B. King, J. Kirk, A. E. Kiryunin, T. Kishimoto, D. Kisielewska, F. Kiss, K. Kiuchi, O. Kivernyk, E. Kladiva, M. H. Klein, M. Klein, U. Klein, K. Kleinknecht, P. Klimek, A. Klimentov, R. Klingenberg, J. A. Klinger, T. Klioutchnikova, E.-E. Kluge, P. Kluit, S. Kluth, E. Kneringer, E. B. F. G. Knoops, A. Knue, A. Kobayashi, D. Kobayashi, T. Kobayashi, M. Kobel, M. Kocian, P. Kodys, T. Koffas, E. Koffeman, L. A. Kogan, S. Kohlmann, Z. Kohout, T. Kohriki, T. Koi, H. Kolanoski, I. Koletsou, A. A. Komar, Y. Komori, T. Kondo, N. Kondrashova, K. Köneke, A. C. König, S. König, T. Kono, R. Konoplich, N. Konstantinidis, R. Kopeliansky, S. Koperny, L. Köpke, A. K. Kopp, K. Korcyl, K. Kordas, A. Korn, A. A. Korol, I. Korolkov, E. V. Korolkova, O. Kortner, S. Kortner, T. Kosek, V. V. Kostyukhin, V. M. Kotov, A. Kotwal, A. Kourkoumeli-Charalampidi, C. Kourkoumelis, V. Kouskoura, A. Koutsman, R. Kowalewski, T. Z. Kowalski, W. Kozanecki, A. S. Kozhin, V. A. Kramarenko, G. Kramberger, D. Krasnopevtsev, A. Krasznahorkay, J. K. Kraus, A. Kravchenko, S. Kreiss, M. Kretz, J. Kretzschmar, K. Kreutzfeldt, P. Krieger, K. Krizka, K. Kroeninger, H. Kroha, J. Kroll, J. Kroseberg, J. Krstic, U. Kruchonak, H. Krüger, N. Krumnack, Z. V. Krumshteyn, A. Kruse, M. C. Kruse, M. Kruskal, T. Kubota, H. Kucuk, S. Kuday, S. Kuehn, A. Kugel, F. Kuger, A. Kuhl, T. Kuhl, V. Kukhtin, Y. Kulchitsky, S. Kuleshov, M. Kuna, T. Kunigo, A. Kupco, H. Kurashige, Y. A. Kurochkin, R. Kurumida, V. Kus, E. S. Kuwertz, M. Kuze, J. Kvita, T. Kwan, D. Kyriazopoulos, A. La Rosa, J. L. La Rosa Navarro, L. La Rotonda, C. Lacasta, F. Lacava, J. Lacey, H. Lacker, D. Lacour, V. R. Lacuesta, E. Ladygin, R. Lafaye, B. Laforge, T. Lagouri, S. Lai, L. Lambourne, S. Lammers, C. L. Lampen, W. Lampl, E. Lançon, U. Landgraf, M. P. J. Landon, V. S. Lang, J. C. Lange, A. J. Lankford, F. Lanni, K. Lantzsch, S. Laplace, C. Lapoire, J. F. Laporte, T. Lari, F. Lasagni Manghi, M. Lassnig, P. Laurelli, W. Lavrijsen, A. T. Law, P. Laycock, T. Lazovich, O. Le Dortz, E. Le Guirriec, E. Le Menedeu, M. LeBlanc, T. LeCompte, F. Ledroit-Guillon, C. A. Lee, S. C. Lee, L. Lee, G. Lefebvre, M. Lefebvre, F. Legger, C. Leggett, A. Lehan, G. Lehmann Miotto, X. Lei, W. A. Leight, A. Leisos, A. G. Leister, M. A. L. Leite, R. Leitner, D. Lellouch, B. Lemmer, K. J. C. Leney, T. Lenz, B. Lenzi, R. Leone, S. Leone, C. Leonidopoulos, S. Leontsinis, C. Leroy, C. G. Lester, M. Levchenko, J. Levêque, D. Levin, L. J. Levinson, M. Levy, A. Lewis, A. M. Leyko, M. Leyton, B. Li, H. Li, H. L. Li, L. Li, L. Li, S. Li, Y. Li, Z. Liang, H. Liao, B. Liberti, A. Liblong, P. Lichard, K. Lie, J. Liebal, W. Liebig, C. Limbach, A. Limosani, S. C. Lin, T. H. Lin, F. Linde, B. E. Lindquist, J. T. Linnemann, E. Lipeles, A. Lipniacka, M. Lisovyi, T. M. Liss, D. Lissauer, A. Lister, A. M. Litke, B. Liu, D. Liu, J. Liu, J. B. Liu, K. Liu, L. Liu, M. Liu, M. Liu, Y. Liu, M. Livan, A. Lleres, J. Llorente Merino, S. L. Lloyd, F. Lo Sterzo, E. Lobodzinska, P. Loch, W. S. Lockman, F. K. Loebinger, A. E. Loevschall-Jensen, A. Loginov, T. Lohse, K. Lohwasser, M. Lokajicek, B. A. Long, J. D. Long, R. E. Long, K. A. Looper, L. Lopes, D. Lopez Mateos, B. Lopez Paredes, I. Lopez Paz, J. Lorenz, N. Lorenzo Martinez, M. Losada, P. Loscutoff, P. J. Lösel, X. Lou, A. Lounis, J. Love, P. A. Love, N. Lu, H. J. Lubatti, C. Luci, A. Lucotte, F. Luehring, W. Lukas, L. Luminari, O. Lundberg, B. Lund-Jensen, D. Lynn, R. Lysak, E. Lytken, H. Ma, L. L. Ma, G. Maccarrone, A. Macchiolo, C. M. Macdonald, J. Machado Miguens, D. Macina, D. Madaffari, R. Madar, H. J. Maddocks, W. F. Mader, A. Madsen, S. Maeland, T. Maeno, A. Maevskiy, E. Magradze, K. Mahboubi, J. Mahlstedt, C. Maiani, C. Maidantchik, A. A. Maier, T. Maier, A. Maio, S. Majewski, Y. Makida, N. Makovec, B. Malaescu, Pa. Malecki, V. P. Maleev, F. Malek, U. Mallik, D. Malon, C. Malone, S. Maltezos, V. M. Malyshev, S. Malyukov, J. Mamuzic, G. Mancini, B. Mandelli, L. Mandelli, I. Mandić, R. Mandrysch, J. Maneira, A. Manfredini, L. Manhaes de Andrade Filho, J. Manjarres Ramos, A. Mann, P. M. Manning, A. Manousakis-Katsikakis, B. Mansoulie, R. Mantifel, M. Mantoani, L. Mapelli, L. March, G. Marchiori, M. Marcisovsky, C. P. Marino, M. Marjanovic, F. Marroquim, S. P. Marsden, Z. Marshall, L. F. Marti, S. Marti-Garcia, B. Martin, T. A. Martin, V. J. Martin, B. Martin dit Latour, M. Martinez, S. Martin-Haugh, V. S. Martoiu, A. C. Martyniuk, M. Marx, F. Marzano, A. Marzin, L. Masetti, T. Mashimo, R. Mashinistov, J. Masik, A. L. Maslennikov, I. Massa, L. Massa, N. Massol, P. Mastrandrea, A. Mastroberardino, T. Masubuchi, P. Mättig, J. Mattmann, J. Maurer, S. J. Maxfield, D. A. Maximov, R. Mazini, S. M. Mazza, L. Mazzaferro, G. Mc Goldrick, S. P. Mc Kee, A. McCarn, R. L. McCarthy, T. G. McCarthy, N. A. McCubbin, K. W. McFarlane, J. A. Mcfayden, G. Mchedlidze, S. J. McMahon, R. A. McPherson, M. Medinnis, S. Meehan, S. Mehlhase, A. Mehta, K. Meier, C. Meineck, B. Meirose, B. R. Mellado Garcia, F. Meloni, A. Mengarelli, S. Menke, E. Meoni, K. M. Mercurio, S. Mergelmeyer, P. Mermod, L. Merola, C. Meroni, F. S. Merritt, A. Messina, J. Metcalfe, A. S. Mete, C. Meyer, C. Meyer, J-P. Meyer, J. Meyer, R. P. Middleton, S. Miglioranzi, L. Mijović, G. Mikenberg, M. Mikestikova, M. Mikuž, M. Milesi, A. Milic, D. W. Miller, C. Mills, A. Milov, D. A. Milstead, A. A. Minaenko, Y. Minami, I. A. Minashvili, A. I. Mincer, B. Mindur, M. Mineev, Y. Ming, L. M. Mir, T. Mitani, J. Mitrevski, V. A. Mitsou, A. Miucci, P. S. Miyagawa, J. U. Mjörnmark, T. Moa, K. Mochizuki, S. Mohapatra, W. Mohr, S. Molander, R. Moles-Valls, K. Mönig, C. Monini, J. Monk, E. Monnier, J. Montejo Berlingen, F. Monticelli, S. Monzani, R. W. Moore, N. Morange, D. Moreno, M. Moreno Llácer, P. Morettini, M. Morgenstern, M. Morii, M. Morinaga, V. Morisbak, S. Moritz, A. K. Morley, G. Mornacchi, J. D. Morris, S. S. Mortensen, A. Morton, L. Morvaj, M. Mosidze, J. Moss, K. Motohashi, R. Mount, E. Mountricha, S. V. Mouraviev, E. J. W. Moyse, S. Muanza, R. D. Mudd, F. Mueller, J. Mueller, K. Mueller, R. S. P. Mueller, T. Mueller, D. Muenstermann, P. Mullen, Y. Munwes, J. A. Murillo Quijada, W. J. Murray, H. Musheghyan, E. Musto, A. G. Myagkov, M. Myska, O. Nackenhorst, J. Nadal, K. Nagai, R. Nagai, Y. Nagai, K. Nagano, A. Nagarkar, Y. Nagasaka, K. Nagata, M. Nagel, E. Nagy, A. M. Nairz, Y. Nakahama, K. Nakamura, T. Nakamura, I. Nakano, H. Namasivayam, R. F. Naranjo Garcia, R. Narayan, T. Naumann, G. Navarro, R. Nayyar, H. A. Neal, P. Yu. Nechaeva, T. J. Neep, P. D. Nef, A. Negri, M. Negrini, S. Nektarijevic, C. Nellist, A. Nelson, S. Nemecek, P. Nemethy, A. A. Nepomuceno, M. Nessi, M. S. Neubauer, M. Neumann, R. M. Neves, P. Nevski, P. R. Newman, D. H. Nguyen, R. B. Nickerson, R. Nicolaidou, B. Nicquevert, J. Nielsen, N. Nikiforou, A. Nikiforov, V. Nikolaenko, I. Nikolic-Audit, K. Nikolopoulos, J. K. Nilsen, P. Nilsson, Y. Ninomiya, A. Nisati, R. Nisius, T. Nobe, M. Nomachi, I. Nomidis, T. Nooney, S. Norberg, M. Nordberg, O. Novgorodova, S. Nowak, M. Nozaki, L. Nozka, K. Ntekas, G. Nunes Hanninger, T. Nunnemann, E. Nurse, F. Nuti, B. J. O’Brien, F. O’grady, D. C. O’Neil, V. O’Shea, F. G. Oakham, H. Oberlack, T. Obermann, J. Ocariz, A. Ochi, I. Ochoa, J. P. Ochoa-Ricoux, S. Oda, S. Odaka, H. Ogren, A. Oh, S. H. Oh, C. C. Ohm, H. Ohman, H. Oide, W. Okamura, H. Okawa, Y. Okumura, T. Okuyama, A. Olariu, S. A. Olivares Pino, D. Oliveira Damazio, E. Oliver Garcia, A. Olszewski, J. Olszowska, A. Onofre, P. U. E. Onyisi, C. J. Oram, M. J. Oreglia, Y. Oren, D. Orestano, N. Orlando, C. Oropeza Barrera, R. S. Orr, B. Osculati, R. Ospanov, G. Otero y Garzon, H. Otono, M. Ouchrif, E. A. Ouellette, F. Ould-Saada, A. Ouraou, K. P. Oussoren, Q. Ouyang, A. Ovcharova, M. Owen, R. E. Owen, V. E. Ozcan, N. Ozturk, K. Pachal, A. Pacheco Pages, C. Padilla Aranda, M. Pagáčová, S. Pagan Griso, E. Paganis, C. Pahl, F. Paige, P. Pais, K. Pajchel, G. Palacino, S. Palestini, M. Palka, D. Pallin, A. Palma, Y. B. Pan, E. Panagiotopoulou, C. E. Pandini, J. G. Panduro Vazquez, P. Pani, S. Panitkin, D. Pantea, L. Paolozzi, Th. D. Papadopoulou, K. Papageorgiou, A. Paramonov, D. Paredes Hernandez, M. A. Parker, K. A. Parker, F. Parodi, J. A. Parsons, U. Parzefall, E. Pasqualucci, S. Passaggio, F. Pastore, Fr. Pastore, G. Pásztor, S. Pataraia, N. D. Patel, J. R. Pater, T. Pauly, J. Pearce, B. Pearson, L. E. Pedersen, M. Pedersen, S. Pedraza Lopez, R. Pedro, S. V. Peleganchuk, D. Pelikan, H. Peng, B. Penning, J. Penwell, D. V. Perepelitsa, E. Perez Codina, M. T. Pérez García-Estañ, L. Perini, H. Pernegger, S. Perrella, R. Peschke, V. D. Peshekhonov, K. Peters, R. F. Y. Peters, B. A. Petersen, T. C. Petersen, E. Petit, A. Petridis, C. Petridou, E. Petrolo, F. Petrucci, N. E. Pettersson, R. Pezoa, P. W. Phillips, G. Piacquadio, E. Pianori, A. Picazio, E. Piccaro, M. Piccinini, M. A. Pickering, R. Piegaia, D. T. Pignotti, J. E. Pilcher, A. D. Pilkington, J. Pina, M. Pinamonti, J. L. Pinfold, A. Pingel, B. Pinto, S. Pires, M. Pitt, C. Pizio, L. Plazak, M.-A. Pleier, V. Pleskot, E. Plotnikova, P. Plucinski, D. Pluth, R. Poettgen, L. Poggioli, D. Pohl, G. Polesello, A. Policicchio, R. Polifka, A. Polini, C. S. Pollard, V. Polychronakos, K. Pommès, L. Pontecorvo, B. G. Pope, G. A. Popeneciu, D. S. Popovic, A. Poppleton, S. Pospisil, K. Potamianos, I. N. Potrap, C. J. Potter, C. T. Potter, G. Poulard, J. Poveda, V. Pozdnyakov, P. Pralavorio, A. Pranko, S. Prasad, S. Prell, D. Price, L. E. Price, M. Primavera, S. Prince, M. Proissl, K. Prokofiev, F. Prokoshin, E. Protopapadaki, S. Protopopescu, J. Proudfoot, M. Przybycien, E. Ptacek, D. Puddu, E. Pueschel, D. Puldon, M. Purohit, P. Puzo, J. Qian, G. Qin, Y. Qin, A. Quadt, D. R. Quarrie, W. B. Quayle, M. Queitsch-Maitland, D. Quilty, S. Raddum, V. Radeka, V. Radescu, S. K. Radhakrishnan, P. Radloff, P. Rados, F. Ragusa, G. Rahal, S. Rajagopalan, M. Rammensee, C. Rangel-Smith, F. Rauscher, S. Rave, T. Ravenscroft, M. Raymond, A. L. Read, N. P. Readioff, D. M. Rebuzzi, A. Redelbach, G. Redlinger, R. Reece, K. Reeves, L. Rehnisch, H. Reisin, M. Relich, C. Rembser, H. Ren, A. Renaud, M. Rescigno, S. Resconi, O. L. Rezanova, P. Reznicek, R. Rezvani, R. Richter, S. Richter, E. Richter-Was, O. Ricken, M. Ridel, P. Rieck, C. J. Riegel, J. Rieger, M. Rijssenbeek, A. Rimoldi, L. Rinaldi, B. Ristić, E. Ritsch, I. Riu, F. Rizatdinova, E. Rizvi, S. H. Robertson, A. Robichaud-Veronneau, D. Robinson, J. E. M. Robinson, A. Robson, C. Roda, S. Roe, O. Røhne, S. Rolli, A. Romaniouk, M. Romano, S. M. Romano Saez, E. Romero Adam, N. Rompotis, M. Ronzani, L. Roos, E. Ros, S. Rosati, K. Rosbach, P. Rose, P. L. Rosendahl, O. Rosenthal, V. Rossetti, E. Rossi, L. P. Rossi, R. Rosten, M. Rotaru, I. Roth, J. Rothberg, D. Rousseau, C. R. Royon, A. Rozanov, Y. Rozen, X. Ruan, F. Rubbo, I. Rubinskiy, V. I. Rud, C. Rudolph, M. S. Rudolph, F. Rühr, A. Ruiz-Martinez, Z. Rurikova, N. A. Rusakovich, A. Ruschke, H. L. Russell, J. P. Rutherfoord, N. Ruthmann, Y. F. Ryabov, M. Rybar, G. Rybkin, N. C. Ryder, A. F. Saavedra, G. Sabato, S. Sacerdoti, A. Saddique, H. F-W. Sadrozinski, R. Sadykov, F. Safai Tehrani, M. Saimpert, H. Sakamoto, Y. Sakurai, G. Salamanna, A. Salamon, M. Saleem, D. Salek, P. H. Sales De Bruin, D. Salihagic, A. Salnikov, J. Salt, D. Salvatore, F. Salvatore, A. Salvucci, A. Salzburger, D. Sampsonidis, A. Sanchez, J. Sánchez, V. Sanchez Martinez, H. Sandaker, R. L. Sandbach, H. G. Sander, M. P. Sanders, M. Sandhoff, C. Sandoval, R. Sandstroem, D. P. C. Sankey, M. Sannino, A. Sansoni, C. Santoni, R. Santonico, H. Santos, I. Santoyo Castillo, K. Sapp, A. Sapronov, J. G. Saraiva, B. Sarrazin, O. Sasaki, Y. Sasaki, K. Sato, G. Sauvage, E. Sauvan, G. Savage, P. Savard, C. Sawyer, L. Sawyer, J. Saxon, C. Sbarra, A. Sbrizzi, T. Scanlon, D. A. Scannicchio, M. Scarcella, V. Scarfone, J. Schaarschmidt, P. Schacht, D. Schaefer, R. Schaefer, J. Schaeffer, S. Schaepe, S. Schaetzel, U. Schäfer, A. C. Schaffer, D. Schaile, R. D. Schamberger, V. Scharf, V. A. Schegelsky, D. Scheirich, M. Schernau, C. Schiavi, C. Schillo, M. Schioppa, S. Schlenker, E. Schmidt, K. Schmieden, C. Schmitt, S. Schmitt, S. Schmitt, B. Schneider, Y. J. Schnellbach, U. Schnoor, L. Schoeffel, A. Schoening, B. D. Schoenrock, E. Schopf, A. L. S. Schorlemmer, M. Schott, D. Schouten, J. Schovancova, S. Schramm, M. Schreyer, C. Schroeder, N. Schuh, M. J. Schultens, H.-C. Schultz-Coulon, H. Schulz, M. Schumacher, B. A. Schumm, Ph. Schune, C. Schwanenberger, A. Schwartzman, T. A. Schwarz, Ph. Schwegler, H. Schweiger, Ph. Schwemling, R. Schwienhorst, J. Schwindling, T. Schwindt, M. Schwoerer, F. G. Sciacca, E. Scifo, G. Sciolla, F. Scuri, F. Scutti, J. Searcy, G. Sedov, E. Sedykh, P. Seema, S. C. Seidel, A. Seiden, F. Seifert, J. M. Seixas, G. Sekhniaidze, K. Sekhon, S. J. Sekula, K. E. Selbach, D. M. Seliverstov, N. Semprini-Cesari, C. Serfon, L. Serin, L. Serkin, T. Serre, M. Sessa, R. Seuster, H. Severini, T. Sfiligoj, F. Sforza, A. Sfyrla, E. Shabalina, M. Shamim, L. Y. Shan, R. Shang, J. T. Shank, M. Shapiro, P. B. Shatalov, K. Shaw, S. M. Shaw, A. Shcherbakova, C. Y. Shehu, P. Sherwood, L. Shi, S. Shimizu, C. O. Shimmin, M. Shimojima, M. Shiyakova, A. Shmeleva, D. Shoaleh Saadi, M. J. Shochet, S. Shojaii, S. Shrestha, E. Shulga, M. A. Shupe, S. Shushkevich, P. Sicho, O. Sidiropoulou, D. Sidorov, A. Sidoti, F. Siegert, Dj. Sijacki, J. Silva, Y. Silver, S. B. Silverstein, V. Simak, O. Simard, Lj. Simic, S. Simion, E. Simioni, B. Simmons, D. Simon, R. Simoniello, P. Sinervo, N. B. Sinev, G. Siragusa, A. N. Sisakyan, S. Yu. Sivoklokov, J. Sjölin, T. B. Sjursen, M. B. Skinner, H. P. Skottowe, P. Skubic, M. Slater, T. Slavicek, M. Slawinska, K. Sliwa, V. Smakhtin, B. H. Smart, L. Smestad, S. Yu. Smirnov, Y. Smirnov, L. N. Smirnova, O. Smirnova, M. N. K. Smith, R. W. Smith, M. Smizanska, K. Smolek, A. A. Snesarev, G. Snidero, S. Snyder, R. Sobie, F. Socher, A. Soffer, D. A. Soh, C. A. Solans, M. Solar, J. Solc, E. Yu. Soldatov, U. Soldevila, A. A. Solodkov, A. Soloshenko, O. V. Solovyanov, V. Solovyev, P. Sommer, H. Y. Song, N. Soni, A. Sood, A. Sopczak, B. Sopko, V. Sopko, V. Sorin, D. Sosa, M. Sosebee, C. L. Sotiropoulou, R. Soualah, P. Soueid, A. M. Soukharev, D. South, B. C. Sowden, S. Spagnolo, M. Spalla, F. Spanò, W. R. Spearman, F. Spettel, R. Spighi, G. Spigo, L. A. Spiller, M. Spousta, T. Spreitzer, R. D. St. Denis, S. Staerz, J. Stahlman, R. Stamen, S. Stamm, E. Stanecka, C. Stanescu, M. Stanescu-Bellu, M. M. Stanitzki, S. Stapnes, E. A. Starchenko, J. Stark, P. Staroba, P. Starovoitov, R. Staszewski, P. Stavina, P. Steinberg, B. Stelzer, H. J. Stelzer, O. Stelzer-Chilton, H. Stenzel, S. Stern, G. A. Stewart, J. A. Stillings, M. C. Stockton, M. Stoebe, G. Stoicea, P. Stolte, S. Stonjek, A. R. Stradling, A. Straessner, M. E. Stramaglia, J. Strandberg, S. Strandberg, A. Strandlie, E. Strauss, M. Strauss, P. Strizenec, R. Ströhmer, D. M. Strom, R. Stroynowski, A. Strubig, S. A. Stucci, B. Stugu, N. A. Styles, D. Su, J. Su, R. Subramaniam, A. Succurro, Y. Sugaya, C. Suhr, M. Suk, V. V. Sulin, S. Sultansoy, T. Sumida, S. Sun, X. Sun, J. E. Sundermann, K. Suruliz, G. Susinno, M. R. Sutton, S. Suzuki, Y. Suzuki, M. Svatos, S. Swedish, M. Swiatlowski, I. Sykora, T. Sykora, D. Ta, C. Taccini, K. Tackmann, J. Taenzer, A. Taffard, R. Tafirout, N. Taiblum, H. Takai, R. Takashima, H. Takeda, T. Takeshita, Y. Takubo, M. Talby, A. A. Talyshev, J. Y. C. Tam, K. G. Tan, J. Tanaka, R. Tanaka, S. Tanaka, B. B. Tannenwald, N. Tannoury, S. Tapprogge, S. Tarem, F. Tarrade, G. F. Tartarelli, P. Tas, M. Tasevsky, T. Tashiro, E. Tassi, A. Tavares Delgado, Y. Tayalati, F. E. Taylor, G. N. Taylor, W. Taylor, F. A. Teischinger, M. Teixeira Dias Castanheira, P. Teixeira-Dias, K. K. Temming, H. Ten Kate, P. K. Teng, J. J. Teoh, F. Tepel, S. Terada, K. Terashi, J. Terron, S. Terzo, M. Testa, R. J. Teuscher, J. Therhaag, T. Theveneaux-Pelzer, J. P. Thomas, J. Thomas-Wilsker, E. N. Thompson, P. D. Thompson, R. J. Thompson, A. S. Thompson, L. A. Thomsen, E. Thomson, M. Thomson, R. P. Thun, M. J. Tibbetts, R. E. Ticse Torres, V. O. Tikhomirov, Yu. A. Tikhonov, S. Timoshenko, E. Tiouchichine, P. Tipton, S. Tisserant, T. Todorov, S. Todorova-Nova, J. Tojo, S. Tokár, K. Tokushuku, K. Tollefson, E. Tolley, L. Tomlinson, M. Tomoto, L. Tompkins, K. Toms, E. Torrence, H. Torres, E. Torró Pastor, J. Toth, F. Touchard, D. R. Tovey, T. Trefzger, L. Tremblet, A. Tricoli, I. M. Trigger, S. Trincaz-Duvoid, M. F. Tripiana, W. Trischuk, B. Trocmé, C. Troncon, M. Trottier-McDonald, M. Trovatelli, P. True, L. Truong, M. Trzebinski, A. Trzupek, C. Tsarouchas, J. C-L. Tseng, P. V. Tsiareshka, D. Tsionou, G. Tsipolitis, N. Tsirintanis, S. Tsiskaridze, V. Tsiskaridze, E. G. Tskhadadze, I. I. Tsukerman, V. Tsulaia, S. Tsuno, D. Tsybychev, A. Tudorache, V. Tudorache, A. N. Tuna, S. A. Tupputi, S. Turchikhin, D. Turecek, R. Turra, A. J. Turvey, P. M. Tuts, A. Tykhonov, M. Tylmad, M. Tyndel, I. Ueda, R. Ueno, M. Ughetto, M. Ugland, M. Uhlenbrock, F. Ukegawa, G. Unal, A. Undrus, G. Unel, F. C. Ungaro, Y. Unno, C. Unverdorben, J. Urban, P. Urquijo, P. Urrejola, G. Usai, A. Usanova, L. Vacavant, V. Vacek, B. Vachon, C. Valderanis, N. Valencic, S. Valentinetti, A. Valero, L. Valery, S. Valkar, E. Valladolid Gallego, S. Vallecorsa, J. A. Valls Ferrer, W. Van Den Wollenberg, P. C. Van Der Deijl, R. van der Geer, H. van der Graaf, R. Van Der Leeuw, N. van Eldik, P. van Gemmeren, J. Van Nieuwkoop, I. van Vulpen, M. C. van Woerden, M. Vanadia, W. Vandelli, R. Vanguri, A. Vaniachine, F. Vannucci, G. Vardanyan, R. Vari, E. W. Varnes, T. Varol, D. Varouchas, A. Vartapetian, K. E. Varvell, F. Vazeille, T. Vazquez Schroeder, J. Veatch, L. M. Veloce, F. Veloso, T. Velz, S. Veneziano, A. Ventura, D. Ventura, M. Venturi, N. Venturi, A. Venturini, V. Vercesi, M. Verducci, W. Verkerke, J. C. Vermeulen, A. Vest, M. C. Vetterli, O. Viazlo, I. Vichou, T. Vickey, O. E. Vickey Boeriu, G. H. A. Viehhauser, S. Viel, R. Vigne, M. Villa, M. Villaplana Perez, E. Vilucchi, M. G. Vincter, V. B. Vinogradov, I. Vivarelli, F. Vives Vaque, S. Vlachos, D. Vladoiu, M. Vlasak, M. Vogel, P. Vokac, G. Volpi, M. Volpi, H. von der Schmitt, H. von Radziewski, E. von Toerne, V. Vorobel, K. Vorobev, M. Vos, R. Voss, J. H. Vossebeld, N. Vranjes, M. Vranjes Milosavljevic, V. Vrba, M. Vreeswijk, R. Vuillermet, I. Vukotic, Z. Vykydal, P. Wagner, W. Wagner, H. Wahlberg, S. Wahrmund, J. Wakabayashi, J. Walder, R. Walker, W. Walkowiak, C. Wang, F. Wang, H. Wang, H. Wang, J. Wang, J. Wang, K. Wang, R. Wang, S. M. Wang, T. Wang, X. Wang, C. Wanotayaroj, A. Warburton, C. P. Ward, D. R. Wardrope, M. Warsinsky, A. Washbrook, C. Wasicki, P. M. Watkins, A. T. Watson, I. J. Watson, M. F. Watson, G. Watts, S. Watts, B. M. Waugh, S. Webb, M. S. Weber, S. W. Weber, J. S. Webster, A. R. Weidberg, B. Weinert, J. Weingarten, C. Weiser, H. Weits, P. S. Wells, T. Wenaus, T. Wengler, S. Wenig, N. Wermes, M. Werner, P. Werner, M. Wessels, J. Wetter, K. Whalen, A. M. Wharton, A. White, M. J. White, R. White, S. White, D. Whiteson, F. J. Wickens, W. Wiedenmann, M. Wielers, P. Wienemann, C. Wiglesworth, L. A. M. Wiik-Fuchs, A. Wildauer, H. G. Wilkens, H. H. Williams, S. Williams, C. Willis, S. Willocq, A. Wilson, J. A. Wilson, I. Wingerter-Seez, F. Winklmeier, B. T. Winter, M. Wittgen, J. Wittkowski, S. J. Wollstadt, M. W. Wolter, H. Wolters, B. K. Wosiek, J. Wotschack, M. J. Woudstra, K. W. Wozniak, M. Wu, M. Wu, S. L. Wu, X. Wu, Y. Wu, T. R. Wyatt, B. M. Wynne, S. Xella, D. Xu, L. Xu, B. Yabsley, S. Yacoob, R. Yakabe, M. Yamada, Y. Yamaguchi, A. Yamamoto, S. Yamamoto, T. Yamanaka, K. Yamauchi, Y. Yamazaki, Z. Yan, H. Yang, H. Yang, Y. Yang, L. Yao, W-M. Yao, Y. Yasu, E. Yatsenko, K. H. Yau Wong, J. Ye, S. Ye, I. Yeletskikh, A. L. Yen, E. Yildirim, K. Yorita, R. Yoshida, K. Yoshihara, C. Young, C. J. S. Young, S. Youssef, D. R. Yu, J. Yu, J. M. Yu, J. Yu, L. Yuan, A. Yurkewicz, I. Yusuff, B. Zabinski, R. Zaidan, A. M. Zaitsev, J. Zalieckas, A. Zaman, S. Zambito, L. Zanello, D. Zanzi, C. Zeitnitz, M. Zeman, A. Zemla, K. Zengel, O. Zenin, T. Ženiš, D. Zerwas, D. Zhang, F. Zhang, J. Zhang, L. Zhang, R. Zhang, X. Zhang, Z. Zhang, X. Zhao, Y. Zhao, Z. Zhao, A. Zhemchugov, J. Zhong, B. Zhou, C. Zhou, L. Zhou, L. Zhou, N. Zhou, C. G. Zhu, H. Zhu, J. Zhu, Y. Zhu, X. Zhuang, K. Zhukov, A. Zibell, D. Zieminska, N. I. Zimine, C. Zimmermann, S. Zimmermann, Z. Zinonos, M. Zinser, M. Ziolkowski, L. Živković, G. Zobernig, A. Zoccoli, M. zur Nedden, G. Zurzolo, L. Zwalinski, ATLAS Publications

**Affiliations:** Department of Physics, University of Adelaide, Adelaide, Australia; Physics Department, SUNY Albany, Albany, NY USA; Department of Physics, University of Alberta, Edmonton, AB Canada; Department of Physics, Ankara University, Ankara, Turkey; Istanbul Aydin University, Istanbul, Turkey; Division of Physics, TOBB University of Economics and Technology, Ankara, Turkey; LAPP, CNRS/IN2P3 and Université Savoie Mont Blanc, Annecy-le-Vieux, France; High Energy Physics Division, Argonne National Laboratory, Argonne, IL USA; Department of Physics, University of Arizona, Tucson, AZ USA; Department of Physics, The University of Texas at Arlington, Arlington, TX USA; Physics Department, University of Athens, Athens, Greece; Physics Department, National Technical University of Athens, Zografou, Greece; Institute of Physics, Azerbaijan Academy of Sciences, Baku, Azerbaijan; Institut de Física d’Altes Energies and Departament de Física de la Universitat Autònoma de Barcelona, Barcelona, Spain; Institute of Physics, University of Belgrade, Belgrade, Serbia; Department for Physics and Technology, University of Bergen, Bergen, Norway; Physics Division, Lawrence Berkeley National Laboratory and University of California, Berkeley, CA USA; Department of Physics, Humboldt University, Berlin, Germany; Albert Einstein Center for Fundamental Physics and Laboratory for High Energy Physics, University of Bern, Bern, Switzerland; School of Physics and Astronomy, University of Birmingham, Birmingham, UK; Department of Physics, Bogazici University, Istanbul, Turkey; Department of Physics, Dogus University, Istanbul, Turkey; Department of Physics Engineering, Gaziantep University, Gaziantep, Turkey; INFN Sezione di Bologna, Bologna, Italy; Dipartimento di Fisica e Astronomia, Università di Bologna, Bologna, Italy; Physikalisches Institut, University of Bonn, Bonn, Germany; Department of Physics, Boston University, Boston, MA USA; Department of Physics, Brandeis University, Waltham, MA USA; Universidade Federal do Rio De Janeiro COPPE/EE/IF, Rio de Janeiro, Brazil; Electrical Circuits Department, Federal University of Juiz de Fora (UFJF), Juiz de Fora, Brazil; Federal University of Sao Joao del Rei (UFSJ), Sao Joao del Rei, Brazil; Instituto de Fisica, Universidade de Sao Paulo, São Paulo, Brazil; Physics Department, Brookhaven National Laboratory, Upton, NY USA; National Institute of Physics and Nuclear Engineering, Bucharest, Romania; Physics Department, National Institute for Research and Development of Isotopic and Molecular Technologies, Cluj Napoca, Romania; University Politehnica Bucharest, Bucharest, Romania; West University in Timisoara, Timisoara, Romania; Departamento de Física, Universidad de Buenos Aires, Buenos Aires, Argentina; Cavendish Laboratory, University of Cambridge, Cambridge, UK; Department of Physics, Carleton University, Ottawa, ON Canada; CERN, Geneva, Switzerland; Enrico Fermi Institute, University of Chicago, Chicago, IL USA; Departamento de Física, Pontificia Universidad Católica de Chile, Santiago, Chile; Departamento de Física, Universidad Técnica Federico Santa María, Valparaiso, Chile; Institute of High Energy Physics, Chinese Academy of Sciences, Beijing, China; Department of Modern Physics, University of Science and Technology of China, Hefei, Anhui China; Department of Physics, Nanjing University, Nanjing, Jiangsu China; School of Physics, Shandong University, Shandong, China; Department of Physics and Astronomy, Shanghai Key Laboratory for Particle Physics and Cosmology, Shanghai Jiao Tong University, Shanghai, China; Physics Department, Tsinghua University, Beijing, 100084 China; Laboratoire de Physique Corpusculaire, Clermont Université and Université Blaise Pascal and CNRS/IN2P3, Clermont-Ferrand, France; Nevis Laboratory, Columbia University, Irvington, NY USA; Niels Bohr Institute, University of Copenhagen, Copenhagen, Denmark; INFN Gruppo Collegato di Cosenza, Laboratori Nazionali di Frascati, Frascati, Italy; Dipartimento di Fisica, Università della Calabria, Rende, Italy; AGH University of Science and Technology, Faculty of Physics and Applied Computer Science, Kraków, Poland; Marian Smoluchowski Institute of Physics, Jagiellonian University, Kraków, Poland; Institute of Nuclear Physics, Polish Academy of Sciences, Kraków, Poland; Physics Department, Southern Methodist University, Dallas, TX USA; Physics Department, University of Texas at Dallas, Richardson, TX USA; DESY, Hamburg and Zeuthen, Germany; Institut für Experimentelle Physik IV, Technische Universität Dortmund, Dortmund, Germany; Institut für Kern- und Teilchenphysik, Technische Universität Dresden, Dresden, Germany; Department of Physics, Duke University, Durham, NC USA; SUPA-School of Physics and Astronomy, University of Edinburgh, Edinburgh, UK; INFN Laboratori Nazionali di Frascati, Frascati, Italy; Fakultät für Mathematik und Physik, Albert-Ludwigs-Universität, Freiburg, Germany; Section de Physique, Université de Genève, Geneva, Switzerland; INFN Sezione di Genova, Genoa, Italy; Dipartimento di Fisica, Università di Genova, Genoa, Italy; E. Andronikashvili Institute of Physics, Iv. Javakhishvili Tbilisi State University, Tbilisi, Georgia; High Energy Physics Institute, Tbilisi State University, Tbilisi, Georgia; II Physikalisches Institut, Justus-Liebig-Universität Giessen, Giessen, Germany; SUPA-School of Physics and Astronomy, University of Glasgow, Glasgow, UK; II Physikalisches Institut, Georg-August-Universität, Göttingen, Germany; Laboratoire de Physique Subatomique et de Cosmologie, Université Grenoble-Alpes, CNRS/IN2P3, Grenoble, France; Department of Physics, Hampton University, Hampton, VA USA; Laboratory for Particle Physics and Cosmology, Harvard University, Cambridge, MA USA; Kirchhoff-Institut für Physik, Ruprecht-Karls-Universität Heidelberg, Heidelberg, Germany; Physikalisches Institut, Ruprecht-Karls-Universität Heidelberg, Heidelberg, Germany; ZITI Institut für technische Informatik, Ruprecht-Karls-Universität Heidelberg, Mannheim, Germany; Faculty of Applied Information Science, Hiroshima Institute of Technology, Hiroshima, Japan; Department of Physics, The Chinese University of Hong Kong, Shatin, NT Hong Kong; Department of Physics, The University of Hong Kong, Hong Kong, Hong Kong; Department of Physics, The Hong Kong University of Science and Technology, Clear Water Bay, Kowloon, Hong Kong, China; Department of Physics, Indiana University, Bloomington, IN USA; Institut für Astro- und Teilchenphysik, Leopold-Franzens-Universität, Innsbruck, Austria; University of Iowa, Iowa City, IA USA; Department of Physics and Astronomy, Iowa State University, Ames, IA USA; Joint Institute for Nuclear Research, JINR Dubna, Dubna, Russia; KEK, High Energy Accelerator Research Organization, Tsukuba, Japan; Graduate School of Science, Kobe University, Kobe, Japan; Faculty of Science, Kyoto University, Kyoto, Japan; Kyoto University of Education, Kyoto, Japan; Department of Physics, Kyushu University, Fukuoka, Japan; Instituto de Física La Plata, Universidad Nacional de La Plata and CONICET, La Plata, Argentina; Physics Department, Lancaster University, Lancaster, UK; INFN Sezione di Lecce, Lecce, Italy; Dipartimento di Matematica e Fisica, Università del Salento, Lecce, Italy; Oliver Lodge Laboratory, University of Liverpool, Liverpool, UK; Department of Physics, Jožef Stefan Institute and University of Ljubljana, Ljubljana, Slovenia; School of Physics and Astronomy, Queen Mary University of London, London, UK; Department of Physics, Royal Holloway University of London, Surrey, UK; Department of Physics and Astronomy, University College London, London, UK; Louisiana Tech University, Ruston, LA USA; Laboratoire de Physique Nucléaire et de Hautes Energies, UPMC and Université Paris-Diderot and CNRS/IN2P3, Paris, France; Fysiska institutionen, Lunds universitet, Lund, Sweden; Departamento de Fisica Teorica C-15, Universidad Autonoma de Madrid, Madrid, Spain; Institut für Physik, Universität Mainz, Mainz, Germany; School of Physics and Astronomy, University of Manchester, Manchester, UK; CPPM, Aix-Marseille Université and CNRS/IN2P3, Marseille, France; Department of Physics, University of Massachusetts, Amherst, MA USA; Department of Physics, McGill University, Montreal, QC Canada; School of Physics, University of Melbourne, Melbourne, VIC Australia; Department of Physics, The University of Michigan, Ann Arbor, MI USA; Department of Physics and Astronomy, Michigan State University, East Lansing, MI USA; INFN Sezione di Milano, Milan, Italy; Dipartimento di Fisica, Università di Milano, Milan, Italy; B.I. Stepanov Institute of Physics, National Academy of Sciences of Belarus, Minsk, Republic of Belarus; National Scientific and Educational Centre for Particle and High Energy Physics, Minsk, Republic of Belarus; Department of Physics, Massachusetts Institute of Technology, Cambridge, MA USA; Group of Particle Physics, University of Montreal, Montreal, QC Canada; P.N. Lebedev Institute of Physics, Academy of Sciences, Moscow, Russia; Institute for Theoretical and Experimental Physics (ITEP), Moscow, Russia; National Research Nuclear University MEPhI, Moscow, Russia; D.V. Skobeltsyn Institute of Nuclear Physics, M.V. Lomonosov Moscow State University, Moscow, Russia; Fakultät für Physik, Ludwig-Maximilians-Universität München, Munich, Germany; Max-Planck-Institut für Physik (Werner-Heisenberg-Institut), Munich, Germany; Nagasaki Institute of Applied Science, Nagasaki, Japan; Graduate School of Science and Kobayashi-Maskawa Institute, Nagoya University, Nagoya, Japan; INFN Sezione di Napoli, Naples, Italy; Dipartimento di Fisica, Università di Napoli, Naples, Italy; Department of Physics and Astronomy, University of New Mexico, Albuquerque, NM USA; Institute for Mathematics, Astrophysics and Particle Physics, Radboud University Nijmegen/Nikhef, Nijmegen, The Netherlands; Nikhef National Institute for Subatomic Physics and University of Amsterdam, Amsterdam, The Netherlands; Department of Physics, Northern Illinois University, De Kalb, IL USA; Budker Institute of Nuclear Physics, SB RAS, Novosibirsk, Russia; Department of Physics, New York University, New York, NY USA; Ohio State University, Columbus, OH USA; Faculty of Science, Okayama University, Okayama, Japan; Homer L. Dodge Department of Physics and Astronomy, University of Oklahoma, Norman, OK USA; Department of Physics, Oklahoma State University, Stillwater, OK USA; Palacký University, RCPTM, Olomouc, Czech Republic; Center for High Energy Physics, University of Oregon, Eugene, OR USA; LAL, Université Paris-Sud and CNRS/IN2P3, Orsay, France; Graduate School of Science, Osaka University, Osaka, Japan; Department of Physics, University of Oslo, Oslo, Norway; Department of Physics, Oxford University, Oxford, UK; INFN Sezione di Pavia, Pavia, Italy; Dipartimento di Fisica, Università di Pavia, Pavia, Italy; Department of Physics, University of Pennsylvania, Philadelphia, PA USA; National Research Centre “Kurchatov Institute” B.P. Konstantinov, Petersburg Nuclear Physics Institute, St. Petersburg, Russia; INFN Sezione di Pisa, Pisa, Italy; Dipartimento di Fisica E. Fermi, Università di Pisa, Pisa, Italy; Department of Physics and Astronomy, University of Pittsburgh, Pittsburgh, PA USA; Laboratório de Instrumentação e Física Experimental de Partículas-LIP, Lisbon, Portugal; Faculdade de Ciências, Universidade de Lisboa, Lisbon, Portugal; Department of Physics, University of Coimbra, Coimbra, Portugal; Centro de Física Nuclear da Universidade de Lisboa, Lisbon, Portugal; Departamento de Fisica, Universidade do Minho, Braga, Portugal; Departamento de Fisica Teorica y del Cosmos and CAFPE, Universidad de Granada, Granada, Spain; Dep Fisica and CEFITEC of Faculdade de Ciencias e Tecnologia, Universidade Nova de Lisboa, Caparica, Portugal; Institute of Physics, Academy of Sciences of the Czech Republic, Prague, Czech Republic; Czech Technical University in Prague, Prague, Czech Republic; Faculty of Mathematics and Physics, Charles University in Prague, Prague, Czech Republic; State Research Center Institute for High Energy Physics, Protvino, Russia; Particle Physics Department, Rutherford Appleton Laboratory, Didcot, UK; INFN Sezione di Roma, Rome, Italy; Dipartimento di Fisica, Sapienza Università di Roma, Rome, Italy; INFN Sezione di Roma Tor Vergata, Rome, Italy; Dipartimento di Fisica, Università di Roma Tor Vergata, Rome, Italy; INFN Sezione di Roma Tre, Rome, Italy; Dipartimento di Matematica e Fisica, Università Roma Tre, Rome, Italy; Faculté des Sciences Ain Chock, Réseau Universitaire de Physique des Hautes Energies, Université Hassan II, Casablanca, Morocco; Centre National de l’Energie des Sciences Techniques Nucleaires, Rabat, Morocco; Faculté des Sciences Semlalia, Université Cadi Ayyad, LPHEA-Marrakech, Marrakech, Morocco; Faculté des Sciences, Université Mohamed Premier and LPTPM, Oujda, Morocco; Faculté des Sciences, Université Mohammed V-Agdal, Rabat, Morocco; DSM/IRFU (Institut de Recherches sur les Lois Fondamentales de l’Univers), CEA Saclay (Commissariat à l’Energie Atomique et aux Energies Alternatives), Gif-sur-Yvette, France; Santa Cruz Institute for Particle Physics, University of California Santa Cruz, Santa Cruz, CA USA; Department of Physics, University of Washington, Seattle, WA USA; Department of Physics and Astronomy, University of Sheffield, Sheffield, UK; Department of Physics, Shinshu University, Nagano, Japan; Fachbereich Physik, Universität Siegen, Siegen, Germany; Department of Physics, Simon Fraser University, Burnaby, BC Canada; SLAC National Accelerator Laboratory, Stanford, CA USA; Faculty of Mathematics, Physics and Informatics, Comenius University, Bratislava, Slovak Republic; Department of Subnuclear Physics, Institute of Experimental Physics of the Slovak Academy of Sciences, Kosice, Slovak Republic; Department of Physics, University of Cape Town, Cape Town, South Africa; Department of Physics, University of Johannesburg, Johannesburg, South Africa; School of Physics, University of the Witwatersrand, Johannesburg, South Africa; Department of Physics, Stockholm University, Stockholm, Sweden; The Oskar Klein Centre, Stockholm, Sweden; Physics Department, Royal Institute of Technology, Stockholm, Sweden; Departments of Physics and Astronomy and Chemistry, Stony Brook University, Stony Brook, NY USA; Department of Physics and Astronomy, University of Sussex, Brighton, UK; School of Physics, University of Sydney, Sydney, Australia; Institute of Physics, Academia Sinica, Taipei, Taiwan; Department of Physics, Technion: Israel Institute of Technology, Haifa, Israel; Raymond and Beverly Sackler School of Physics and Astronomy, Tel Aviv University, Tel Aviv, Israel; Department of Physics, Aristotle University of Thessaloniki, Thessaloníki, Greece; International Center for Elementary Particle Physics and Department of Physics, The University of Tokyo, Tokyo, Japan; Graduate School of Science and Technology, Tokyo Metropolitan University, Tokyo, Japan; Department of Physics, Tokyo Institute of Technology, Tokyo, Japan; Department of Physics, University of Toronto, Toronto, ON Canada; TRIUMF, Vancouver, BC Canada; Department of Physics and Astronomy, York University, Toronto, ON Canada; Faculty of Pure and Applied Sciences, University of Tsukuba, Tsukuba, Japan; Department of Physics and Astronomy, Tufts University, Medford, MA USA; Centro de Investigaciones, Universidad Antonio Narino, Bogotá, Colombia; Department of Physics and Astronomy, University of California Irvine, Irvine, CA USA; INFN Gruppo Collegato di Udine, Sezione di Trieste, Udine, Italy; ICTP, Trieste, Italy; Dipartimento di Chimica Fisica e Ambiente, Università di Udine, Udine, Italy; Department of Physics, University of Illinois, Urbana, IL USA; Department of Physics and Astronomy, University of Uppsala, Uppsala, Sweden; Instituto de Física Corpuscular (IFIC) and Departamento de Física Atómica, Molecular y Nuclear and Departamento de Ingeniería Electrónica and Instituto de Microelectrónica de Barcelona (IMB-CNM), University of Valencia and CSIC, Valencia, Spain; Department of Physics, University of British Columbia, Vancouver, BC Canada; Department of Physics and Astronomy, University of Victoria, Victoria, BC Canada; Department of Physics, University of Warwick, Coventry, UK; Waseda University, Tokyo, Japan; Department of Particle Physics, The Weizmann Institute of Science, Rehovot, Israel; Department of Physics, University of Wisconsin, Madison, WI USA; Fakultät für Physik und Astronomie, Julius-Maximilians-Universität, Würzburg, Germany; Fachbereich C Physik, Bergische Universität Wuppertal, Wuppertal, Germany; Department of Physics, Yale University, New Haven, CT USA; Yerevan Physics Institute, Yerevan, Armenia; Centre de Calcul de l’Institut National de Physique Nucléaire et de Physique des Particules (IN2P3), Villeurbanne, France; CERN, 1211 Geneva 23, Switzerland

## Abstract

Combined analyses of the Higgs boson production and decay rates as well as its coupling strengths to vector bosons and fermions are presented. The combinations include the results of the analyses of the $H\rightarrow \gamma \gamma ,\, ZZ^*,\, WW^*,\, Z\gamma ,\, b\bar{b},\, \tau \tau$ and $\mu \mu$ decay modes, and the constraints on the associated production with a pair of top quarks and on the off-shell coupling strengths of the Higgs boson. The results are based on the LHC proton-proton collision datasets, with integrated luminosities of up to 4.7 $\mathrm {fb}^{-1}$ at $\sqrt{s}=7$ TeV and 20.3 $\mathrm {fb}^{-1}$ at $\sqrt{s}=8$ TeV, recorded by the ATLAS detector in 2011 and 2012. Combining all production modes and decay channels, the measured signal yield, normalised to the Standard Model expectation, is $1.18^{+0.15}_{-0.14}$. The observed Higgs boson production and decay rates are interpreted in a leading-order coupling framework, exploring a wide range of benchmark coupling models both with and without assumptions on the Higgs boson width and on the Standard Model particle content in loop processes. The data are found to be compatible with the Standard Model expectations for a Higgs boson at a mass of 125.36 GeV for all models considered.

## Introduction

In 2012, the ATLAS and CMS Collaborations at the Large Hadron Collider (LHC) reported the observation of a new particle at a mass of approximately 125 GeV [1, 2]. The discovery made in the search for the Standard Model (SM) Higgs boson (*H*), is a milestone in the quest to understand electroweak symmetry breaking (EWSB). Within the SM, EWSB is achieved through the Brout–Englert–Higgs mechanism [3–8] which predicts the existence of a neutral scalar particle, commonly known as the Higgs boson. While the SM does not predict the value of its mass ($m_H$), the production cross sections and decay branching ratios (BR) of the Higgs boson can be precisely calculated once the mass is known. Therefore, precision measurements of the properties of the new particle are critical in ascertaining whether the newly discovered particle is fully responsible for EWSB and whether there are potential deviations from SM predictions.

At the LHC, SM production of the Higgs boson is dominated by the gluon fusion process $gg\rightarrow H$ (ggF), followed by the vector-boson fusion process $qq'\rightarrow qq'H$ (VBF). Associated production with a *W* boson $q\bar{q}'\rightarrow WH$ (*WH*), a *Z* boson $q\bar{q}/gg\rightarrow ZH$ (*ZH*) or with a pair of top quarks $q\bar{q}/gg\rightarrow t\bar{t}H$ (*ttH*) have sizeable contributions as well. The *WH* and *ZH* production processes are collectively referred to as the *VH* process. Contributions are also expected from $b\bar{b}\rightarrow H$ (*bbH*) and production in association with a single top quark (*tH*). The latter proceeds through either the $qb\rightarrow tHq'$ or $gb\rightarrow WtH$ process. With the present dataset, the LHC is expected to be most sensitive to the Higgs boson decays of $H\rightarrow \gamma \gamma ,\, ZZ^*,\, WW^*,\, \tau \tau$ and $b\bar{b}$. Together they account for approximately 88 % of all decays of a SM Higgs boson at $m_H\sim 125$ GeV.

The discovery of the Higgs boson was made through analyses of the bosonic decay modes in $H\rightarrow \gamma \gamma$, $H\rightarrow ZZ^*\rightarrow 4\ell$ and $H\rightarrow WW^*\rightarrow \ell \nu \ell \nu$ ($\ell =e,\,\mu$) events. Since the discovery, these analyses have been improved and updated with more data [9–11]. The $H\rightarrow WW^*\rightarrow \ell \nu \ell \nu$ analysis has been supplemented with a dedicated *VH* analysis targeting $H\rightarrow WW^*$ [12]. The ATLAS Collaboration has measured the Higgs boson mass from the $H\rightarrow \gamma \gamma$ and $H\rightarrow ZZ^*\rightarrow 4\ell$ decays to be $m_H=125.36\pm 0.41$ GeV [13], reported results in the $H\rightarrow \tau \tau$ [14] and $H\rightarrow b\bar{b}$ [15] fermionic decay modes, and published upper limits on the rare decays $H\rightarrow Z\gamma$ [16] and $H\rightarrow \mu \mu$ [17]. Furthermore, constraints have been set on the *ttH* production rate [18–20] and on the off-shell coupling strengths of the Higgs boson [21]. These results are based on the full proton-proton collision data with integrated luminosities of up to 4.7 $\mathrm{fb}^{-1}$ at a centre-of-mass energy $\sqrt{s}=7$ TeV recorded in 2011 and 20.3 $\mathrm{fb}^{-1}$ at $\sqrt{s}=8$ TeV recorded in 2012 by the ATLAS detector at the LHC. A detailed description of the ATLAS detector can be found in Ref. [22].

This paper presents the combined results of the analyses mentioned above. These analyses are designed for maximum sensitivities to SM Higgs boson production from different processes, exploiting in particular the differences in kinematics through categorisation of the selected events. Thus the yields of different Higgs boson production processes and decays can be extracted. The Higgs boson coupling strengths to SM vector bosons and fermions in different benchmark models are probed for the measured Higgs boson mass of $m_H=125.36$ GeV. All results are obtained assuming the Higgs boson has a small total decay width such that its production and decay factorise. The ATLAS Collaboration has previously published combined studies of Higgs boson production and decay rates [23] and of spin-parity properties [24, 25] using diboson final states. The results are found to be consistent with expectations from the SM Higgs boson. Compared with the previous publication, the current results are based on the improved analysis sensitivities and the addition of information from other decay modes. A similar combination has been published by the CMS Collaboration [26].

The paper is organised as follows. Section 2 briefly summarises the individual analyses that are included in the combinations and Sect. 3 outlines the statistical method and the treatment of systematic uncertainties used in the combinations. In Sect. 4, the measured Higgs boson yields are compared with the SM predictions for different production processes and decay modes. In Sect. 5, the coupling strengths of the Higgs boson are tested through fits to the observed data. These studies probe possible deviations from the SM predictions under various assumptions, motivated in many cases by beyond-the-SM (BSM) physics scenarios. An upper limit on the branching ratio to invisible or undetected decay modes of the Higgs boson is also set. Finally, a brief summary is presented in Sect. 6.

## Input analyses to the combinations

The inputs to the combinations are the results from the analyses of $H\rightarrow \gamma \gamma ,\, ZZ^*,\, WW^*,\, \tau \tau ,\, b\bar{b},\, \mu \mu$ and $Z\gamma$ decay modes, and of the constraints on *ttH* and off-shell Higgs boson production. These analyses and changes made for the combinations are briefly discussed in this section. The ATLAS Collaboration has also performed a search for the rare $H\rightarrow J/\psi \gamma$ decay [27] which has the potential to constrain the Higgs boson coupling strength to the charm quark. However, the current result does not add sensitivity and is therefore omitted from the combinations. Furthermore, the inclusion of the results from direct searches for Higgs boson decays to invisible particles, such as those reported in Refs. [28, 29], is beyond the scope of the combinations presented in this paper.

The theoretical calculations of the Higgs boson production cross sections and decay branching ratios have been compiled in Refs. [30–32] and are summarised in Table 1. For the ggF process, the cross section is computed at up to NNLO in QCD corrections [33–38] and NLO in electroweak (EW) corrections [39–41]. The effects of QCD soft-gluon resummations at up to NNLL [42] are also applied. These calculations are described in Refs. [43–47]. For the VBF process, full QCD and EW corrections up to NLO [48–50] and approximate NNLO [51, 52] QCD corrections are used to calculate the cross section. The cross sections of the *WH* and *ZH* ($q\bar{q}\rightarrow ZH$) are calculated including QCD corrections up to NNLO [53, 54] and EW corrections up to NLO [55, 56] whereas the cross section of the $gg\rightarrow ZH$ process is calculated up to NLO in QCD corrections [57, 58]. The cross section for *ttH* is computed up to NLO in QCD [59–62]. For the *bbH* process, the cross section is calculated in QCD corrections up to NLO [63–65] in the four-flavour scheme and up to NNLO [66] in the five-flavour scheme with the Santander matching scheme [67]. The cross sections of the *tH* processes used are calculated at up to NLO in QCD corrections [68, 69]. The PDF sets used in these calculations are CT10 [70, 71], MSTW2008 [72], NNPDF2.1 [73, 74] and NNPDF2.3 [75] following the prescription of Ref. [76]. The decay branching ratios of the Higgs boson are calculated using the Hdecay [77, 78] and Prophecy4f [79, 80] programs, compiled in Ref. [81].

**Table 1:** SM predictions of the Higgs boson production cross sections and decay branching ratios and their uncertainties for $m_H=125.36$ GeV, obtained by linear interpolations from those at 125.3 and 125.4 GeV from Ref. [32] except for the *tH* production cross section which is obtained from Refs. [20, 82]. The uncertainties of the cross sections are the sum in quadrature of the uncertainties resulting from variations of QCD scales, parton distribution functions and $\alpha _\mathrm{s}$. The uncertainty on the *tH* cross section is calculated following the procedure in Refs. [20, 32]

Production process	Cross section (pb)		Decay channel	Branching ratio (%)
	$\sqrt{s}=7$ TeV	$\sqrt{s}=8$ TeV		
ggF	$15.0\pm 1.6$	$19.2\pm 2.0$	$H\rightarrow b\bar{b}$	$57.1\pm 1.9$
VBF	$1.22\pm 0.03$	$1.57\pm 0.04$	$H\rightarrow WW^*$	$22.0\pm 0.9$
*WH*	$0.573\pm 0.016$	$0.698\pm 0.018$	$H\rightarrow gg$	$8.53\pm 0.85$
*ZH*	$0.332\pm 0.013$	$0.412\pm 0.013$	$H\rightarrow \tau \tau$	$6.26\pm 0.35$
*bbH*	$0.155\pm 0.021$	$0.202\pm 0.028$	$H\rightarrow c\bar{c}$	$2.88\pm 0.35$
*ttH*	$0.086\pm 0.009$	$0.128\pm 0.014$	$H\rightarrow ZZ^*$	$2.73\pm 0.11$
*tH*	$0.012\pm 0.001$	$0.018\pm 0.001$	$H\rightarrow \gamma \gamma$	$0.228\pm 0.011$
Total	$17.4 \pm 1.6$	$22.3\pm 2.0$	$H\rightarrow Z\gamma$	$0.157\pm 0.014$
			$H\rightarrow \mu \mu$	$0.022\pm 0.001$

**Table 2:** Summary of event generators, showering programs and PDF sets used to model the Higgs boson production and decays at $\sqrt{s}=8$ TeV

Production process	Event generator	Showering program	PDF set
ggF	Powheg	Pythia6/Pythia8	CT10
VBF	Powheg	Pythia6/Pythia8	CT10
*WH*	Pythia8	Pythia8	CTEQ6L1
$ZH:\ q\bar{q}\rightarrow ZH$	Pythia8	Pythia8	CTEQ6L1
$ZH:\ gg\rightarrow ZH$	Powheg	Pythia8	CT10
*ttH*	Powheg	Pythia8	CT10
*bbH*	MadGraph5_aMC@NLO	Herwig++	CT10
$tH:\ qb\rightarrow tHq'$	MadGraph	Pythia8	CT10
$tH:\ gb\rightarrow WtH$	MadGraph5_aMC@NLO	Herwig++	CT10

All analyses use Monte Carlo (MC) samples to model the acceptances of the Higgs boson events. Table 2 summarises the event generators and parton distribution functions (PDF) used for the analyses of the $\sqrt{s}=8$ TeV data. The modelling at $\sqrt{s}=7$ TeV is similar, with one notable difference of Pythia6 [83] replacing Pythia8 [84]. The ggF and VBF production of the Higgs boson are simulated with the next-to-leading order (NLO) matrix-element Powheg program [85–89] interfaced to either Pythia6 or Pythia8 for the simulation of the underlying event, parton showering and hadronisation (referred to as the showering program). The Higgs boson transverse momentum distribution from ggF production is reweighted to match the calculation of HRes2.1 [90, 91], which includes QCD corrections up to the next-to-next-to-leading order (NNLO) and next-to-next-to-leading logarithm (NNLL) in perturbative expansions. Furthermore, ggF events with two or more jets are reweighted to match the transverse momentum distribution from MiNLO HJJ predictions [92]. The *WH* and *ZH* ($q\bar{q}\rightarrow ZH$) production processes are simulated with the leading-order (LO) Pythia8 program. The $gg\rightarrow ZH$ process contributes approximately 8 % to the total *ZH* production cross section in the SM. For most of the analyses, the process is modelled using $q\bar{q}\rightarrow ZH$ of Pythia8. Only the *VH* analysis in the $H\rightarrow b\bar{b}$ decay mode specifically models $gg\rightarrow ZH$ production using Powheg [85–87] interfaced to Pythia8. The *ttH* process is modelled using the NLO calculation in the HELAC-Oneloop package [93] interfaced to Powheg and Pythia8 for the subsequent simulation. The *tH* production process is simulated using MadGraph [94] interfaced to Pythia8 for $qb\rightarrow tHq'$ and using MadGraph5_aMC@NLO [82] interfaced to Herwig++ [95] for $gb\rightarrow WtH$. The *bbH* production process contributes approximately 1 % [96] to the total Higgs boson cross section in the SM. It is simulated with the MadGraph5_aMC@NLO program for some analyses. The event kinematics of ggF and *bbH* production are found to be similar for analysis categories that are most important for *bbH*. Thus the acceptance times efficiency for *bbH* is assumed to be the same as for ggF for all analyses. The PDF sets used in the event generations are CT10 [70] and CTEQ6L1 [97]. All Higgs boson decays are simulated by the showering programs.

Throughout this paper, the signal-strength parameter $\mu$ is defined as the ratio of the measured Higgs boson yield to its SM expectation:

1 $$\begin{aligned} \mu = \frac{\sigma \times \mathrm{BR}}{(\sigma \times \mathrm{BR})_\mathrm{SM}}\,. \end{aligned}$$

Here $\sigma$ is the production cross section of the Higgs boson. For a specific production process *i* and decay channel *f*, i.e., $i\rightarrow H\rightarrow f$, the signal-strength parameter is labelled as $\mu _i^f$ and can be factorised in terms of the signal strengths of production ($\mu _i$) and decay ($\mu _f$):

2 $$\begin{aligned}&\mu _i^f = \frac{\sigma _i\times \mathrm{BR}_f}{(\sigma _i\times \mathrm{BR}_f)_\mathrm{SM}}\equiv \mu _i\times \mu _f, \nonumber \\&\quad \text {with } \ \mu _i=\frac{\sigma _i}{(\sigma _i)_\mathrm{SM}}\quad \text {and} \quad \mu _f= \frac{\mathrm{BR}_f}{(\mathrm{BR}_f)_\mathrm{SM}}. \end{aligned}$$

Thus for each analysis category (*c*) as discussed later in this section, the number of signal events ($n^c_s$) can be written as:

3 $$\begin{aligned} n^c_s =\sum _i \sum _f \mu _i (\sigma _i)_\mathrm{SM} \times \mu _f (\mathrm{BR}_f)_\mathrm{SM} \times A^c_{if} \times \varepsilon ^c_{if} \times \mathcal {L}^c \end{aligned}$$

where the indices *i* and *f* indicate the production processes and decays contributing to the category, $A^c_{if}$ represents the detector acceptance derived from simulation of the SM process, $\varepsilon ^c_{if}$ is the reconstruction efficiency within the acceptance and $\mathcal {L}^c$ the integrated luminosity for the given category *c* of the given channel.

However, the experimental data do not allow to separately determine $\mu _i$ and $\mu _f$ for any given process since only their product is measured. All combined fits of signal strengths presented in this paper make assumptions about the relationship between $\mu _i$ of different production processes or similarly between $\mu _f$ of different decay modes. Thus the meaning of the signal strength depends on the assumptions made. Nevertheless, the production and decays can be factorised using the ratios of cross sections and of branching ratios as discussed in Sect. 4.4.

Leptons ($\ell$) refer to electrons or muons unless specified otherwise; the symbols $\tau _{\mathrm { lep}}$ and $\tau _{\mathrm { had}}$ refer to $\tau$ leptons identified through their decays to leptons or hadrons; and variables $p_{\text {T}}$, $E_{\text {T}}$ and $E_{\text {T}}^{\text {miss}}$ refer to transverse momentum, transverse energy and missing transverse momentum, respectively. Notations indicating particle charges or antiparticles are generally omitted.

The ATLAS experiment uses a right-handed coordinate system with its origin at the nominal interaction point (IP) in the centre of the detector and the *z*-axis along the beam pipe. The *x*-axis points from the IP to the centre of the LHC ring, and the *y*-axis points upward. Cylindrical coordinates $(r,\phi )$ are used in the transverse plane, $\phi$ being the azimuthal angle around the beam pipe. The pseudorapidity is defined in terms of the polar angle $\theta$ as $\eta =-\ln \tan (\theta /2)$.

**Fig. 1:**
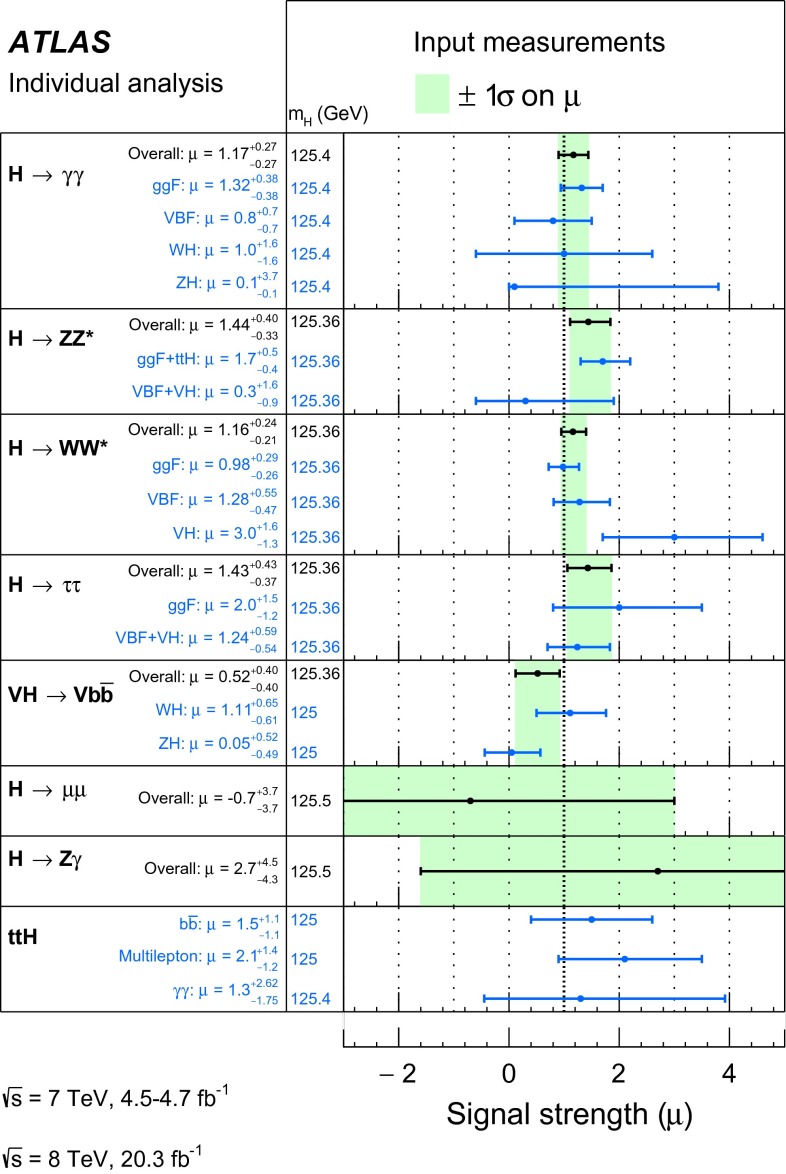
Summary of the signal-strength measurements, as published, from individual analyses that are inputs to the combinations. The Higgs boson mass column indicates the $m_H$ value at which the result is quoted. The overall signal strength of each analysis (*black*) is the combined result of the measurements for different production processes (*blue*) assuming SM values for their cross-section ratios. The *error bars* represent $\pm 1\sigma$ total uncertainties, combining statistical and systematic contributions. The *green shaded bands* indicate the uncertainty on the overall signal strength obtained by each analysis. The combined signal strength of the $H\rightarrow \gamma \gamma$ analysis also includes the *ttH* contribution which is listed separately under *ttH* production

Table 3 gives an overview of the analyses that are inputs to the combinations and their main results, as published. An essential feature of these analyses is the extensive application of exclusive categorisation, i.e., classifying candidate events based on the expected kinematics of the different Higgs boson production processes. The categorisation not only improves the analysis sensitivity, but also allows for the discrimination among different production processes. Figure 1 summarises the signal-strength measurements of different production processes that are used as inputs to the combinations.

**Table 3:** Overview of the individual analyses that are included in the combinations described in this paper. The signal strengths, the statistical significances of a Higgs boson signal, or the 95 % CL upper limits on the Higgs boson production rates or properties are also shown wherever appropriate. A range is quoted for the upper limit on the off-shell signal strength, depending on the assumption for the continuum $gg\rightarrow WW/ZZ$ cross section. These results are taken directly from the individual publications. Results of the on-shell analyses are quoted for $m_H=125.36$ GeV except that $m_H=125.5$ GeV is assumed for the $H\rightarrow Z\gamma$ and $H\rightarrow \mu \mu$ analyses and that $m_H=125$ GeV is used for the *ttH* searches with $H\rightarrow b\bar{b}$ and $ttH\rightarrow \mathrm{multileptons}$. The luminosity used for the $\sqrt{s}=7$ TeV $VH(\rightarrow b\bar{b})$ analysis differs slightly from the values used for other analyses because a previous version of the luminosity calibration was applied. The significance is given in units of standard deviations (SD). The numbers in parentheses are the expected values for the SM Higgs boson. The *ttH* analysis in the $H\rightarrow \gamma \gamma$ decay is part of the $H\rightarrow \gamma \gamma$ analysis. It is included separately under the *ttH* production for completeness. The checkmark ($\checkmark$) indicates whether the analysis is performed for the respective $\sqrt{s}=7$ and 8 TeV dataset

Analysis	Signal		$\int \mathcal{L} dt \ \mathrm{fb}^{-1}$	
Categorisation or final states	Strength $\mu$	Significance [SD]	7 TeV	8 TeV
$H\rightarrow \gamma \gamma$ [9]	$1.17\pm 0.27$	5.2 (4.6)	4.5	20.3
*ttH*: leptonic, hadronic			$\checkmark$	$\checkmark$
*VH*: one-lepton, dilepton, $E_{\text {T}}^{\text {miss}}$, hadronic			$\checkmark$	$\checkmark$
VBF: tight, loose			$\checkmark$	$\checkmark$
ggF: 4 $p_{\mathrm {Tt}}$ categories			$\checkmark$	$\checkmark$
$H\rightarrow ZZ^*\rightarrow 4\ell$ [10]	$1.44^{+0.40}_{-0.33}$	8.1 (6.2)	4.5	20.3
VBF			$\checkmark$	$\checkmark$
*VH*: hadronic, leptonic			$\checkmark$	$\checkmark$
ggF			$\checkmark$	$\checkmark$
$H\rightarrow WW^*$ [11, 12]	$1.16^{+0.24}_{-0.21}$	6.5 (5.9)	4.5	20.3
ggF: (0-jet, 1-jet) $\otimes$ ($ee+\mu \mu ,\ e\mu$)			$\checkmark$	$\checkmark$
ggF: $\ge 2$-jet and $e\mu$				$\checkmark$
VBF: $\ge 2$-jet $\otimes$ ($ee+\mu \mu ,\ e\mu$)			$\checkmark$	$\checkmark$
*VH*: opposite-charge dilepton, three-lepton, four-lepton			$\checkmark$	$\checkmark$
*VH*: same-charge dilepton				$\checkmark$
$H\rightarrow \tau \tau$ [14]	$1.43^{+0.43}_{-0.37}$	4.5 (3.4)	4.5	20.3
Boosted: $\tau _\mathrm{lep}\tau _\mathrm{lep}, \tau _\mathrm{lep}\tau _\mathrm{had}, \tau _\mathrm{had}\tau _\mathrm{had}$			$\checkmark$	$\checkmark$
VBF: $\tau _\mathrm{lep}\tau _\mathrm{lep}, \tau _\mathrm{lep}\tau _\mathrm{had}, \tau _\mathrm{had}\tau _\mathrm{had}$			$\checkmark$	$\checkmark$
$VH\rightarrow Vb\bar{b}$ [15]	$0.52\pm 0.40$	1.4 (2.6)	4.7	20.3
$0\ell \ (ZH\rightarrow \nu \nu b\bar{b})$: $N_\mathrm{jet}=2,3$, $N_\mathrm{btag}=1,2$, $p_{\text {T}} ^V\in$ 100–120 and $>\!120$ GeV			$\checkmark$	$\checkmark$
$1\ell \ (WH\rightarrow \ell \nu b\bar{b})$: $N_\mathrm{jet}=2,3$, $N_\mathrm{btag}=1,2$, $p_{\text {T}} ^V<$ and $>\!120$ GeV			$\checkmark$	$\checkmark$
$2\ell \ (ZH\rightarrow \ell \ell b\bar{b})$: $N_\mathrm{jet}=2,3$, $N_\mathrm{btag}=1,2$, $p_{\text {T}} ^V<$ and $>\!120$ GeV			$\checkmark$	$\checkmark$

	95 % CL limit			
$H\rightarrow Z\gamma$ [16]	$\mu <11\ (9)$		4.5	20.3
10 categories based on $\Delta \eta _{Z\gamma }$ and $p_{\mathrm {Tt}}$			$\checkmark$	$\checkmark$
$H\rightarrow \mu \mu$ [17]	$\mu <7.0\ (7.2)$		4.5	20.3
VBF and 6 other categories based on $\eta _\mu$ and $p_{\text {T}} ^{\mu \mu }$			$\checkmark$	$\checkmark$
*ttH* production [18–20]			4.5	20.3
$H\rightarrow b\bar{b}$: single-lepton, dilepton	$\mu <3.4\ (2.2)$			$\checkmark$
$ttH\rightarrow$multileptons: categories on lepton multiplicity	$\mu <4.7\ (2.4)$			$\checkmark$
$H\rightarrow \gamma \gamma$: leptonic, hadronic	$\mu <6.7\ (4.9)$		$\checkmark$	$\checkmark$
Off-shell $H^*$ production [21]	$\mu <$ 5.1–8.6 (6.7–11.0)			20.3
$H^*\rightarrow ZZ\rightarrow 4\ell$				$\checkmark$
$H^*\rightarrow ZZ\rightarrow 2\ell 2\nu$				$\checkmark$
$H^*\rightarrow WW\rightarrow e\nu \mu \nu$				$\checkmark$

### $H\rightarrow \gamma \gamma$

In the $H\rightarrow \gamma \gamma$ analysis, described in detail in Ref. [9], the Higgs boson signal is measured in events with at least two isolated and well-identified photon candidates. The leading and subleading photon candidates are required to have $E_{\text {T}}/m_{\gamma \gamma } > 0.35$ and 0.25, respectively, where $m_{\gamma \gamma }$ is the invariant mass of the two selected photons. The diphoton candidate events are grouped into twelve exclusive categories separately for the $\sqrt{s}=7$ and 8 TeV datasets; the order of categorisation is chosen to give precedence to production modes with the most distinct signatures. Each category is optimised by adjusting the event selection criteria to minimise the expected uncertainty on the signal yield of the targeted production mode.

The first two categories are designed for *ttH* production based on the topology of leptonic and hadronic decays of the associated $t\bar{t}$ pair. They are described in Sect. 2.8. The next four categories are optimised for *VH* production, targeting one-lepton, dilepton, $E_{\text {T}}^{\text {miss}}$, and hadronic signatures of *W* and *Z* boson decays. Events from VBF production are identified by requiring two well-separated and high-$p_{\text {T}}$ jets and little hadronic activity between them. A boosted decision tree (BDT) [98, 99] algorithm is employed to maximise the VBF signal and background separation. Events are sorted into two categories with different VBF purities according to the output value of the BDT. Finally, the remaining events are separated into four categories based on the pseudorapidities of the photons and the $p_{\mathrm {Tt}}$ of the diphoton system [9], the diphoton momentum transverse to its thrust axis in the transverse plane.

For most of the categories, the background is composed of a mixture of $\gamma$–jet and jet–jet events, where one or two jets are misidentified as photons, and $\gamma \gamma$ events. In particular the $\gamma \gamma$ background is dominant and irreducible. The Higgs boson signal is extracted from maximum-likelihood fits of a narrow resonance plus continuum background models to unbinned diphoton invariant-mass distributions observed in the different event categories. In the fit, the signal is modelled by the sum of a Crystal Ball function [100] and a smaller but wider Gaussian component while the backgrounds are modelled by category-dependent exponential functions of first- or second-order polynomials.

### $H\rightarrow ZZ^*\rightarrow 4\ell$

The $H\rightarrow ZZ^*\rightarrow 4\ell$ analysis, described in detail in Ref. [10], has a high signal-to-background ratio, which is about two for each of the four final states considered: $4\mu$, $2e2\mu$, $2\mu 2e$, and 4*e*, where the first lepton pair has an invariant mass closer to the *Z* boson mass. The analysis selects Higgs boson candidates by requiring two pairs of isolated, same-flavour and opposite-charge leptons with one of the two pairs having a dilepton invariant mass in the range 50 – 106 GeV.

To measure the rates of different production processes, each $H\rightarrow ZZ^*\rightarrow 4\ell$ candidate is assigned to one of four categories depending on event characteristics beyond the four selected leptons. The VBF category consists of candidates with two additional jets with dijet mass $m_{jj}>130$ GeV. The events failing this selection are considered for the *VH*-hadronic category, where the dijet mass is required to be $40\,\mathrm{GeV} <m_{jj}<130\,\mathrm{GeV}$. Events failing the VBF and *VH*-hadronic categorisation criteria are considered for the *VH*-leptonic category with the requirement of an additional lepton. Finally, the remaining events are assigned to the ggF category. The separation of VBF and *VH* production from the dominant ggF production mode is improved by exploiting two BDT discriminants trained on the jet kinematics, one for the VBF category and the other for the *VH*-hadronic category. A third BDT discriminant based on the four-lepton kinematics is used to improve the separation between the ggF signal and its main background.

The largest background comes from continuum $ZZ^*$ production and is estimated using simulation normalised to the SM next-to-leading-order cross-section calculation. For the four-lepton events with an invariant mass, $m_{4\ell }$, below about 160 GeV, there are also important background contributions from *Z*+jets and $t\bar{t}$ production with two prompt leptons, where the additional charged lepton candidates arise from decays of hadrons with *b*- or *c*-quark content, from photon conversions or from misidentified jets. Their contributions are estimated with data-driven methods.

For each category, the signal is extracted from a maximum-likelihood fit to either the $m_{4\ell }$ distribution (*VH* categories) or the combined two-dimensional distributions of $m_{4\ell }$ and a BDT discriminant (ggF and VBF categories). The four-lepton mass range of $110\,\mathrm{GeV}<m_{4\ell }<140\,\mathrm{GeV}$ is included in the fits.

### $H\rightarrow WW^*$

Analyses targeting the ggF, VBF, and *VH* production modes [11, 12] are performed for the $H\rightarrow WW^*$ decay channel. The ggF and VBF production processes are explored through the $H\rightarrow WW^*\rightarrow \ell \nu \ell \nu$ decay and the *VH* process is studied in final states with two or more leptons.

The analysis of the ggF and VBF production processes [11] selects the signal candidate events by requiring two oppositely charged leptons. Candidates are categorised according to the number of jets ($N_\mathrm{jet}$) and to the flavours of the leptons. The $N_\mathrm{jet}$ categorisation separates the large top-quark production background from the ggF signal while the categorisation by lepton flavours isolates the challenging Drell–Yan background in the same-flavour categories. The categories targeting ggF production include $N_\mathrm{jet}=0,\, 1$ and $\ge$2 and are further divided into the same- and different-flavour leptons for $N_\mathrm{jet}=0,\, 1$. Only the different-flavour leptons are considered for $N_\mathrm{jet}\ge 2$. The categories targeting VBF production require $N_\mathrm{jet}\ge 2$, separately for the same- or different-flavour leptons. The primary background processes are *WW*, top quark ($t\bar{t}$ and *Wt*), *W*+jets, Drell–Yan, and other diboson (*WZ*, $W\gamma$, $W\gamma ^{*}$, and *ZZ*) production. Most of the background contributions are estimated using data. For the ggF categories, the final signal region is selected by requiring the dilepton mass $m_{\ell \ell } < 55$ GeV and their azimuthal angular separation $\Delta \phi _{\ell \ell } < 1.8$ and the signal is extracted through a combined fit to the transverse mass distributions of the dilepton plus $E_{\text {T}}^{\text {miss}}$ system in both the signal and control regions of different categories and lepton flavours. For the VBF categories, a BDT combining information such as rapidity separation and mass of the two leading jets and the dilepton angular separation, is used as the final discriminant, from which the signal is extracted.

The *VH* analysis [12] is optimised for different lepton multiplicities: opposite-charge dileptons, same-charge dileptons, three and four leptons. Most final states are required to have $E_{\text {T}}^{\text {miss}}$ and events with a *b*-tagged jet are vetoed. Dilepton final states target *VH* production with the $H\rightarrow WW^*$ decay with two bosons decaying leptonically and the other hadronically. The opposite-charge dilepton final state selects events with two or more jets, with the value of $m_{jj}$ required to be close to the *W* and *Z* boson masses. Similar to the ggF $N_\mathrm{jet}\ge 2$ category, the dominant background is from top quark production. The same-charge dilepton category accepts events with either one or two jets. The dominant backgrounds are from *WZ*, $W\gamma ^{(*)}$, and *W*+jets production. The three-lepton final state targets *WH* with $H\rightarrow WW^*$ and has the highest sensitivity of the four final states. The three leptons are required to have a net charge of $\pm 1$ and the event can have at most one jet. The dominant background process is *WZ* production and is reduced with a $Z\rightarrow \ell \ell$ veto. The four-lepton category is designed to accept events from *ZH* production with the $H\rightarrow WW^*$ decay. The net charge of the leptons is required to be zero and at least one pair of leptons is required to have the same flavour, opposite charge, and an invariant mass close to the *Z* boson mass. The dominant background is SM $ZZ^*$ production. In the three-lepton category, the signal yield is extracted through fits to distributions of a BDT or the minimum separation in the $\eta -\phi$ plane between opposite-charge leptons depending on the lepton flavours. For other categories, the event yields are used, without exploiting information on the shapes of distributions.

### $H\rightarrow \tau \tau$

The $H\rightarrow \tau \tau$ analysis [14] considers both the leptonic ($\tau _{\mathrm { lep}}$) and hadronic ($\tau _{\mathrm { had}}$) decays of the $\tau$ lepton. Three sub-channels ($\tau _\mathrm{lep}\tau _\mathrm{lep}$, $\tau _\mathrm{lep}\tau _\mathrm{had}$ and $\tau _\mathrm{had}\tau _\mathrm{had}$) are defined by orthogonal requirements on the number of reconstructed hadronic $\tau$ decays and leptons (electrons or muons) in the event.^1^

Candidate events are divided into boosted and VBF categories. The boosted category targets signal events where the Higgs boson is produced with a large boost, primarily from the gluon fusion process, and requires the transverse momentum of the reconstructed Higgs boson candidate to be greater than 100 GeV. The VBF category contains events with two jets separated in pseudorapidity and targets signal events produced through the vector boson fusion process. A separate BDT is then employed in each category and sub-channel to discriminate signal from background, utilising between five and nine input variables, chosen in order to exploit discriminating features such as Higgs boson decay properties, event activity, and the VBF topology in the corresponding category. One of the most important input variables is the mass of the $\tau \tau$ system, which is quite challenging to reconstruct due to the presence of at least two neutrinos in the final state; the Missing Mass Calculator [101] is used for this purpose.

In all three sub-channels, the most important backgrounds are irreducible $Z\rightarrow \tau \tau$ events, and events with one or two jets misidentified as $\tau$ lepton decay products (primarily from multijet and *W*+jets production). To estimate the $Z\rightarrow \tau \tau$ background the embedding technique [102] is used, where $Z\rightarrow \mu \mu$ events are selected in data and the reconstructed muons are replaced by simulated $\tau$ lepton decays. Fully data-driven techniques are used for the estimation of backgrounds from misidentified $\tau$ decay products, while Monte Carlo simulation corrected to data is used for other backgrounds, such as the top quark and $Z\rightarrow \ell \ell$ production.

The signal is extracted by fitting the shape of the BDT discriminant with signal and background templates simultaneously in all signal regions. The fit also includes dedicated control regions enriched with top quark, $Z\rightarrow \ell \ell$ and multijet events. These control regions are used to constrain normalisations of the corresponding backgrounds.

### *VH* with $H\rightarrow b\bar{b}$

The $H\rightarrow b\bar{b}$ decay mode is predicted in the SM to have the largest branching ratio (see Table 1). In spite of this large branching ratio, an inclusive search for $H\rightarrow b\bar{b}$ is not feasible because of the overwhelming background from multijet production. Associated production of a Higgs boson with a vector boson *V* (*W* or *Z*), offers a viable alternative because leptonic decays of the vector boson, $W\rightarrow \ell \nu$, $Z\rightarrow \ell \ell$, and $Z\rightarrow \nu \nu$, can be efficiently used for triggering and background reduction.

The search for associated *VH* production with $H\rightarrow b\bar{b}$ [15] is performed for events containing zero, one, or two charged leptons. Contributions from $W\rightarrow \tau \nu$ and $Z\rightarrow \tau \tau$ decays in which the $\tau$ leptons subsequently decay to electrons or muons are also included. A *b*-tagging algorithm is used to identify jets from $H\rightarrow b\bar{b}$ decays. To improve the sensitivity, the three channels are each split into categories according to the vector-boson transverse momentum, $p_{\text {T}} ^V$, the number of jets, and the number and quality of the *b*-tagged jets. Topological and kinematic selection criteria are applied within each of the resulting categories. The categories providing most of the sensitivity are those requiring two *b*-tagged jets and large $p_{\text {T}} ^V$. The categories with low sensitivity are used to constrain the contributions of the dominant background processes.

A binned profile maximum-likelihood fit to all categories simultaneously is used to extract the signal yield and the background normalisations. The most significant background sources are *V*+heavy-flavour-jet production and $t\bar{t}$ production. The normalisations of these backgrounds are fully determined by the likelihood fit. Other significant background sources are single-top-quark and diboson (*WZ* and *ZZ*) production, with normalisations from theory, as well as multijet events. The shapes of all backgrounds are estimated from simulation, except for the multijet background for which the shape and normalisation are obtained using multijet-enriched control samples.

Two versions of the analysis are performed. In the dijet-mass analysis, the mass of the dijet system of *b*-tagged jets is the final discriminating variable used in the statistical analysis. In the multivariate analysis (MVA), which incorporates various kinematic variables in addition to the dijet mass and the *b*-tagging information, the outputs of boosted decision trees provide the final discriminating variable. Since the MVA has higher expected sensitivity, it is chosen as the nominal analysis for the $\sqrt{s}=8$ TeV dataset to extract the final results. For the $\sqrt{s}=7$ TeV dataset, only a dijet-mass analysis is performed.

The $\sqrt{s}=7$ TeV $VH(\rightarrow b\bar{b})$ analysis uses a previous version of the luminosity calibration and therefore has a slightly different luminosity value compared with those quoted for other analyses. However, this small difference is expected to have negligible effects on the results presented in this paper.

### $H\rightarrow Z\gamma$

The $H\rightarrow Z\gamma$ analysis [16] with $Z\rightarrow \ell \ell$ searches for a narrow peak in the reconstructed $\ell \ell \gamma$ invariant-mass distribution around 125 GeV over a smooth background. The $Z{+}\gamma$ production, $Z\rightarrow \ell \ell \gamma$ radiative decays and *Z*+jets events where a jet is misidentified as a photon dominate the background contributions.

The analysis selects two isolated leptons of same flavour and opposite charge and one isolated photon. Due to the kinematics of the decay, low $p_{\text {T}}$ thresholds are applied to the leptons and the photon. The invariant mass of the dilepton system must satisfy $m_{\ell \ell }>m_Z-10$ GeV and the three-body invariant mass must be consistent with the mass of the Higgs boson. To enhance the sensitivity of the analysis, events are classified into categories with different signal-to-background ratios and invariant-mass resolutions, based on the pseudorapidity difference $\Delta \eta _{Z\gamma }$ between the photon and the *Z* boson and $p_\mathrm{Tt}$, the component of the Higgs boson candidate $p_{\text {T}}$ that is orthogonal to the $Z\gamma$ thrust axis in the transverse plane.

The final discrimination between signal and background events is based on a simultaneous likelihood fit to the $m_{\ell \ell \gamma }$ spectra in each category, separately for the $\sqrt{s}=7$ and 8 TeV datasets. Similar to the $H\rightarrow \gamma \gamma$ analysis (Sect. 2.1), the signal is modelled with the sum of a Crystal Ball function and a smaller but wider Gaussian component while the backgrounds are modelled with polynomials, or exponentiated polynomials depending on categories.

### $H\rightarrow \mu \mu$

The $H\rightarrow \mu \mu$ analysis [17] searches for a narrow peak in the dimuon invariant mass $m_{\mu \mu }$ distribution over a smooth background, where the width of the signal is dominated by the experimental resolution. The mass spectrum is dominated by the continuously falling background due to $Z/\gamma ^*$ production, with smaller contributions from top quark and diboson production.

The selected events containing a pair of oppositely charged muons are separated into seven mutually exclusive categories based on the VBF dijet signature, the muon pseudorapidity $\eta _\mu$, and the transverse momentum of the dimuon system $p_\mathrm{T}^{\mu \mu }$. The events with two or more jets that match selections designed for the VBF process are accepted in the VBF signal region. All other selected events are split up into six categories based on the values of $\eta _{\mu }$ and $p_\mathrm{T}^{\mu \mu }$. This categorisation takes advantage of the higher momentum resolution for muons reconstructed in the central part of the detector, and high $p_\mathrm{T}^{\mu \mu }$ for the expected SM signal.

The $m_{\mu \mu }$ distribution in the 110–160 GeV region is fitted with an analytic signal-plus-background model separately for the $\sqrt{s}=7$ and 8 TeV datasets, setting a limit on the dimuon decay of the SM Higgs boson with a mass of 125.5 GeV. In the fit, the signal is modelled as the sum of a Crystal Ball function and a Gaussian function in all regions while the backgrounds are modelled using exponentials or polynomials.

### *ttH* production

Searches for $q\bar{q}/gg\rightarrow t\bar{t}H$ production have been performed with three analyses targeting the Higgs boson decays $H\rightarrow b\bar{b}$, $H\rightarrow (WW^*,\,\tau \tau ,\,ZZ^*)\rightarrow \mathrm{leptons}$, and $H\rightarrow \gamma \gamma$. The search in the $H\rightarrow \gamma \gamma$ decay mode uses both $\sqrt{s}=7$ and 8 TeV data, while the other two use only the $\sqrt{s}=8$ TeV data.

The search for *ttH* production with $H\rightarrow b\bar{b}$ [18] considers two separate selections optimised for single-lepton and dilepton final states of $t\bar{t}$ decays. In the single-lepton channel, events are required to have one isolated electron or muon and at least four jets. In the dilepton channel, events are required to have two opposite-charge leptons (*ee*, $\mu \mu$ or $e\mu$) and at least two jets; events consistent with originating from a $Z\rightarrow \ell \ell$ decay are rejected. In both cases at least two *b*-tagged jets are required. Candidate events are categorised according to the jet and *b*-jet multiplicities with a total of nine (six) categories for the single-lepton (dilepton) final states. The background is dominated by $t\bar{t}$+jets events, with increasing fractions of $t\bar{t}b\bar{b}$ and $t\bar{t}c\bar{c}$ at the higher *b*-jet multiplicities characteristic of signal events. The analysis uses a neural network to discriminate signal from background in the most signal-like categories. Simpler kinematic discriminants are used in background-like categories.

The *ttH* search with $H\rightarrow WW^*,\ \tau \tau$ and $ZZ^*$ decays [19] exploits several multilepton signatures resulting from leptonic decays of vector bosons and/or the presence of $\tau$ leptons. The events are categorised by the number of reconstructed electrons or muons and hadronic $\tau$ candidates. The five channels used in this combination are: one lepton with two hadronic $\tau$ candidates, two same-charge leptons with zero or one hadronic $\tau$ candidate, three leptons, and four leptons. The largest backgrounds in the analysis are non-prompt leptons, primarily arising from semileptonic *b*-hadron decays in $t\bar{t}$ events; electron charge misreconstruction in events where opposite-charge leptons are produced; and the production of $t\bar{t} W$ and $t\bar{t} Z$ ($t\bar{t} V$). The potential signal is determined from the numbers of observed events in data and of the estimated number of background events.

The *ttH* search in the $H\rightarrow \gamma \gamma$ channel [20] is part of the $H\rightarrow \gamma \gamma$ analysis (see Sect. 2.1) and employs the same diphoton selection. The leptonic as well as fully hadronic decay signatures of the $t\bar{t}$ system are considered. The leptonic selection requires at least one lepton and one *b*-tagged jet as well as $E_{\text {T}}^{\text {miss}}$. In the hadronic selection, different combinations of jet and *b*-tagging multiplicities are applied to improve the signal sensitivity. The small contribution from ggF, VBF and *VH* production is estimated from Monte Carlo simulation. The *ttH* signal is extracted from a fit to the observed diphoton mass distribution.

### Off-shell Higgs boson production

Measurements of the $H^*\rightarrow ZZ$ and $H^*\rightarrow WW$ final states in the mass range above the $2m_Z$ and $2m_W$ thresholds (off-shell region) provide a unique opportunity to measure the off-shell coupling strengths of the observed Higgs boson, as discussed in Refs. [103–106]. The $ZZ \rightarrow 4\ell$, $ZZ\rightarrow 2\ell 2\nu$ and $WW\rightarrow e\nu \mu \nu$ final states in the $\sqrt{s}=8$ TeV dataset are used in these measurements, detailed in Ref. [21]. Assuming the relevant Higgs boson coupling strengths are independent of the energy scale of Higgs boson production, a combination with the on-shell measurements can be interpreted as a constraint on the total width of the Higgs boson.

The analysis in the $ZZ\rightarrow 4\ell$ final state follows closely the Higgs boson measurements in the same final state, described in Sect. 2.2, with the same object definitions, event selections and background estimation methods. The off-peak region is defined to include the range $220\,\mathrm{GeV} < m_{4\ell } <1000\,\mathrm{GeV}$. Like the $H\rightarrow ZZ^*\rightarrow 4\ell$ analysis, the background is dominated by $q\bar{q}/gg\rightarrow ZZ$ production. A matrix-element-based discriminant [21] is constructed to enhance the $gg\rightarrow H^*\rightarrow ZZ$ signal and is used in a binned maximum-likelihood fit for the final result.

The analysis in the $ZZ\rightarrow 2\ell 2\nu$ channel follows closely the *ZH* analysis with the Higgs boson decaying to weakly interacting particles [28], with the same object definitions. As the analysis is performed inclusively in the number of jets in the final states, kinematic cuts are optimised accordingly. SM *ZZ* and *WZ* production are the major backgrounds. The transverse mass ($m_{\mathrm {T}}^{ZZ}$) [21], reconstructed from the momentum of the dilepton system and the missing transverse momentum, is chosen as the discriminating variable. Events in the range of $380\,\mathrm{GeV} < m_{\mathrm {T}}^{ZZ} <1000\,\mathrm{GeV}$ are used in a binned maximum likelihood fit for the final result.

The analysis in the $WW\rightarrow e\nu \mu \nu$ channel follows closely the Higgs boson measurements in the oppositely charged electron–muon pair final state, described in Sect. 2.3, with the same object definitions. The analysis is performed inclusively in the number of jets in the final state, and selections are optimised for the off-shell region with revised background estimation methods. Top quark pairs and *WW* events constitute the major backgrounds. In order to isolate the off-shell Higgs boson production while minimising sensitivity to higher-order QCD effects on $gg\rightarrow WW$ kinematics, a new variable $R_8$ [12], defined as the weighted combination of the dilepton mass and the transverse mass of the dilepton and $E_{\text {T}}^{\text {miss}}$ system, is constructed to select the signal region. The final results are obtained from the numbers of events observed in the data and expected from background processes in the signal region of $R_8>450$ GeV.

### Modifications of analyses

To ensure a consistent interpretation of all inputs in terms of Higgs boson coupling strengths, several minor modifications were made to the inputs of these combinations with respect to their previously published versions:

- The upper limits on the $H\rightarrow Z\gamma$ and $H\rightarrow \mu \mu$ decays and the results of the *ttH* searches in $H\rightarrow b\bar{b}$ and $ttH\rightarrow \mathrm{multilepton}$ decays have been updated to assume a Higgs boson mass of 125.36 GeV.
- In some individual analyses, cross-feed of other Higgs boson decays occurs: in the $VH,\,H \rightarrow WW^{*}$ selection cross-feed of $H \rightarrow \tau \tau$ and $H \rightarrow ZZ^{*}$ occurs (whereas this cross-feed is negligible in the ggF and VBF $H \rightarrow WW^{*}$analyses where a veto on the reconstructed $\tau \tau$ mass is applied). Similarly, there is cross-feed from $H \rightarrow WW^{*}$ in the $H \rightarrow \tau \tau$ analysis. In such cases, this cross-feed was treated as background in the relevant individual channel analyses. For the combinations described in this paper, such events are interpreted as signal from the corresponding Higgs boson decay.
- The rate of $gg\rightarrow ZH$ events in the *VH* channels is parameterised in terms of Higgs boson coupling strengths to *Z* bosons and top quarks, following the calculations of Ref. [58] for $\sqrt{s}=7$ and 8 TeV.
- The rate of *tH* events in all the *ttH* channels is parameterised in terms of Higgs boson coupling strengths to *W* bosons and top quarks.
- In the standalone analysis of the *ttH* channels, small contributions from Higgs boson decays to the $c\bar{c}$ and *gg* final states are explicitly modelled. To avoid spurious sensitivity due to these very small components in the combined analyses presented in this paper, both aforementioned decays are treated like $H \rightarrow b\bar{b}$ in the fits for the Higgs boson signal strength. In fits for Higgs boson coupling strengths, it is assumed that the coupling strengths of the $H\rightarrow c\bar{c}$ and $H\rightarrow gg$ decays scale as the $t\bar{t}\rightarrow H$ and $gg\rightarrow H$ couplings, respectively.
- Theoretical uncertainties from QCD scales in Higgs boson signal processes have been updated to be consistent with the latest recommendations [32] for $H \rightarrow WW^*,\, b\bar{b},\, \tau \tau$ and $Z\gamma$. No modifications were needed for the $H{\rightarrow \,}\gamma \gamma$ and $H \rightarrow ZZ^{*}$ channels.
- In channels where *bbH* production was not explicitly modelled, the signal strength of ggF is redefined to include this process. In channels where *bbH* was modelled explicitly ($H \rightarrow \gamma \gamma ,ZZ^*$), ggF and *bbH* production are correlated with their ratio fixed to the SM value, allowing a consistent treatment of *bbH* production across all channels. The impact of this average scaling on the results is negligible since, as can be seen in Table 3, the *bbH* production process has a cross section which is only 1 % of the ggF production in the SM.
- The off-shell analysis depends on the unknown *K*-factor from higher-order QCD corrections for the $gg \rightarrow VV$ background process. In the case of the very similar Higgs boson signal $gg\rightarrow H^*\rightarrow VV$ production process, a *K*-factor between 0.5 and 2 is expected, as discussed in Ref. [21]. The results are given as a function of the unknown ratio of the *K*-factors for $gg\rightarrow VV$ background and $gg\rightarrow H^*\rightarrow VV$ signal, $R^B_{H^*}$. The range 0.5–2.0 is chosen as a systematic uncertainty on $R^B_{H^*}$.

## Statistical procedure

The statistical treatment of the data is described in Refs. [107–111]. Hypothesis testing and confidence intervals are based on the $\Lambda (\mathbf {\alpha })$ profile likelihood ratio [112] test statistic. The test statistic depends on one or more parameters of interest $\mathbf {\alpha }$, such as the Higgs boson signal strength $\mu$ normalised to the SM expectation (Eq. (1)), Higgs boson mass $m_H$, coupling strength scale factors $\mathbf {\kappa }$ and their ratios $\mathbf {\lambda }$, as well as on additional parameters $\mathbf {\theta }$ that are not of interest,

4 $$\begin{aligned} \Lambda (\mathbf {\alpha }) = \frac{L\big (\mathbf {\alpha }\,,\,{\hat{\hat{{\varvec{\theta }}}}}(\mathbf {\alpha })\big )}{L(\hat{\mathbf {\alpha }},\hat{\mathbf {\theta }})} . \end{aligned}$$

The likelihood functions in the numerator and denominator of the above equation are built using sums of signal and background probability density functions (pdfs) of the discriminating variables, introduced in Sect. 2. The pdfs are derived from MC simulation for the signal and from both data and simulation for the background. Likelihood fits to the observed data are done for the parameters of interest. The single circumflex in Eq. (4) denotes the unconditional maximum-likelihood estimate of a parameter, i.e. both the parameters of interest and the nuisance parameters are varied to maximise the likelihood function. The double circumflex denotes a conditional maximum-likelihood estimate, i.e. an estimate for given fixed values of the parameters of interest $\mathbf {\alpha }$.

Systematic uncertainties and their correlations [107] are modelled by introducing nuisance parameters $\mathbf {\theta }$ described by likelihood functions associated with the estimate of the corresponding effect. Systematic uncertainties that affect multiple measurements are modelled with common nuisance parameters to propagate the effect of these uncertainties coherently to all measurements. Most experimental systematic uncertainties are modelled independently for the $\sqrt{s}=7$ and 8 TeV data samples, reflecting independent assessments of these uncertainties, but a subset of these uncertainties, e.g. material effects and some components of the jet energy scale, are considered common to the two data taking periods and are correspondingly described by a common set of nuisance parameters.

Components of theoretical uncertainties, scale uncertainties of a given Higgs boson production process as well as PDF-induced uncertainties, that affect the inclusive signal rate are described with common nuisance parameters in all channels, whereas components of theoretical uncertainties that affect the acceptance of individual channels are modelled with separate nuisance parameters for each decay channel. Specifically, since PDF-induced uncertainties and scale uncertainties are described by separate nuisance parameters, these uncertainties are effectively treated as uncorrelated. The PDF uncertainties of the inclusive rates are treated as correlated for *WH*, *ZH* and VBF production, as anti-correlated for $gg\rightarrow ZH$ and $qq\rightarrow ZH$ production and as uncorrelated for ggF and *ttH* production. A cross check with the full correlation matrix as given in Ref. [32] show no differences larger than 1 % for the most generic model (Sect. 5.5.3). Similarly, the effects of correlations between Higgs boson branching ratios and partial decay widths have been determined to be negligible, and are ignored in the combinations, except for the branching ratios to $WW^*$ and $ZZ^*$ which are treated as fully correlated. When results are provided with a breakdown of the systematic uncertainties in experimental and theoretical uncertainties, the theoretical uncertainties correspond to the influence of all nuisance parameters that can affect Higgs boson signal distributions, e.g. parton density functions related to Higgs boson production, QCD scale uncertainties related to Higgs boson production processes and uncertainties on the Higgs boson branching ratios. Theoretical uncertainties that exclusively affect background samples are included in the systematic uncertainty components.

The choice of the parameters of interest depends on the test under consideration, with the remaining parameters being “profiled”, i.e., similarly to nuisance parameters they are set to the values that maximise the likelihood function for the given fixed values of the parameters of interest.

Asymptotically, a test statistic $-2\ln \Lambda (\mathbf {\alpha })$ of several parameters of interest $\mathbf {\alpha }$ is distributed as a $\chi ^2$ distribution with *n* degrees of freedom, where *n* is the dimensionality of the vector $\mathbf {\alpha }$. In particular, the $100(1-\beta ) \ \%$ confidence level (CL) contours are defined by $-2\ln \Lambda (\mathbf {\alpha })<k_\beta$, where $k_\beta$ satisfies $P(\chi ^2_n > k_\beta ) = \beta$. For one degree of freedom the 68 % and 95 % CL intervals are given by $-2\ln \Lambda (\mathbf {\alpha })=1.0$ and 4.0, respectively. For two degrees of freedom the 68 and 95 % CL contours are given by $-2\ln \Lambda (\mathbf {\alpha })=2.3$ and 6.0, respectively. All results presented in the following sections are based on likelihood evaluations and give CL intervals under asymptotic approximation.^2^ For selected parameters of interest a physical boundary on the parameter values is included in the statistical interpretation. For example, branching ratio parameters can conceptually not be smaller than zero. The 95 % confidence interval quoted for such parameters is then based on the profile likelihood ratio restricted to the allowed region of parameter space; the confidence interval is defined by the standard $\chi ^2$ cutoff, which leads to some over-coverage near the boundaries.

For the measurements in the following sections the compatibility with the Standard Model, $p_\text {SM}$, is quantified using the *p*-value^3^ obtained from the profile likelihood ratio $\Lambda (\mathbf {\alpha }=\mathbf {\alpha }_\mathrm{SM})$, where $\mathbf {\alpha }$ is the set of parameters of interest and $\mathbf {\alpha }_\mathrm{SM}$ are their Standard Model values. For a given benchmark coupling model, $\mathbf {\alpha }$ is the set of Higgs boson coupling scale factors $\kappa _i$ and ratios of coupling scale factors $\lambda _{ij}$ probed by that model, where the indices *i*, *j* refer to the parameters of interest of the model (see Sect. 5). All other parameters are treated as independent nuisance parameters.

## Signal-strength measurements

This section discusses the measurements of the signal-strength parameter $\mu$ of different production modes and decay channels as well as their ratios for a fixed Higgs boson mass hypothesis of $m_H = 125.36$ GeV [23]. The signal-strength parameter is a measure of potential deviations from the SM prediction under the assumption that the Higgs boson production and decay kinematics do not change appreciably from the SM expectations. In particular, the transverse momentum and rapidity distributions of the Higgs boson are assumed to be those predicted for the SM Higgs boson by state-of-the-art event generators and calculations of each production process. This assumption is corroborated by studies such as the measurements of differential production cross sections [113, 114] and tests of spin and CP properties of the Higgs boson [24, 115].

For the discussion in this section, *bbH* is assumed to have the same signal strength as ggF, *tH* the same as *ttH*, and $gg\rightarrow ZH$ the same as $q\bar{q}\rightarrow ZH$, unless noted otherwise. The ggF and *bbH* processes lead to similar event signatures and no attempt is made to separate them in the analyses, thus the assumption of equal signal strength implies that the observed ggF signal is interpreted as a mixture of *bbH* and ggF events following their SM ratio of cross sections. The *ttH* and *tH* events have similar topologies. The $gg\rightarrow ZH$ process leads to the same final state as the $q\bar{q}\rightarrow ZH$ process. Whenever *WH* and *ZH* are combined into *VH*, their signal strengths are assumed to be the same.

### Global signal strength

**Fig. 2:**
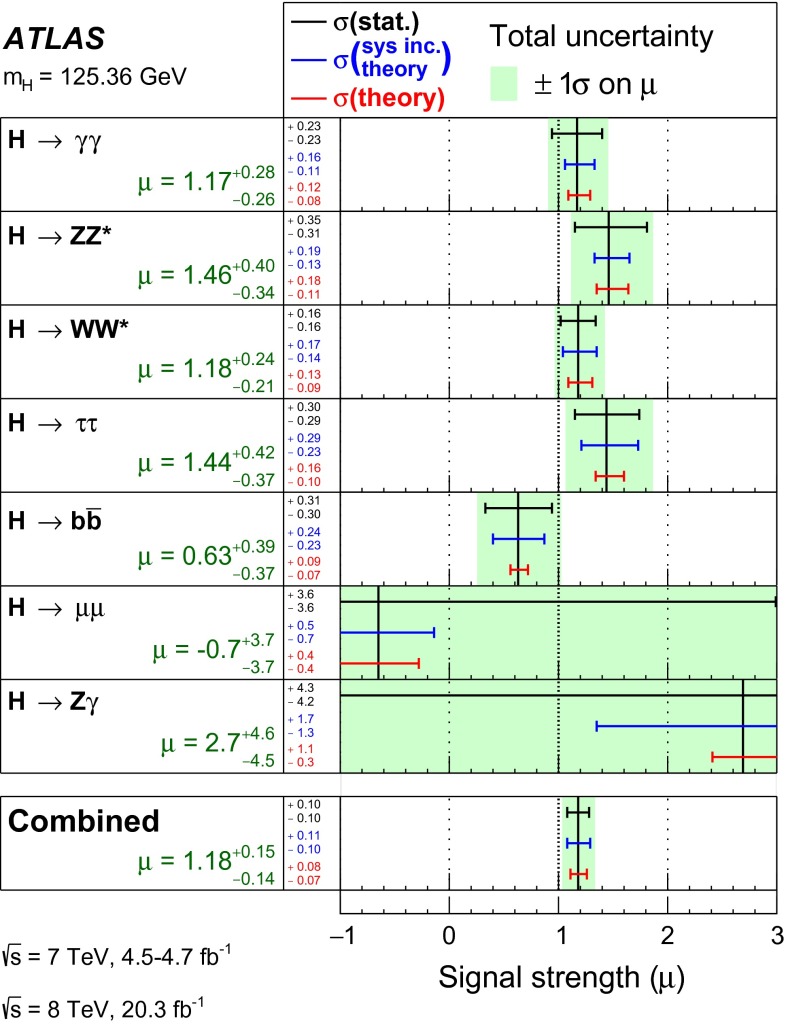
The observed signal strengths and uncertainties for different Higgs boson decay channels and their combination for $m_H=125.36$ GeV. Higgs boson signals corresponding to the same decay channel are combined together for all analyses, assuming SM values for the cross-section ratios of different production processes. The best-fit values are shown by the *solid vertical lines*. The total $\pm 1\sigma$ uncertainties are indicated by *green shaded bands*, with the individual contributions from the statistical uncertainty (*top*), the total (experimental and theoretical) systematic uncertainty (*middle*), and the signal theoretical uncertainty (*bottom*) on the signal strength shown as *horizontal error bars*

In Sect. 2, the published ATLAS measurements on Higgs boson production and decay modes based on individual final states as well as the changes since their publication are summarised. Figure 2 shows the updated measurements of the signal-strength parameter $\mu$ from a simultaneous fit to all decay channels analysed, assuming SM values for the cross-section ratios of different Higgs boson production processes (or equivalently all $\mu _i$’s of Eq. (2) are set to be equal). In the fit, the SM predictions of the signal yields are scaled by decay-dependent signal-strength parameters, independent of production processes. Compared to the separate measurements shown in Fig. 1, small changes are observed, resulting from the assignment of the Higgs boson yields in the *ttH* searches to appropriate decay channels, namely $H\rightarrow WW^*$, $H\rightarrow \tau \tau$ and $H\rightarrow b\bar{b}$.^4^ The central values all increase slightly due to the high observed signal-strength values of the *ttH* searches, but the uncertainties are barely improved because of the limited significance obtained for the *ttH* production process with the current dataset. The most significant change in the signal strength is observed for the $H\rightarrow b\bar{b}$ decay. The combination of the $VH(\rightarrow b\bar{b})$ analysis and the $ttH(\rightarrow b\bar{b})$ search leads to an observed (expected) significance of 1.8 (2.8) standard deviations for the $H\rightarrow b\bar{b}$ decay channel.

Assuming a multiplier common to all decay modes, signal-strength measurements of individual decay modes can be combined to give a global and more precise measurement, providing the simplest consistency test with the SM expectation. Combining all measurements using the profile likelihood ratio $\Lambda (\mu )$ results in a global signal-strength value of

$$\begin{aligned} \mu = 1.18\,^{+0.15}_{-0.14}=1.18 \pm 0.10\,(\mathrm{stat.})\pm 0.07\,(\mathrm{syst.})\,^{+0.08}_{-0.07}\,(\mathrm{theo.}), \end{aligned}$$

where the labels stat., syst. and theo. refer to statistical, systematic, and signal theoretical uncertainties, respectively. The signal theoretical uncertainty includes contributions from uncertainties in SM cross sections and branching ratios as well as in the modelling of the production and decays of the Higgs boson, as discussed in Sect. 3. The theoretical uncertainties of background processes are included in the uncertainty labelled as systematic uncertainty.

**Table 4:** Measured signal strengths $\mu$ at $m_H=125.36$ GeV and their total $\pm 1\sigma$ uncertainties for different production modes for the $\sqrt{s}=8$ TeV data and the combination with the $\sqrt{s}=7$ TeV data. The $\sqrt{s}=7$ TeV data do not have sufficient statistical power to yield meaningful measurements for individual production modes, but are included in the combination. Shown in the square brackets are uncertainty components: statistical (first), systematic (second) and signal theoretical (third) uncertainties. These results are derived using SM values for the ratios of branching ratios of different Higgs boson decay channels

Production process	Signal strength $\mu$ at $m_H=125.36$ GeV	
	$\sqrt{s}=8$ TeV	Combined $\sqrt{s}=7$ and 8 TeV
ggF	$1.23\,^{+0.25}_{-0.21}$ $\left[ {^{+0.16}_{-0.16}}\, {^{+0.10}_{-0.08}}\, {^{+0.16}_{-0.11}}\right]$	$1.23\,^{+0.23}_{-0.20}$ $\left[ {^{+0.14}_{-0.14}}\, {^{+0.09}_{-0.08}}\, {^{+0.16}_{-0.12}}\right]$
VBF	$1.55^{+0.39}_{-0.35}$ $\left[ {^{+0.32}_{-0.31}}\, {^{+0.17}_{-0.13}}\, {^{+0.13}_{-0.11}}\right]$	$1.23\pm 0.32$ $\left[ {^{+0.28}_{-0.27}}\, {^{+0.13}_{-0.12}}\, {^{+0.11}_{-0.09}}\right]$
*VH*	$0.93\pm 0.39$ $\left[ {^{+0.37}_{-0.33}}\, {^{+0.20}_{-0.18}}\, {^{+0.12}_{-0.06}}\right]$	$0.80\pm 0.36$ $\left[ {^{+0.31}_{-0.30}}\, {^{+0.17}_{-0.17}}\, {^{+0.10}_{-0.05}}\right]$
*ttH*	$1.62\pm 0.78$ $\left[ {^{+0.51}_{-0.50}}\, {^{+0.58}_{-0.54}}\, {^{+0.28}_{-0.10}}\right]$	$1.81\pm 0.80$ $\left[ {^{+0.52}_{-0.50}}\, {^{+0.58}_{-0.55}}\, {^{+0.31}_{-0.12}}\right]$

The uncertainty on the global signal strength has comparable statistical and systematic components and is significantly reduced compared to the individual measurements, as illustrated in Fig. 2. Here, the largest source of experimental systematic uncertainty is from background estimates in the analyses of individual channels. This result is consistent with the SM expectation of $\mu =1$, with a *p*-value of $18~\%$, All individual measurements of the signal-strength parameters are consistent and compatible with the combined value, with a *p*-value of $76~\%$.

Performing independent combinations of measurements at $\sqrt{s}=7$ and 8 TeV independently lead to signal-strength values of

$$\begin{aligned} \mu (7\,\mathrm{TeV})= & {} 0.75\,^{+0.32}_{-0.29}=0.75\,^{+0.28}_{-0.26}\,(\mathrm{stat.})\,^{+0.13}_{-0.11}\,(\mathrm{syst.})\,^{+0.08}_{-0.05}\,(\mathrm{theo.}),\ \mathrm{and} \\ \mu (8\,\mathrm{TeV})= & {} 1.28\,^{+0.17}_{-0.15}=1.28 \pm 0.11\,(\mathrm{stat.})\,^{+0.08}_{-0.07}\,(\mathrm{syst.})\,^{+0.10}_{-0.08}\,(\mathrm{theo.})\end{aligned}$$

at these two energies. The relative theoretical uncertainty of $\sim$7 % on the measured $\mu$ value at $\sqrt{s}=8$ TeV arises predominantly from the uncertainty on the total cross section, but is nevertheless smaller than the corresponding uncertainty of $\sim$9 % on the total SM cross section shown in Table 1, because $\mu$ is effectively a weighted average of the signal-strength measurements in all categories: the contributions from VBF and *VH* production, which have comparatively small theoretical uncertainties, have larger weights in this average than in the total cross section.

### Individual production processes

In addition to the signal strengths of different decay channels, the signal strengths of different production modes are also determined, exploiting the sensitivity offered by the use of event categories in the analyses of all channels.

**Fig. 3:**
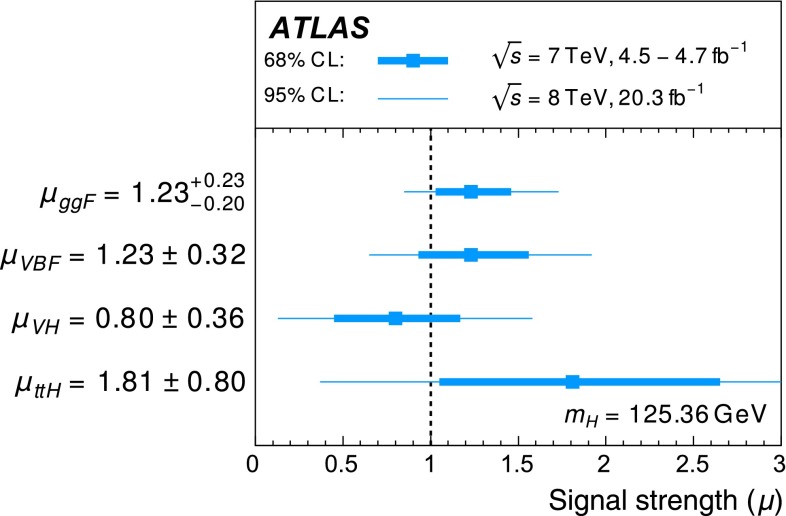
The best-fit signal-strength values of different production modes determined from the combined fit to the $\sqrt{s}=7$ and 8 TeV data. Higgs boson signals corresponding to the same production process but from different decay channels are combined together, assuming SM values for the ratios of the branching ratios of different Higgs boson decay channels. The *inner* and *outer error bars* correspond to 68  and 95 % CL intervals. Total uncertainties combining statistical, experimental and theoretical systematic uncertainties are shown

The Higgs boson production modes can be probed with four signal-strength parameters: $\mu _\mathrm{ggF}$, $\mu _\mathrm{VBF}$, $\mu _{VH}$ and $\mu _{ttH}$, one for each main production mode, combining Higgs boson signals from different decay channels under the assumption of SM values for the ratios of the branching ratios of different Higgs boson decays. This assumption is equivalent to set all $\mu _f's$ in Eq. (2) to be equal. The SM predictions of the signal yields are scaled by these four production-dependent parameters. The best-fit values of these parameters for the $\sqrt{s}=8$ TeV data separately and in combination with the $\sqrt{s}=7$ TeV data are shown in Table 4. Uncertainty components from statistics, systematics, and signal theory are also shown. The accuracy with which the uncertainties are broken down is limited by the precision of the fit and more importantly by the approximations made in individual analyses when neglecting uncertainties which are small with respect to, e.g., the statistical uncertainty. The $\sqrt{s}=7$ and 8 TeV combined values with their total uncertainties are also illustrated in Fig. 3. The $\sqrt{s}=7$ TeV data are included in the combinations only, as they have limited statistical power to distinguish between different production modes. The signal-strength measurements are in reasonable agreement with the SM predictions of unity. Although the results support the SM prediction of the *ttH* production (see Sect. 4.4), this production process remains to be firmly established in future LHC runs. Thus, a 95 % CL upper limit on its signal strength is also derived. Combining the results from various analyses with sensitivity to *ttH* production, the observed and expected limits are $\mu _{ttH}<3.2$ and $1.4$, respectively.

**Table 5:** Measured cross sections of different Higgs boson production processes at $\sqrt{s}=8$ TeV for $m_H=125.36$ GeV obtained from the signal-strength values of Table 4. Their SM predictions can be found in Table 1. Shown in the square brackets are uncertainty components: statistical (first), systematic (second) and signal theoretical (third) uncertainties. The theoretical uncertainties here arise from the modelling of Higgs boson production and decays. These results are derived using the SM values of the Higgs boson decay branching ratios

Production process	Cross section (pb) at $\sqrt{s}=8$ TeV
ggF	$23.9\pm 3.6$ $\left[ ^{+3.1}_{-3.1}\, {^{+1.9}_{-1.6}}\, {^{+1.0}_{-1.0}}\right]$
VBF	$2.43\pm 0.58$ $\left[ ^{+0.50}_{-0.49}\, {^{+0.27}_{-0.20}}\, {^{+0.19}_{-0.16}}\right]$
*VH*	$1.03\pm 0.53$ $\left[ ^{+0.37}_{-0.36}\, {^{+0.22}_{-0.20}}\, {^{+0.13}_{-0.06}}\right]$
*ttH*	$0.24\pm 0.11$ $\left[ ^{+0.07}_{-0.07}\, {^{+0.08}_{-0.08}}\, {^{+0.01}_{-0.01}}\right]$

The signal-strength measurements shown in Table 4 are extrapolated to total cross-section measurements for each production process, as shown in Table 5 for $\sqrt{s}=8$ TeV, with the further assumption of SM values for the Higgs boson decay branching ratios. The theoretical uncertainties on the absolute values of the SM Higgs boson production cross sections are thereby removed, but significant theoretical uncertainties remain, related to the modelling of the Higgs boson production and of the acceptance of the event selection. One can sum the different cross sections to obtain an overall extrapolated cross section for Higgs boson production. The measurement is performed at $\sqrt{s}=7$ TeV as well despite of the limited statistical power of the dataset. The resulting total Higgs boson production cross sections at the two energies are

$$\begin{aligned} \sigma _H(7\,\mathrm{TeV})= & {} 22.1\,^{+7.4}_{-6.0}\,\mathrm{pb} \\= & {} 22.1\,^{+6.7}_{-5.3}\,(\mathrm{stat.})\,^{+2.7}_{-2.3}\,(\mathrm{syst.})\,^{+1.9}_{-1.4}\,(\mathrm{theo.})\, \mathrm{pb},\ \mathrm{and} \\ \sigma _H(8\,\mathrm{TeV})= & {} 27.7\pm 3.7\,\mathrm{pb} \\= & {} 27.7\pm 3.0\, (\mathrm{stat.})\,^{+2.0}_{-1.7}\,(\mathrm{syst.})\,^{+1.2}_{-0.9}\,(\mathrm{theo.})\, \mathrm{pb}\,, \end{aligned}$$

to be compared with the theoretical predictions of $17.4\pm 1.6$ pb at $\sqrt{s}=7$ TeV and $22.3\pm 2.0$ pb at $\sqrt{s}=8$ TeV, as shown in Table 1.

**Fig. 4:**
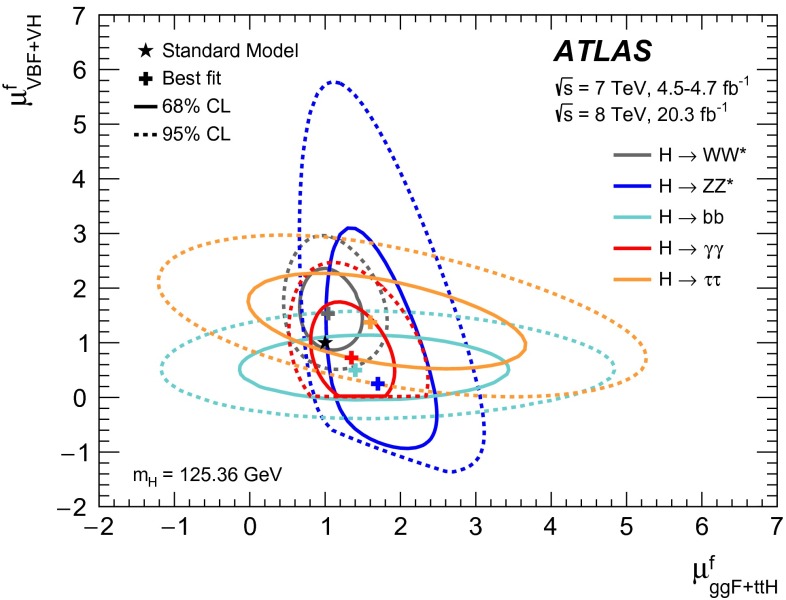
Likelihood contours in the $(\mu ^f_{\mathrm {ggF}+ttH}, \mu ^f_{\mathrm {VBF}+VH})$ plane for a Higgs boson mass $m_H=125.36$ GeV measured separately for $H\rightarrow WW^*,\, ZZ^*,\, b\bar{b},\, \gamma \gamma$ and $\tau \tau$ decays. SM values are assumed for the relative contributions between ggF and *ttH* and between VBF and *VH* production. The straight lower portions of the $H\rightarrow \gamma \gamma$ and $H\rightarrow ZZ^*\rightarrow 4\ell$ contours are due to the small numbers of events in these channels and the requirement of a positive probability density function. The best-fit values to the data (*plus symbol*) and the $68~\%$ (*full*) and $95~\%$ (*dashed*) CL contours are indicated, as well as the SM expectation $(*)$

These cross sections are different from what one would naively expect from the global signal-strength values discussed in Sect. 4.1, particularly for $\sqrt{s}=7$ TeV. The differences are largely the result of analysis categorisation. Categories often explore production processes or phase-space regions with distinct signal-event topologies. The resulting high signal-to-background ratios can significantly improve the precision of the signal-strength measurements. However, these categories often account for small fractions of the production cross section and thus have limited impact on the total cross-section measurement, which is dominated by processes with larger expected cross sections. One good example is the VBF category. It contributes significantly to the global signal-strength measurement, but has a relatively minor impact on the total cross-section measurement.

### Boson and fermion-mediated production processes

The Higgs boson production processes can be categorised into two groups according to the Higgs boson couplings to fermions (ggF and *ttH*) or vector bosons (VBF and *VH*). Potential deviations from the SM can be tested with two signal-strength parameters, $\mu ^f_{\text {ggF}+ttH}\equiv (\mu ^f_{\text {ggF}}=\mu ^f_{ttH}$) and $\mu ^f_{\text {VBF}+VH}\equiv (\mu ^f_{\text {VBF}}=\mu ^f_{VH})$ for each decay channel *f*, assuming SM values for the ratio of ggF and *ttH* cross sections and the ratio of VBF and VH cross sections. Signal contaminations from one group to another, e.g. ggF events with two jets passing the VBF selection, are taken into account in the simultaneous fit. The 68 and 95 % CL two-dimensional contours of $\mu ^f_{\text {ggF}+ttH}$ and $\mu ^f_{\text {VBF}+VH}$ of the five main decay channels are shown in Fig. 4. The measurements of $H\rightarrow \mu \mu$ and $H\rightarrow Z\gamma$ decays have relatively poor sensitivities and are therefore not included in the figure. The cutoff in the contours of the $H{\rightarrow \,}\gamma \gamma$ and $H \rightarrow ZZ^{*}$ decays is caused by the expected sum of signal and background yields in one of the contributing measurements going below zero in some regions of the parameter space shown in Fig. 4. The SM expectation of $\mu ^f_{\text {ggF}+ttH}=1$ and $\mu ^f_{\text {VBF}+VH}=1$ is within the 68 % CL contour of most of these measurements.

**Table 6:** The best-fit values and their uncertainties for the ratio $R_{ff}$ of cross sections for the vector-boson- and fermion-mediated production processes relative to their SM values at $m_H=125.36$ GeV for the individual decay channels and their combination. Shown in the square brackets are uncertainty components: statistical (first), systematic (second) and signal theoretical (third) uncertainties. These results are independent of the Higgs boson decay branching ratios

Decay channel	Cross-section ratio $R_{ff}$
$H\rightarrow \gamma \gamma$	$0.56\,^{+0.66}_{-0.45}\ \ \left[ {^{+0.62}_{-0.42}}\, {^{+0.15}_{-0.09}}\, {^{+0.18}_{-0.15}}\right]$
$H\rightarrow ZZ^*$	$0.18\,^{+1.20}_{-0.52}\ \ \left[ {^{+1.16}_{-0.50}}\, {^{+0.23}_{-0.05}}\, {^{+0.23}_{-0.16}}\right]$
$H\rightarrow WW^*$	$1.47\,^{+0.80}_{-0.54}\ \ \left[ {^{+0.63}_{-0.47}}\, {^{+0.37}_{-0.19}}\, {^{+0.31}_{-0.18}}\right]$
$H\rightarrow \tau \tau$	$0.81\,^{+2.19}_{-0.49}\ \ \left[ {^{+1.36}_{-0.41}}\, {^{+1.68}_{-0.15}}\, {^{+0.39}_{-0.23}}\right]$
$H\rightarrow b\bar{b}$	$0.33\,^{+1.03}_{-0.25}\ \ \left[ {^{+0.39}_{-0.20}}\, {^{+0.94}_{-0.14}}\, {^{+0.18}_{-0.06}}\right]$
Combined	$0.96\,^{+0.43}_{-0.31}\ \ \left[ {^{+0.33}_{-0.26}}\, {^{+0.20}_{-0.13}}\, {^{+0.18}_{-0.10}}\right]$

The relative production cross sections of the processes mediated by vector bosons and by fermions can be tested using the ratio $\mu ^f_{\text {VBF}+VH}/\mu ^f_{\text {ggF}+ttH}$. When measured separately for each decay channel, this ratio reduces to the ratio of production cross sections because the Higgs boson decay branching ratios cancel and is equivalent to the ratio of $\mu _i$ defined in Sect. 4.1, i.e.,

5 $$\begin{aligned} \frac{\mu ^f_{\text {VBF}+VH}}{\mu ^f_{\text {ggF}+ttH}} = \frac{\sigma _{\text {VBF}+VH}/\sigma _{\text {ggF}+ttH}}{\left[ \sigma _{\text {VBF}+VH}/\sigma _{\text {ggF}+ttH}\right] }_\mathrm{SM}=\frac{\mu _{\mathrm{VBF}+VH}}{\mu _{\mathrm{ggF}+ttH}} \equiv R_{ff}. \end{aligned}$$

**Fig. 5:**
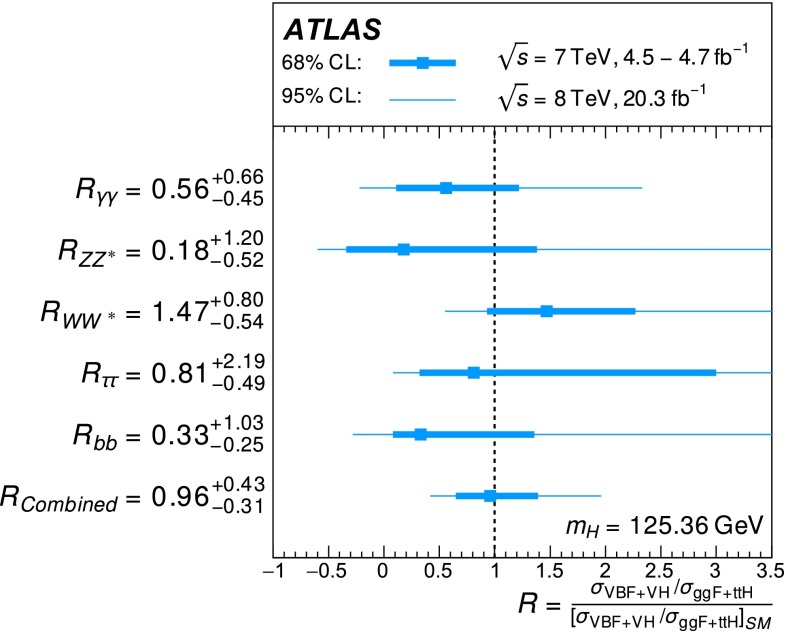
The ratios of cross sections for the vector-boson- and fermion-mediated processes relative to their SM values at $m_H=125.36$ GeV, measured in the individual Higgs boson decay final states and their combination, $R_\mathrm{Combined}$ (see text). The *inner* and *outer error bars* represent 68 and 95 % CL intervals, combining statistical and systematic uncertainties. These measurements are independent of Higgs boson decay branching ratios

The observed ratios are shown in Table 6 and illustrated in Fig. 5 for the five main decay channels. The signal-strength parameter $\mu ^f_{\mathrm{ggF}+ttH}$ of each decay channel is profiled in the fit. The combination of these measurements yields an overall value of the ratio of cross sections for the vector-boson- and fermion-mediated processes (relative to its SM prediction):

$$\begin{aligned} R_\mathrm{Combined} =0.96\,^{+0.43}_{-0.31}=0.96\,^{+0.33}_{-0.26}\,(\mathrm{stat.})\,^{+0.20}_{-0.13}\,(\mathrm{syst.})\,^{+0.18}_{-0.10}\,(\mathrm{theo.}). \end{aligned}$$

### Ratios of production cross sections and partial decay widths

At the LHC, the Higgs boson production cross sections and decay branching ratios cannot be separately determined in a model-independent way as only their products are measured. However, the ratios of cross sections and ratios of branching ratios can be disentangled without any assumptions, within the validity of the narrow width approximation of the Higgs boson. By normalising to the cross section of the $gg\rightarrow H\rightarrow WW^*$ production process, $\sigma (gg\rightarrow H\rightarrow WW^*)$, the yields of other Higgs boson production modes and decay channels can be parameterised using the ratios of cross sections and ratios of branching ratios. For the production and decay $i\rightarrow H\rightarrow f$, the yield is then

6 $$\begin{aligned}&\sigma _i\cdot \mathrm{BR}_f = \left( \sigma _\mathrm{ggF}\cdot \mathrm{BR}_{WW^*}\right) \times \left( \frac{\sigma _i}{\sigma _\mathrm{ggF}}\right) \times \left( \frac{\mathrm{BR}_f}{\mathrm{BR}_{WW^*}}\right) \nonumber \\&\quad = \sigma (gg\rightarrow H\rightarrow WW^*)\times \left( \frac{\sigma _i}{\sigma _\mathrm{ggF}}\right) \times \left( \frac{\Gamma _f}{\Gamma _{WW^*}}\right) . \end{aligned}$$

The ratio of branching ratios in the above equation is substituted by the equivalent ratio of partial decay widths. The ratios extracted from the measured yields are independent of theoretical predictions on the inclusive cross sections and partial decay widths (and thus branching ratios). Furthermore, many experimental systematic uncertainties cancel in the ratios. The residual theoretical uncertainties are related to the modelling of the Higgs boson production and decay, which impacts the signal acceptance calculations. The $gg\rightarrow H\rightarrow WW^*$ process is chosen as the reference because it has both the smallest statistical and overall uncertainties, as shown in Fig. 2.

**Fig. 6:**
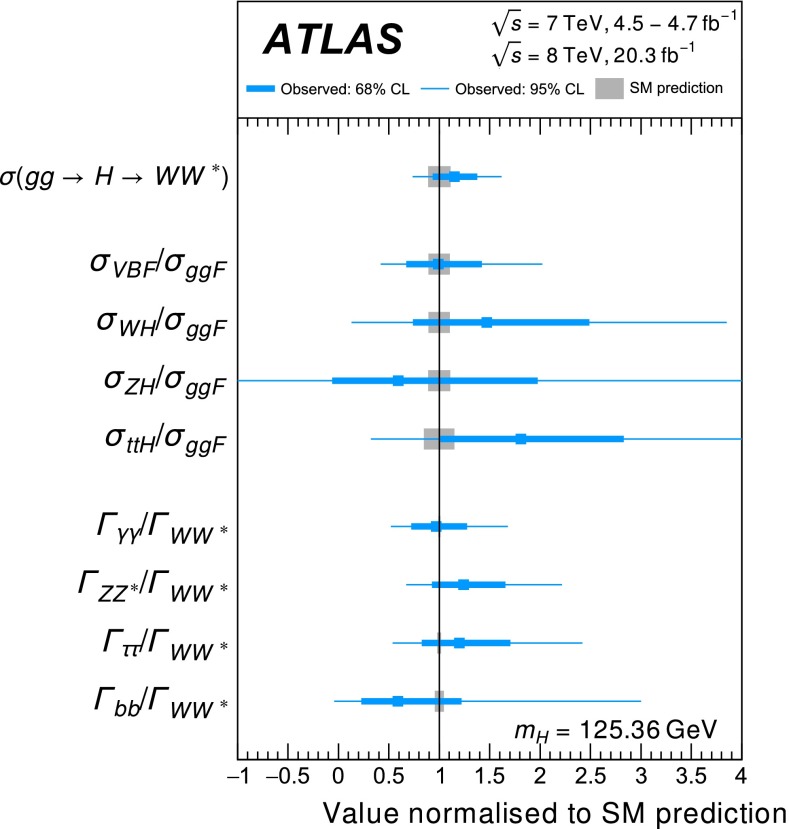
The $gg\rightarrow H\rightarrow WW^*$ cross section, ratios of cross sections and of partial decay widths relative to their SM values at $m_H=125.36$ GeV from the combined analyses of the $\sqrt{s}=7$ and 8 TeV data. The *inner* and *outer error bars* on the measurements are 68 and 95 % CL intervals. The SM predictions are shown as the *vertical line* at unity with *grey bands* representing theoretical uncertainties on the ratios of inclusive cross sections and of partial decay widths

The $\sqrt{s}=7$ and 8 TeV data are fitted with $\sigma (gg\rightarrow H\rightarrow WW^*)$, $\sigma _i/\sigma _\mathrm{ggF}$ and $\Gamma _f/\Gamma _{WW^*}$ as parameters of interest and the results are listed in Table 7, together with the SM predictions [32]. The results after normalising to their SM values are illustrated in Fig. 6. The results of $\sigma (gg\rightarrow H\rightarrow WW^*)$ and $\sigma _i/\sigma _\mathrm{ggF}$ from the combined analysis of the $\sqrt{s}=7$ and 8 TeV data are shown for $\sqrt{s}=8$ TeV, assuming the SM values for $\sigma _i(7~\mathrm{TeV})/\sigma _i(8~\mathrm{TeV})$. The *WH* and *ZH* production processes are treated independently in the fit to allow for direct comparisons with theoretical predictions. The searches for $H\rightarrow \mu \mu$ and $H\rightarrow Z\gamma$ decays are included in the fit, but the current datasets do not result in sensitive measurements for these two decays. Therefore only 95 % CL upper limits are derived, namely $0.006$ for $\Gamma _{\mu \mu }/\Gamma _{WW^*}$ and $0.078$ for $\Gamma _{Z\gamma }/\Gamma _{WW^*}$. The *p*-value of the compatibility between the data and the SM predictions is found to be $80~\%$.

**Table 7:** Best-fit values of $\sigma (gg\rightarrow H\rightarrow WW^*)$, $\sigma _i/\sigma _\mathrm{ggF}$ and $\Gamma _f/\Gamma _{WW^*}$ for a Higgs boson with $m_H=125.36$ GeV from the combined analysis of the $\sqrt{s}=7$ and 8 TeV data. The cross-section ratios are given for $\sqrt{s}=8$ TeV assuming the SM values for $\sigma _i(7~\mathrm{TeV})/\sigma _i(8~\mathrm{TeV})$. Shown in square brackets are uncertainty components: statistical (first), systematic (second) and signal theoretical (third) uncertainties. The SM predictions [32] are shown in the last column

Parameter	Best-fit value	SM prediction
$\sigma (gg\rightarrow H\rightarrow WW^*)$ (pb)	$4.86\,^{+0.95}_{-0.90}$ $\left[ ^{+0.76}_{-0.74}\,^{+0.52}_{-0.48}\,^{+0.26}_{-0.18}\right]$	$4.22\pm 0.47$
$\sigma _\mathrm{VBF}/\sigma _\mathrm{ggF}$	$0.081\,^{+0.035}_{-0.026}$ $\left[ ^{+0.031}_{-0.024}\,^{+0.016}_{-0.010}\,^{+0.008}_{-0.005}\right]$	$0.082\pm 0.009$
$\sigma _{WH}/\sigma _\mathrm{ggF}$	$0.053\,^{+0.037}_{-0.026}$ $\left[ ^{+0.032}_{-0.023}\,^{+0.018}_{-0.012}\,^{+0.008}_{-0.004}\right]$	$0.036\pm 0.004$
$\sigma _{ZH}/\sigma _\mathrm{ggF}$	$0.013\,^{+0.030}_{-0.014}$ $\left[ ^{+0.021}_{-0.013}\,^{+0.020}_{-0.005}\,^{+0.005}_{-0.002}\right]$	$0.021\pm 0.002$
$\sigma _{ttH}/\sigma _\mathrm{ggF}$	$0.012\,^{+0.007}_{-0.005}$ $\left[ ^{+0.005}_{-0.004}\,^{+0.004}_{-0.003}\,^{+0.0014}_{-0.0005}\right]$	$0.007\pm 0.001$
$\Gamma _{\gamma \gamma }/\Gamma _{WW^*}$	$0.010\,^{+0.003}_{-0.003}$ $\left[ ^{+0.003}_{-0.002}\,^{+0.002}_{-0.001}\,^{+0.0006}_{-0.0004}\right]$	$0.01036\pm 0.00011$
$\Gamma _{ZZ^*}/\Gamma _{WW^*}$	$0.15\,^{+0.05}_{-0.04}$ $\left[ ^{+0.046}_{-0.036}\,^{+0.022}_{-0.013}\,^{+0.008}_{-0.005}\right]$	$0.124\,\pm <\!0.001$
$\Gamma _{\tau \tau }/\Gamma _{WW^*}$	$0.34\,^{+0.14}_{-0.11}$ $\left[ ^{+0.112}_{-0.090}\,^{+0.084}_{-0.053}\,^{+0.032}_{-0.017}\right]$	$0.285\pm 0.006$
$\Gamma _{bb}/\Gamma _{WW^*}$	$1.53\,^{+1.64}_{-0.94}$ $\left[ ^{+1.17}_{-0.69}\,^{+1.11}_{-0.63}\,^{+0.30}_{-0.12}\right]$	$2.60\pm 0.12$

The results exhibit a few interesting features that are worth mentioning. As a multiplicative factor common to all rates in this parameterisation, $\sigma (gg\rightarrow H\rightarrow WW^*)$ is pulled up in the fit to accommodate the observed large global signal-strength value (Sect. 4.1). The best-fit value of $\sigma (gg\rightarrow H\rightarrow WW^*)$ is approximately 15 % above the SM prediction, to be compared to the significantly lower value of $0.98\,^{+0.29}_{-0.26}$, found from the stand-alone measurement from the $H\rightarrow WW^*$ decay (see Fig. 1). Moreover, there are by construction large anti-correlations between $\sigma (gg\rightarrow H\rightarrow WW^*)$, $\sigma _i/\sigma _\mathrm{ggF}$ and $\Gamma _f/\Gamma _{WW^*}$.

Table 8 shows the observed and expected significances in units of standard deviations of the VBF, *WH*, *ZH* and *ttH* production processes. Listed under *VH* are the combined significances of *WH* and *ZH* production, assuming the SM value for their relative cross sections. The significance is calculated from a likelihood scan, where the contributions from other processes are fixed at their best-fit values. As the $gg\rightarrow H\rightarrow WW^*$ process is chosen as the reference, the significances are calculated using the observable $\sigma (gg\rightarrow H\rightarrow WW^*)$ for the ggF process and the cross-section ratios $\sigma _i/\sigma _\mathrm{ggF}$ for all other processes. The cross-section ratios are independent of the Higgs boson decay branching ratios and have the advantage of the cancellation of many experimental uncertainties. The result provides an unequivocal confirmation of the gluon fusion production of the Higgs boson with its significance exceeding well above five standard deviations. Furthermore, the result also offers strong evidence, at $4.3$ standard deviations, of vector-boson fusion production and supports the SM assumptions of production in association with vector bosons or a pair of top quarks.

An alternative parameterisation normalising the ratios of cross sections and of branching ratios to their SM values is presented in Appendix A.

## Coupling-strength fits

In the previous section signal-strength parameter $\mu _{i}^{f}$ for a given Higgs boson production or decay mode is discussed. For a measurement of Higgs boson coupling strengths, production and decay modes cannot be treated independently, as each observed process involves at least two Higgs boson coupling strengths. Scenarios with a consistent treatment of coupling strengths in production and decay modes are studied in this section. All uncertainties on the best-fit values shown take into account both the experimental and theoretical systematic uncertainties. For selected benchmark models a breakdown of parameter uncertainties in statistical uncertainties and in experimental and theoretical systematic uncertainties is presented.

### Framework for coupling-strength measurements

Following the leading-order (LO) tree-level-motivated framework and benchmark models recommended in Ref. [32], measurements of Higgs boson coupling-strength scale factors $\kappa _{j}$ are implemented for the combination of all analyses and channels summarised in Table 3.

**Table 8:** The observed and expected significances in units of standard deviations for different Higgs boson production processes except ggF production which is well established (see text). The significances of *VH* production are obtained by combining the *WH* and *ZH* processes, assuming the SM value for their relative cross sections. All significances are calculated under the asymptotic approximation [112]

Process	VBF	*ttH*	*WH*	*ZH*	*VH*
Observed	4.3	2.5	2.1	0.9	2.6
Expected	3.8	1.5	2.0	2.1	3.1

#### Structure and assumptions of the framework for benchmark models

The framework is based on the assumption that the signals observed in the different channels originate from a single narrow resonance with a mass near $125.36~ \mathrm{GeV}$. The case of several, possibly overlapping, resonances in this mass region is not considered. Unless otherwise noted, the Higgs boson production and decay kinematics are assumed to be compatible with those expected for a SM Higgs boson, similar to what was assumed for the signal-strength measurements of Sect. 4.

The width of the assumed Higgs boson near $125.36~ \mathrm{GeV}$ is neglected in the Higgs boson propagator, i.e. the zero-width approximation is used. In this approximation, the cross section $\sigma ( i \rightarrow H\rightarrow f )$ for on-shell measurements can always be decomposed as follows:

7 $$\begin{aligned} \sigma ( i \rightarrow H \rightarrow f ) = \frac{\sigma _{ i }(\kappa _{j})\cdot \Gamma _{ f }(\kappa _{j})}{\Gamma _{{ H}}(\kappa _{j})} \end{aligned}$$

where $\sigma _{ i }$ is the Higgs boson production cross section through the initial state $i$, $\Gamma _{ f }$ its the partial decay width into the final state $f$ and $\Gamma _{{ H}}$ the total width of the Higgs boson. The index *j* runs over all Higgs boson couplings. The components of $\sigma _{ i }$, $\Gamma _{ f }$, and $\Gamma _{{ H}}$ of Eq. (7) are expressed in scale factors $\kappa _{j}$ of the Higgs boson coupling strengths to other particles *j* that are motivated by the leading-order processes that contribute to production or decay, and are detailed in Sect. 5.1.2. All scale factors are defined such that a value of $\kappa _{j}=1$ corresponds to the best available SM prediction, including higher-order QCD and EW corrections. This higher-order accuracy is generally lost for $\kappa _{j}\ne 1$, nevertheless higher-order QCD corrections approximately factorise with respect to coupling rescaling and are accounted for wherever possible.

Modifications of the coupling scale factors change the Higgs boson width $\Gamma _{{ H}}(\kappa _j)$ by a factor $\kappa _{{ H}}^2(\kappa _{j})$ with respect to the SM Higgs boson $\Gamma _{{ H}}^\mathrm{SM}$,

$$\begin{aligned} \Gamma _{{ H}}(\kappa _j) = \kappa _{{ H}}^2(\kappa _{j})\cdot \Gamma _{{ H}}^\mathrm{SM} , \end{aligned}$$

where $\kappa _{{ H}}^2(\kappa _{j})$ is the sum of the scale factors $\kappa _{j}^{2}$ weighted by the corresponding SM branching ratios. The total width of the Higgs boson increases beyond modifications of $\kappa _{j}$ if invisible or undetected Higgs boson decays^5^ occur that are not present in the SM. Including a Higgs boson branching fraction $\mathrm {BR_{i.,u.}}$ to such invisible or undetected decays, the full expression for the assumed Higgs boson width becomes

8 $$\begin{aligned} \Gamma _{{ H}}(\kappa _j,\mathrm {BR_{i.,u.}}) = \frac{\kappa _{{ H}}^2(\kappa _{j})}{(1-\mathrm {BR_{i.,u.}})} \Gamma _{{ H}}^\mathrm{SM}. \end{aligned}$$

As $\mathrm {BR_{i.,u.}}$ scales all observed cross-sections of on-shell Higgs boson production $\sigma ( i \rightarrow H\rightarrow f )$, some assumption about invisible decays must be made to be able to interpret these measurements in terms of absolute coupling-strength scale factors $\kappa _{j}$. The signal-strength measurements of off-shell Higgs boson production [21], on the other hand, is assumed to only depend on the coupling-strength scale factors and not on the total width  [103, 104], i.e.

9 $$\begin{aligned} \sigma ^\mathrm{off}( i \rightarrow H^{*}\rightarrow f ) \sim \kappa _{i,\mathrm{off}}^2 \cdot \kappa _{f,\mathrm{off}}^2 \end{aligned}$$

where the additional assumption of non-running coupling-strength scale factors, $\kappa _{j,\mathrm{off}} = \kappa _{j,\mathrm{on}}$ allows $\Gamma _{H}$ to be constrained using using Eq. (8), from a simultaneous measurement of on-shell and off-shell measurements. While this assumption of non-running coupling-strength scale factors cannot hold universally for ggF and VBF production without violating unitarity, it is assumed to hold in the region of phase space of the off-shell $H^*\rightarrow WW$ and $H^*\rightarrow ZZ$ measurements described in Sect. 2.9 which is relatively close to the on-shell regime [116]. Alternatively, ratios of coupling-strength scale factors can be measured without assumptions on the Higgs boson total width, as the identical contributions of $\Gamma _{{ H}}$ to each coupling strength cancel in any ratio of these.

Finally, only modifications of coupling strengths, i.e. of absolute values of coupling strengths, are taken into account, while the tensor structure of the couplings is assumed to be the same as in the SM. This means in particular that the observed state is assumed to be a CP-even scalar as in the SM. This assumption was tested by both the ATLAS [24] and CMS [115] Collaborations.

**Table 9:** Overview of Higgs boson production cross sections $\sigma _{i}$, the Higgs boson partial decay widths $\Gamma _{f}$ and the Higgs boson total width $\Gamma _{{ H}}$. For each production or decay mode the scaling of the corresponding rate in terms of Higgs boson coupling-strength scale factors is given. For processes where multiple amplitudes contribute, the rate may depend on multiple Higgs boson coupling-strength scale factors, and interference terms may give rise to scalar product terms $\kappa _{i}\kappa _{j}$ that allow the relative sign of the coupling-strength scale factors $\kappa _{i}$ and $\kappa _{j}$ to be determined. Expressions originate from Ref. [32], except for $\sigma (gg\rightarrow ZH$) (from Ref. [58]) and $\sigma (gb \rightarrow WtH)$ and $\sigma (qb \rightarrow tHq^\prime )$ (calculated using Ref. [82]). The expressions are given for $\sqrt{s} = 8\,\mathrm{TeV}$ and $m_H = 125.36\, \mathrm{GeV}$ and are similar for $\sqrt{s} = 7\,\mathrm{TeV}$. Interference contributions with negligible magnitudes have been omitted in this table

Production	Loops	Interference	Expression in fundamental coupling-strength scale factors
$\sigma (\text {ggF})$	$\checkmark$	*b*–*t*	$\kappa _{{ g}}^2 \sim$ $1.06 \cdot \kappa _{{ t}}^2 + 0.01 \cdot \kappa _{{ b}}^2 - 0.07\cdot \kappa _{{ t}}\kappa _{{ b}}$
$\sigma (\text {VBF})$	–	–	$\sim$ $0.74 \cdot \kappa _{{ W}}^2 + 0.26 \cdot \kappa _{{ Z}}^2$
$\sigma (WH)$	–	–	$\sim$ $\kappa _{{ W}}^2$
$\sigma (q\bar{q}\rightarrow ZH)$	–	–	$\sim$ $\kappa _{{ Z}}^2$
$\sigma (gg\rightarrow ZH)$	$\checkmark$	$Z-t$	$\kappa _{{ g}{ g}{ Z}{ H}}^2 \sim$ $2.27\cdot \kappa _{{ Z}}^2 + 0.37 \cdot \kappa _{{ t}}^2 - 1.64 \cdot \kappa _{{ Z}}\kappa _{{ t}}$
$\sigma (bbH)$	–	–	$\sim$ $\kappa _{{ b}}^2$
$\sigma (ttH)$	–	–	$\sim$ $\kappa _{{ t}}^2$
$\sigma (gb \rightarrow WtH)$	–	$W - t$	$\sim$ $1.84\cdot \kappa _{{ t}}^2 + 1.57 \cdot \kappa _{{ W}}^2 - 2.41 \cdot \kappa _{{ t}}\kappa _{{ W}}$
$\sigma (qb \rightarrow tHq^\prime )$	–	$W - t$	$\sim$ $3.4 \cdot \kappa _{{ t}}^2 + 3.56 \cdot \kappa _{{ W}}^2 - 5.96 \cdot \kappa _{{ t}}\kappa _{{ W}}$
Partial decay width			
$\Gamma _{b\bar{b}}$	–	–	$\sim$ $\kappa _{{ b}}^2$
$\Gamma _{WW}$	–	–	$\sim$ $\kappa _{{ W}}^2$
$\Gamma _{ZZ}$	–	–	$\sim$ $\kappa _{{ Z}}^2$
$\Gamma _{\tau \tau }$	–	–	$\sim$ $\kappa _{\tau }^2$
$\Gamma _{\mu \mu }$	–	–	$\sim$ $\kappa _{\mu }^2$
$\Gamma _{\gamma \gamma }$	$\checkmark$	$W - t$	$\kappa _{{\gamma }}^2 \sim$ $1.59 \cdot \kappa _{{ W}}^2 + 0.07 \cdot \kappa _{{ t}}^2 -0.66 \cdot \kappa _{{ W}} \kappa _{{ t}}$
$\Gamma _{Z\gamma }$	$\checkmark$	$W - t$	$\kappa _{{ Z}{\gamma }}^2 \sim$ $1.12 \cdot \kappa _{{ W}}^2 + 0.00035 \cdot \kappa _{{ t}}^2 - 0.12\cdot \kappa _{{ W}}\kappa _{{ t}}$
Total decay width			
$\Gamma _{{ H}}$	$\checkmark$	$$\begin{array}{cc}W-t\\ b-t\end{array}$$	$\kappa _{{ H}}^2 \sim$ $$\begin{array}{lll}0.57 \cdot \kappa _{{ b}}^2 + 0.22 \cdot \kappa _{{ W}}^2 + 0.09 \cdot \kappa _{{ g}}^2 + \\ 0.06 \cdot \kappa _{{\tau }}^2 + 0.03 \cdot \kappa _{{ Z}}^2 + 0.03 \cdot \kappa _{{ c}}^2 + \\ 0.0023 \cdot \kappa _{{\gamma }}^2 + 0.0016 \cdot \kappa _{{ Z}{\gamma }}^2 + 0.00022 \cdot \kappa _{{\mu }}^2\end{array}$$

#### Characterisation of the input measurements in terms of coupling strengths

The combined input channels described in Table 3 probe eight different production processes: $\sigma (\text {ggF})$, $\sigma (\text {VBF})$, $\sigma (WH)$, $\sigma (q\bar{q}\rightarrow ZH)$, $\sigma (gg\rightarrow ZH)$, $\sigma {(bbH)}$, $\sigma (ttH)$, and $\sigma (tH)$ whose SM cross sections are listed in Table 1.^6^ Table 9 summarises the Higgs boson coupling-strength characteristics of all production processes and lists the rate scaling behaviour in terms of Higgs boson coupling-strength scale factors.

**Fig. 7:**
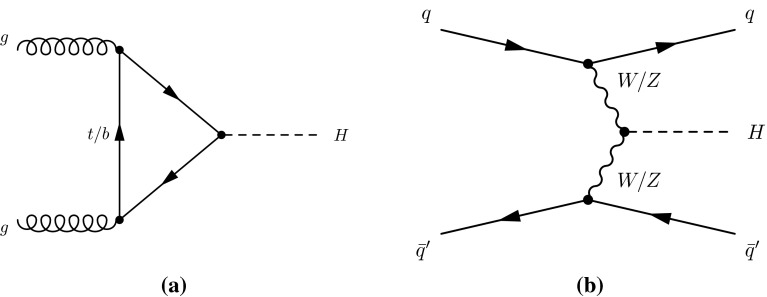
Feynman diagrams of Higgs boson production via **a** the ggF and **b** VBF production processes

The ggF production process (Fig. 7a) involves a loop process at lowest order, with contributions from *t*- and *b*-quark loops and a small interference between them. The VBF production (Fig. 7b) process probes a combination of $\kappa _{{ W}}$ and $\kappa _{{ Z}}$ coupling-strength scale factors, with a negligible amount ($\ll$0.1 %) of interference between these tree-level contributions.

**Fig. 8:**
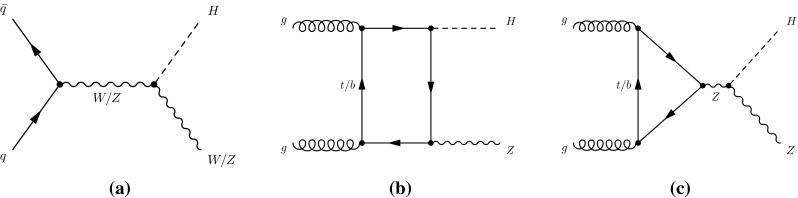
Feynman diagrams of Higgs boson production via **a** the $q\bar{q} \rightarrow VH$ and **b**, **c** $gg\rightarrow ZH$ production processes

The $q\bar{q}\rightarrow WH$ and $q\bar{q}\rightarrow ZH$ processes (Fig. 8a) each probe a single coupling strength, with scale factors $\kappa _{{ W}}$ and $\kappa _{{ Z}}$, respectively. The gluon-initiated associated production of a Higgs boson with a *Z* boson, $\sigma (gg\rightarrow ZH)$, is characterised by gluon-fusion-style production involving *t*, *b*-quark loops where the *Z* boson is always radiated from the fermion loop and the Higgs boson is either radiated directly from the fermion loop (Fig. 8b), or is radiated from the outgoing *Z* boson (Fig. 8c). The cross section of $gg\rightarrow ZH$ production is sensitive to the relative sign between $\kappa _{{ t}}$ and $\kappa _{{ Z}}$ due to interference between these contributions. This separate treatment of $gg\rightarrow ZH$ production is not present in the framework described in Ref. [32].

**Fig. 9:**
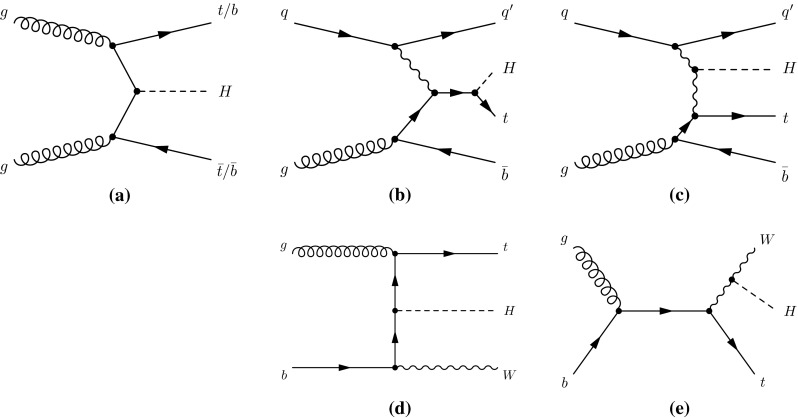
Feynman diagrams of Higgs boson production via **a** the *ttH* (*bbH*) and **b**, **c** $tHq^{\prime }b$ and **d**, **e** *WtH* processes

The *ttH* production process (Fig. 9a) directly probes the Higgs boson coupling strength to top quarks, parameterised in the framework with the scale factor $\kappa _{{ t}}$. Tree-level *tH* production, comprising the processes $qg \rightarrow tHbq^{\prime }$ (Fig. 9b, c) and $gb \rightarrow WtH$ (Fig. 9d, e), is included as background to events in all reconstructed *ttH* categories, and has for SM Higgs boson coupling strengths a large destructive interference [69] between contributions where the Higgs boson is radiated from the *W* boson and from the top quark. The SM cross section for *tH* production is consequently small, about 14 % of the *ttH* cross section. However, for negative $\kappa _{{ t}}$ the interference becomes constructive and, following Table 9, the cross section increases by a factor of 6 (13) for $|\,{\kappa _{{ t}}}\,| = |\,{\kappa _{{ W}}}\,| = 1$ for the $gb \rightarrow WtH$ ($qg \rightarrow tHbq^\prime$) process, making the *tH* process sensitive to the relative sign of the *W* and top-quark coupling strength, despite its small SM cross section. The modelling of *tH* production is not present in the framework described in Ref. [32].

The *bbH* (Fig. 9a) production process directly probes the Higgs boson coupling strength to *b*-quarks, with scale factor $\kappa _{{ b}}$. Simulation studies using *bbH* samples produced in the four-flavour scheme [82, 96] have shown that the ggF samples are a good approximation for *bbH* production for the most important analysis categories, therefore *bbH* production is always modelled using simulated ggF events (see Sect. 2.10).

**Fig. 10:**
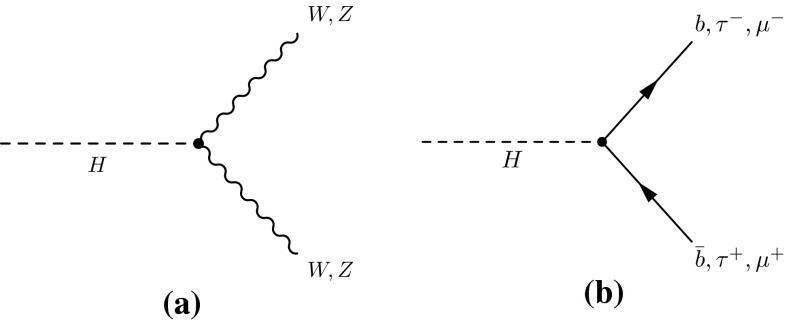
Feynman diagrams of Higgs boson decays **a** to *W* and *Z* bosons and **b** to fermions

**Fig. 11:**
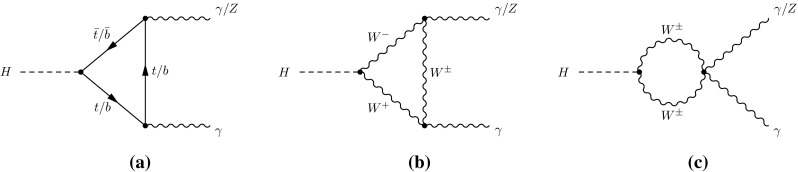
Feynman diagrams of Higgs boson decays to a pair of photons, or to a photon and a *Z* boson

The combined input channels probe seven Higgs boson decay modes. Five of these decay modes, $H\rightarrow WW^*$, $H\rightarrow ZZ^*$, $H\rightarrow b\bar{b}$, $H\rightarrow {\tau \tau }$, and $H\rightarrow \mu \mu$ each probe a single coupling-strength scale factor to either a gauge boson (Fig. 10a) or to a fermion (Fig. 10b). The remaining two decay modes, $H\rightarrow \gamma \gamma$ and $H\rightarrow Z\gamma$ are characterised by the interference between *W* boson or top-quark loop diagrams (Fig. 11). These modes probe the *W* and *t* coupling strengths as well as their relative sign through interference effects.

For completeness it should be noted also that the ggF, *tH* and $gg\rightarrow ZH$ cross sections expressed in Higgs boson coupling strengths depend on the kinematic selection criteria used. The *b*–*t* interference expression quoted in Table 9 for ggF is valid for the inclusive cross section, but in events with additional jets the top-quark loop dominates, and the observed interference is somewhat smaller. For $gg\rightarrow ZH$ production the effect of phase-space dependence was estimated for $H\rightarrow b\bar{b}$ decays with a variant of the coupling model that introduces separate coupling-dependent cross-section expressions for each of the *Z* boson $p_\mathrm{T}$ bins of the $H\rightarrow b\bar{b}$ analysis. The effect on coupling strength measurements of approximating the $gg\rightarrow ZH$ production cross section with an inclusive expression instead of using the set of $p_\mathrm{T}$-dependent expressions was determined to be negligible at the current experimental precision, with the largest effect being a $\sim 0.1\sigma$ reduction of the expected sensitivity in the determination of the relative sign of the *W* / *Z* couplings. Neither this phase-space dependence, nor that of ggF are considered in this paper. For the *tH* process on the other hand, which features a comparatively large *W*–*t* interference term, the effect of phase-space dependence is taken into account, even though Table 9 only lists the inclusive expression.

#### Effective coupling-strength scale factors

In some of the fits, effective scale factors $\kappa _{{ g}}$, $\kappa _{{\gamma }}$ and $\kappa _{{ Z}{\gamma }}$ are introduced to describe the processes $gg \rightarrow H$, $H \rightarrow \gamma \gamma$ and $H\rightarrow Z\gamma$, which are loop-induced in the SM, as shown in Figs. 7a and 11, respectively. In other fits they are treated as a function of the more fundamental coupling-strength scale factors $\kappa _{{ t}}$, $\kappa _{{ b}}$, $\kappa _{{ W}}$, and similarly for all other particles that contribute to these SM loop processes. In these cases, the loop contributions are expressed in terms of the fundamental coupling strengths, including all interference effects, as listed for the SM in Table 9. The loop process $gg\rightarrow ZH$ is never treated as an effective scale factor, as unlike in the other loop processes, a *ggHZ* contact interaction from new physics would likely show a kinematic structure very different from the SM $gg\rightarrow ZH$ process [58] assumed in the current study and is expected to be suppressed. What then remains of BSM effects on the $gg\rightarrow ZH$ process are modifications of the Higgs boson couplings to the top quark (Fig. 8b) and the *Z* boson (Fig. 8c), which are taken into account within the limitation of the framework by the coupling-strength scale factors $\kappa _{{ t}}$ and $\kappa _{{ Z}}$.

**Table 10:** Summary of benchmark coupling models considered in this paper, where $\lambda _{ij}\equiv \kappa _i/\kappa _j$, $\kappa _{ii}\equiv \kappa _i\kappa _i/\kappa _{{ H}}$, and the functional dependence assumptions are: $\kappa _{V}=\kappa _{{ W}}= \kappa _{{ Z}}$, $\kappa _F = \kappa _{{ t}} = \kappa _{{ b}} = \kappa _{{\tau }} = \kappa _{{\mu }}$ (and similarly for the other fermions), $\kappa _{{ g}} = \kappa _{{ g}}(\kappa _{{ b}}, \kappa _{{ t}})$, $\kappa _{{\gamma }}=\kappa _{{\gamma }}(\kappa _{{ b}}, \kappa _{{ t}}, \kappa _{{\tau }}, \kappa _{{ W}})$, and $\kappa _{{ H}}=\kappa _{{ H}}(\kappa _{i})$. The tick marks indicate which assumptions are made in each case. The last column shows, as an example, the relative coupling strengths involved in the $gg\rightarrow H{\rightarrow \,}\gamma \gamma$ process

Section in this paper	Corresponding table in Ref.[32]	Probed couplings	Parameters of interest	Functional assumptions					Example: $gg\rightarrow H{\rightarrow \,}\gamma \gamma$
				$\kappa _{V}$	$\kappa _{F}$	$\kappa _{{ g}}$	$\kappa _{{\gamma }}$	$\kappa _{{ H}}$	
5.2.1	43.1	Couplings to fermions and bosons	$\kappa _{V}$, $\kappa _{F}$	$\checkmark$	$\checkmark$	$\checkmark$	$\checkmark$	$\checkmark$	$\kappa _{F}^2 \cdot \kappa _{{\gamma }}^2(\kappa _{F},\kappa _{V})/ \kappa _{{ H}}^2(\kappa _{F},\kappa _{V})$
5.2.2	43.2		$\kappa _{F}$, $\kappa _{V}$, $\mathrm {BR_{i.,u.}}$	$$\begin{array}{cc} \le \! 1 \\ - \end{array}$$	$$\begin{array}{cc} -\\ - \end{array}$$	$$\begin{array}{cc} \checkmark \\ \checkmark \end{array}$$	$$\begin{array}{cc} \checkmark \\ \checkmark \end{array}$$	$$\begin{array}{ll} \checkmark \\ \kappa _\mathrm{on}=\kappa _\mathrm{off} \end{array}$$	$\frac{\kappa _{F}^2 \cdot \kappa _{{\gamma }}(\kappa _F,\kappa _V)^2}{\kappa _{{ H}}^2(\kappa _{F},\kappa _{V})} \cdot (1-\mathrm {BR_{i.,u.}})$
5.2.3	43.3		$\lambda _{FV}$, $\kappa _{VV}$	$\checkmark$	$\checkmark$	$\checkmark$	$\checkmark$	$-$	$\kappa _{VV}^2 \cdot \lambda _{FV}^2 \cdot \kappa _{{\gamma }}^2(\lambda _{FV},\lambda _{FV},\lambda _{FV},1)$
5.3.1	46	Up-/down-type fermions	$\lambda _{du}$, $\lambda _{Vu}$, $\kappa _{uu}$	$\checkmark$	$\kappa _u$, $\kappa _d$	$\checkmark$	$\checkmark$	$-$	$\kappa _{uu}^2 \cdot \kappa _{{ g}}^2(\lambda _{du},1) \cdot \kappa _{{\gamma }}^2(\lambda _{du},1,\lambda _{du},\lambda _{Vu})$
5.3.2	47	Leptons/quarks	$\lambda _{\ell q}$, $\lambda _{Vq}$, $\kappa _{qq}$	$\checkmark$	$\kappa _\ell$, $\kappa _q$	$\checkmark$	$\checkmark$	$-$	$\kappa _{qq}^2 \cdot \kappa _{{\gamma }}^2(1,1,\lambda _{\ell q},\lambda _{Vq})$
5.4.1	48.1	Vertex loops + ${ H}\!\rightarrow$invisible/undetected decays	$$\begin{array}{cc}\kappa _{{ g}}, \kappa _{{\gamma }},\\ \kappa _{{ Z}{\gamma }}\end{array}$$	=1	=1	$-$	$-$	$\checkmark$	$\kappa _{{ g}}^2 \cdot \kappa _{{\gamma }}^2/ \kappa _{{ H}}^2(\kappa _{{ g}},\kappa _{{\gamma }})$
5.4.2	48.2		$$\begin{array}{cc}\kappa _{{ g}}, \kappa _{{\gamma }}, \\ \kappa _{{ Z}{\gamma }}, \mathrm {BR_{i.,u.}} \end{array}$$	=1	=1	$-$	$-$	$\checkmark$	$\kappa _{{ g}}^2 \cdot \kappa _{{\gamma }}^2/ \kappa _{{ H}}^2(\kappa _{{ g}},\kappa _{{\gamma }}) \cdot (1-\mathrm {BR_{i.,u.}})$
5.4.3	49		$$\begin{array}{cc} \kappa _{F}, \kappa _{V}, \kappa _{{ g}}, \kappa _{{\gamma }}, \\ \kappa _{{ Z}{\gamma }}, \mathrm {BR_{i.,u.}} \end{array}$$	$$\begin{array}{cc} \le \! 1\\ -\end{array}$$	$$\begin{array}{cc} -\\ - \end{array}$$	$$\begin{array}{cc} -\\ - \end{array}$$	$$\begin{array}{cc} -\\ - \end{array}$$	$$\begin{array}{ll} \checkmark \\ \kappa _\mathrm{on}=\kappa _\mathrm{off} \end{array}$$	$\frac{\kappa _{F}^2 \cdot \kappa _{{\gamma }}(\kappa _F,\kappa _V)^2}{\kappa _{{ H}}^2(\kappa _F,\kappa _V,\kappa _{{ g}},\kappa _{{\gamma }})} \cdot (1-\mathrm {BR_{i.,u.}})$
5.5.1	51	Generic models with and without assumptions on vertex loops and $\Gamma _{{ H}}$	$\kappa _{{ W}}$, $\kappa _{{ Z}}$, $\kappa _{{ t}}$, $\kappa _{{ b}}$, $\kappa _{{\tau }}$, $\kappa _{{\mu }}$	$-$	$-$	$\checkmark$	$\checkmark$	$\checkmark$	$\frac{\kappa _{{ g}}^2(\kappa _{{ b}},\kappa _{{ t}}) \cdot \kappa _{{\gamma }}^2(\kappa _{{ b}},\kappa _{{ t}},\kappa _{{\tau }},\kappa _{{\mu }},\kappa _{{ W}})}{\kappa _{{ H}}^2(\kappa _{{ b}},\kappa _{{ t}},\kappa _{{\tau }},\kappa _{{\mu }},\kappa _{{ W}},\kappa _{{ Z}})}$
5.5.2	50.2		$$\begin{array}{cc} \kappa _{{ W}}, \kappa _{{ Z}}, \kappa _{{ t}}, \kappa _{{ b}},\\ \kappa _{{\tau }}, \kappa _{{\mu }}, \kappa _{{ g}}, \kappa _{{\gamma }}, \\ \kappa _{{ Z}{\gamma }}, \mathrm {BR_{i.,u.}} \end{array}$$	$$\begin{array}{cc} \le \! 1\\ -\\ - \end{array}$$	$$\begin{array}{ccc} -\\ -\\ -\end{array}$$	$$\begin{array}{ccc} -\\ -\\ - \end{array}$$	$$\begin{array}{ccc} -\\ -\\ - \end{array}$$	$$\begin{array}{lll} \checkmark \\ \checkmark \\ \kappa _\mathrm{on}=\kappa _\mathrm{off} \end{array}$$	$\frac{\kappa _{{ g}}^2 \cdot \kappa _{{\gamma }}^2}{\kappa _{{ H}}^2(\kappa _{{ b}},\kappa _{{ t}},\kappa _{{\tau }},\kappa _{{\mu }},\kappa _{{ W}},\kappa _{{ Z}})} \cdot (1-\mathrm {BR_{i.,u.}})$
5.5.3	50.3		$$\begin{array}{ccc} \lambda _{{ W}{ Z}}, \lambda _{{ t}{ g}}, \lambda _{{ b}{ Z}}\\ \lambda _{\tau { Z}}, \lambda _{{ g}{ Z}}, \lambda _{{\gamma }{ Z}},\\ \lambda _{({ Z}{\gamma }){ Z}}, \kappa _{{ g}{ Z}} \end{array}$$	$-$	$-$	$-$	$-$	$-$	$\kappa _{{ g}{ Z}}^2 \cdot \lambda _{{\gamma }{ Z}}^2$

**Fig. 12:**
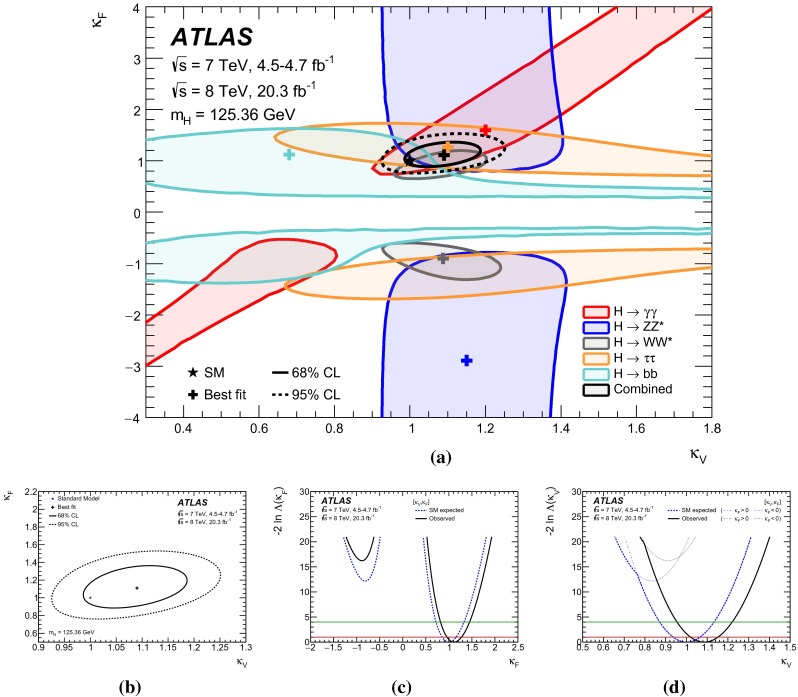
Results of fits for the two-parameter benchmark model defined in Sect. 5.2.1 that probes different coupling-strength scale factors for fermions and vector bosons, assuming only SM contributions to the total width: **a** results of the two-dimensional fit to $\kappa _{F}$ and $\kappa _{V}$, including $68~\%$ and $95~\%$ CL contours; overlaying the $68~\%$ CL contours derived from the individual channels and their combination; **b** the same measurement, without the overlays of the individual channels; **c** the profile likelihood ratio as a function of the coupling-strength scale factors $\kappa _{F}$ ($\kappa _{V}$ is profiled) and **d** as a function of  $\kappa _{V}$ ($\kappa _{F}$ is profiled). The *dashed curves* in **c** and **d** show the SM expectations. In **d** the sign of the chosen profiled solution for $\kappa _{F}$ changes at $\kappa _{V} \approx 0.8$ , causing a kink in the likelihood. The profile likelihood curves restricting $\kappa _{F}$ to be either positive or negative are also shown to illustrate that this sign change in the unrestricted profile likelihood is the origin of the kink. The *red* (*green*) *horizontal line* indicates the value of the profile likelihood ratio corresponding to a 68 % (95 %) confidence interval for the parameter of interest, assuming the asymptotic $\chi ^2$ distribution for the test statistic

#### Strategies for measurements of absolute coupling strengths

As all observed Higgs boson cross sections in the LO framework are inversely proportional to the Higgs boson width (Eq. (7)), which is not experimentally constrained to a meaningful precision at the LHC, only ratios of coupling strengths can be measured at the LHC without assumptions about the Higgs boson width. To make measurements of absolute coupling strengths, an assumption about the Higgs boson width must be introduced.

The simplest assumption is that there are no invisible or undetected Higgs boson decays, i.e. $\mathrm {BR_{i.,u.}} =0$ is assumed in Eq. (8). An alternative, less strong assumption, is that $\kappa _{{ W}} \le 1$ and $\kappa _{{ Z}} \le 1$[32]. This assumption is theoretically motivated by the premise that the Higgs boson should solve the unitarity problem in vector boson scattering and also holds in a wide class of BSM models. In particular, it is valid in any model with an arbitrary number of Higgs doublets, with and without additional Higgs singlets. The assumption is also justified in certain classes of composite Higgs boson models. A second alternative is to assume that the coupling strengths in off-shell Higgs boson production are identical to those for on-shell Higgs boson production. Under the assumption that the off-shell signal strength and coupling-strength scale factors are independent of the energy scale of Higgs boson production, the total Higgs boson decay width can be determined from the ratio of off-shell to on-shell signal strengths [21]. The constraint $\mathrm {BR_{i.,u.}} \ge 0$, motivated by the basic assumption that the total width of the Higgs boson must be greater or equal to the sum of the measured partial widths, always introduces a lower bound on the Higgs boson width. The difference in effect of these assumptions is therefore mostly in the resulting upper limit on the Higgs boson width. The assumptions made for the various measurements are summarised in Table 10 and discussed in the next sections together with the results.

### Fermion versus vector (gauge) coupling strengths

Benchmark coupling models in this section allow for different Higgs boson coupling strengths to fermions and bosons, reflecting the different structure of the interactions of the SM Higgs sector with gauge bosons and fermions. It is always assumed that only SM particles contribute to the $gg\rightarrow H$, $H{\rightarrow \,}\gamma \gamma$, $H\rightarrow Z\gamma$ and $gg\rightarrow ZH$ vertex loops, and modifications of the coupling-strength scale factors for fermions and vector bosons are propagated through the loop calculations. Models with and without assumptions about the total width are presented.

#### Assuming only SM contributions to the total width

In the first benchmark model no undetected or invisible Higgs boson decays are assumed to exist, i.e. $\mathrm {BR_{i.,u.}} =0$. The universal coupling-strength scale factors $\kappa _F$ for all fermions and $\kappa _V$ for all vector bosons are defined in this model as:

$$\begin{aligned} \kappa _V= & {} \kappa _{{ W}} = \kappa _{{ Z}} \\ \kappa _F= & {} \kappa _{{ t}} = \kappa _{{ b}} = \kappa _{{\tau }} = \kappa _{{ g}} = \kappa _{{\mu }} . \end{aligned}$$

As only SM particles are assumed to contribute to the $gg\rightarrow H$ loop in this benchmark model, the gluon fusion process depends directly on the fermion scale factor $\kappa _F^2$. Only the relative sign between $\kappa _F$ and $\kappa _V$ is physical and hence in the following only $\kappa _V>0$ is considered, without loss of generality. Sensitivity to this relative sign is gained from the negative interference between the loop contributions of the *W* boson and the *t*-quark in $H{\rightarrow \,}\gamma \gamma$ and $H\rightarrow Z\gamma$ decays and in $gg\rightarrow ZH$ production, as well as from the *tH* processes (see the corresponding expressions in Table 9).

Figure 12 shows the results of the fits for this benchmark model. Figure 12a illustrates how the decays $H{\rightarrow \,}\gamma \gamma$, $H \rightarrow ZZ^{*}$, $H \rightarrow WW^{*}$, $H \rightarrow \tau \tau$ and $H \rightarrow b\bar{b}$ contribute to the combined measurement. The slight asymmetry in $\kappa _{F}$ for $H \rightarrow WW^{*}$ and $H \rightarrow b\bar{b}$ decays is introduced by the small contributions of the *tH* and $gg\rightarrow ZH$ production processes that contribute to these decay modes, and which are sensitive to the sign of $\kappa _{F}$ due to interference effects. The strong constraint on $\kappa _F$ from $H \rightarrow WW^{*}$ decays is related to the $3.2\sigma$ observation of the VBF production process in this channel [11]. Outside the range shown in Fig. 12a there are two additional minima for $H{\rightarrow \,}\gamma \gamma$. The long tails in the $H \rightarrow b\bar{b}$ contour towards high values of $|\kappa _{V}|$ are the result of an asymptotically disappearing sensitivity of the observed signal strength in the $b\bar{b}$ final states to $\kappa _{V}$ at large values of $\kappa _{V}$. The combined measurement without overlays is also shown in Fig. 12b.

Figure 12a, b only show the SM-like minimum with a positive relative sign, as the local minimum with negative relative sign is disfavoured at the $4.0\sigma$ level, which can been seen in the wider scan of $\kappa _{F}$, where $\kappa _V$ is profiled, shown in Fig. 12c. The likelihood as a function of $\kappa _V$, profiling $\kappa _F$, is given in Fig. 12d. Around $\kappa _{V}=0.8$ the sign of the chosen profiled solution for $\kappa _{F}$ changes, causing a kink in the likelihood. The profile likelihood curves restricting $\kappa _{F}$ to either positive or negative values are also shown in Fig. 12d as thin curves, and illustrate that this sign change in the unrestricted profile likelihood is the origin of the kink.

Both $\kappa _{F}$ and $\kappa _{V}$ are measured to be compatible with their SM expectation and the two-dimensional compatibility of the SM hypothesis with the best-fit point is $41~\%$. The best-fit values and uncertainties are:

$$\begin{aligned} \kappa _V= & {} 1.09\pm 0.07 \left[ ^{+0.05}_{-0.05}(\mathrm{stat.})\,^{+0.03}_{-0.03}(\mathrm{syst.})\,^{+0.04}_{-0.03}(\mathrm{theo.})\right] \\ \kappa _F= & {} 1.11\pm 0.16 \left[ ^{+0.12}_{-0.11}(\mathrm{stat.})\,^{+0.10}_{-0.09}(\mathrm{syst.})\,^{+0.06}_{-0.05}(\mathrm{theo.})\right] . \end{aligned}$$

#### Allowing for invisible or undetected Higgs boson decays in the total width

The second benchmark model of this section allows for the presence of invisible or undetected Higgs boson decays by introducing $\mathrm {BR_{i.,u.}}$ as a free parameter in the expression of Eq. (8) for the Higgs boson total width. The free parameters of this model thus are $\kappa _F$, $\kappa _V$ and $\mathrm {BR_{i.,u.}}$. Loop processes are still assumed to have only SM content.

With the introduction of $\mathrm {BR_{i.,u.}}$ as a free parameter, the assumed Higgs boson width has no intrinsic upper bound and an additional constraint must be imposed on the model that infers an upper bound on $\Gamma _{{ H}}$. Both choices of constraints on the total width discussed in Sect. 5.1 are studied: $\kappa _{V}<1$ and $\kappa _\mathrm{on}=\kappa _\mathrm{off}$.

**Fig. 13:**
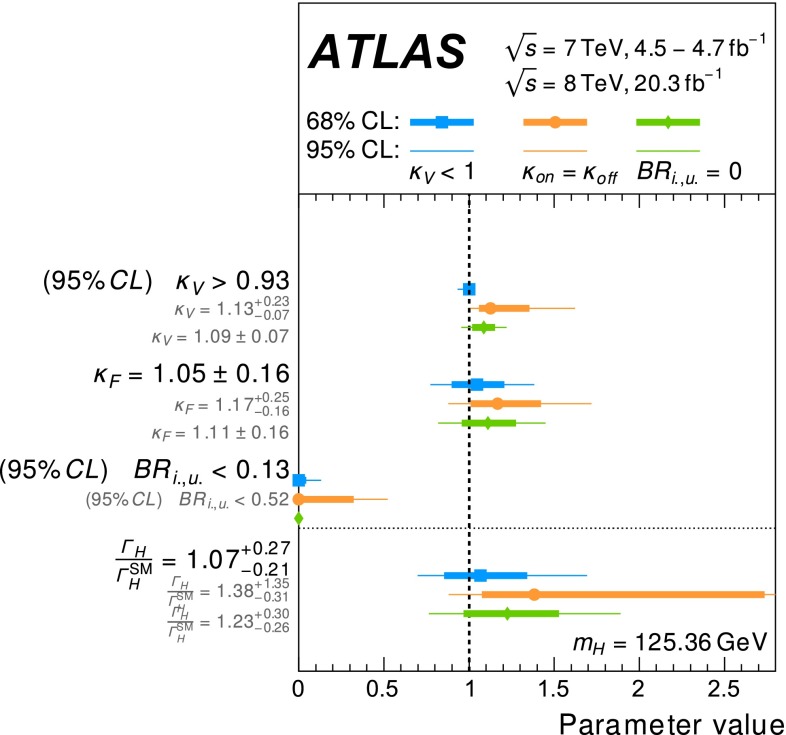
Results of fits for benchmark models that probe for potential extra contributions to the total width, but do not allow contributions from non-SM particles in the $H{\rightarrow \,}\gamma \gamma$, $gg\rightarrow H$ and $H \rightarrow Z \gamma$ loops, with free gauge and fermion coupling-strength scale factors $\kappa _V,\kappa _F$. The estimated values of each parameter under the constraint $\kappa _{V}<1$, $\kappa _\mathrm{on}=\kappa _\mathrm{off}$ or $\mathrm {BR_{i.,u.}} =0$ are shown with markers in the shape of *box*, *circle*, or *diamond*, respectively. The *inner* and *outer bars* correspond to 68 and 95 % CL intervals. The confidence intervals of $\mathrm {BR_{i.,u.}}$ and, in the benchmark model with the constraint $\kappa _{V}<1$, also $\kappa _{V}$, are estimated with respect to their physical bounds, as described in the text. The numerical values of the fit under the constraint $\kappa _{V}<1$ are shown on the *left*. Values for the two alternative constraints are also shown (in a reduced font size due to space constraints)

Figure 13 shows the results of fits for this benchmark scenario. For comparison the results of the benchmark model of Sect. 5.2.1 are included, corresponding to the condition $$\mathrm {BR_{i.,u.}} =0$$. The coupling-strength scale factors $$\kappa _{F}$$ and $$\kappa _{V}$$ are measured to be compatible with the SM values and a limit is set on the fraction of Higgs boson decays to invisible or undetected final states. The three-dimensional compatibility of the SM hypothesis with the best-fit point is $$99~\%$$$$(29~\%)$$, when applying the $$\kappa _V<1$$ (off-shell) constraint, respectively. When imposing the physical constraint $$\mathrm {BR_{i.,u.}} \ge 0$$, the $$95~\%$$ CL upper limit is $$\mathrm {BR_{i.,u.}} < 0.13$$ ($$0.52$$), when applying the constraint $$\kappa _V<1$$ ($$\kappa _\mathrm{on}=\kappa _\mathrm{off}$$). The corresponding expected limit on $$\mathrm {BR_{i.,u.}}$$, under the hypothesis of the SM, is $$0.24$$ ($$0.71$$).

**Fig. 14:**
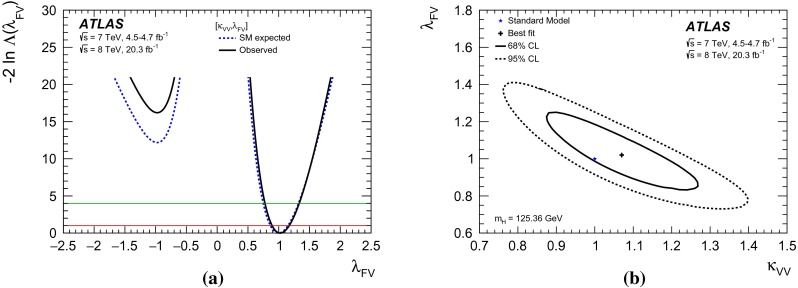
Results of fits for the two-parameter benchmark model defined in Sect. 5.2.3 that probes different coupling-strength scale factors for fermions and vector bosons without assumptions about the total width: **a** profile likelihood ratio as a function of the coupling-strength scale factor ratio $\lambda _{FV}$ ($\kappa _{VV}$ is profiled). The *dashed curve* shows the SM expectation. The *red* (*green*) *horizontal line* indicates the value of the profile likelihood ratio corresponding to a 68 % (95 %) confidence interval for the parameter of interest, assuming the asymptotic $\chi ^2$ distribution for the test statistic. **b** Results of the two-dimensional fit to $\kappa _{VV}$ and $\lambda _{FV}$, including 68 and 95 % CL contours

Also shown in Fig. 13 is the uncertainty on the total width that the model variants allow, expressed as the ratio $\Gamma _{{ H}}/\Gamma _{{ H}}^\mathrm{SM}$. These estimates for the width are obtained from alternative parameterisations of these benchmark models, where the coupling-strength scale factor $\kappa _{F}$ is replaced by the expression that results from solving Eq. (8) for $\kappa _{F}$, introducing $\Gamma _{{ H}}/\Gamma _{{ H}}^\mathrm{SM}$ as a parameter of the model. Figure 13 shows that the upper bound on the Higgs boson width from the assumption $\kappa _\mathrm{off}=\kappa _\mathrm{on}$ is substantially weaker than the bound from the assumption $\kappa _{V}<1$. These choices of constraints on the Higgs boson width complement each other in terms of explored parameter space: the present limit of $\mu _\mathrm{off}<5.1$ [21] in the combined off-shell measurement in the $H \rightarrow WW^{*}$ and $H \rightarrow ZZ^{*}$ channels effectively constrains $\kappa _{V}$ to be greater than one in the combined fit when exploiting the assumption $\kappa _\mathrm{on}=\kappa _\mathrm{off}$.

The parameterisation of the off-shell signal strength $\mu _\mathrm{off}$ in terms of couplings implicitly requires that $\mu _\mathrm{off}\ge 0$ (see Ref. [21] for details). This boundary condition causes the distribution of the test statistic to deviate from its asymptotic form for low values of $\sigma _\mathrm{off}$, with deviations in *p*-values of up to 10 % for $\sigma _\mathrm{off} \approx 2.5$, which corresponds to the value of $\sigma _\mathrm{off}$ at the upper boundary of the 68 % asymptotic confidence interval of $\Gamma _{{ H}}/\Gamma _{{ H}}^\mathrm{SM}$. The upper bound of the 68 % CL interval for the scenario $\kappa _\mathrm{off}=\kappa _\mathrm{on}$ shown in Fig. 13 should therefore be considered to be only approximate. Since the lower bound on $\Gamma _{{ H}}/\Gamma _{{ H}}^\mathrm{SM}$ is always dominated by the constraint $\mathrm {BR_{i.,u.}} \ge 0$, it is not affected by this deviation from the asymptotic behaviour.

#### No assumption about the total width

In the last benchmark model of this section no assumption about the total width is made. In this model only ratios of coupling-strength scale factors are measured, choosing as free parameters

$$\begin{aligned} \lambda _{FV}= & {} \kappa _F / \kappa _V \\ \kappa _{VV}= & {} \kappa _V\cdot \kappa _V / \kappa _{{ H}} , \end{aligned}$$

where $\lambda _{FV}$ is the ratio of the fermion and vector boson coupling-strength scale factors, $\kappa _{VV}$ is an overall scale that includes the total width and applies to all rates, and $\kappa _{{ H}}$ is defined in Table 9.

Figure 14 shows the results of this fit. Both ratio parameters are found to be consistent with the SM expectation and the two-dimensional compatibility of the SM hypothesis with the best-fit point is $41~\%$. The best-fit values and uncertainties, when profiling the other parameter, are:

$$\begin{aligned} \lambda _{FV}= & {} 1.02^{+0.15}_{-0.13} \left[ ^{+0.11}_{-0.11} (\mathrm{stat.})\,^{+0.08}_{-0.07}(\mathrm{syst.})\,^{+0.04}_{-0.03}(\mathrm{theo.})\right] \\ \kappa _{VV}= & {} 1.07^{+0.14}_{-0.13} \left[ ^{+0.11}_{-0.11} (\mathrm{stat.})\,^{+0.06}_{-0.06}(\mathrm{syst.})\,^{+0.04}_{-0.04}(\mathrm{theo.})\right] . \end{aligned}$$

Similar to the model described in Sect. 5.2.1, Fig. 14a shows the determination of the sign of $\lambda _{FV}$ disfavouring $\lambda _{FV}=-1$ at approximately $4.0\sigma$, while Fig. 14b shows the two-dimensional likelihood contour. The estimates of the two parameters are anticorrelated because only their product appears in the model.

### Probing relations within the fermion coupling sector

The previous sections assumed universal coupling-strength scale factors for all fermions, while many extensions of the SM predict deviations from universality within the fermion sector  [32]. In this section, benchmark models are explored that probe the relations between the up- and down-type fermions and between the lepton and quark sectors, using the information in the currently accessible channels, in particular in $H \rightarrow b\bar{b}$, $H \rightarrow \tau \tau$ and $H \rightarrow \mu \mu$ decays and *ttH* production. The models considered assume that only SM particles contribute to the $gg\rightarrow H$, $H{\rightarrow \,}\gamma \gamma$, $H\rightarrow Z\gamma$ and $gg\rightarrow ZH$ vertex loops, and modifications of the coupling-strength scale factors are propagated through the loop calculations. As only ratios of coupling-strength scale factors are explored, no assumptions on the total width are made.

**Fig. 15:**
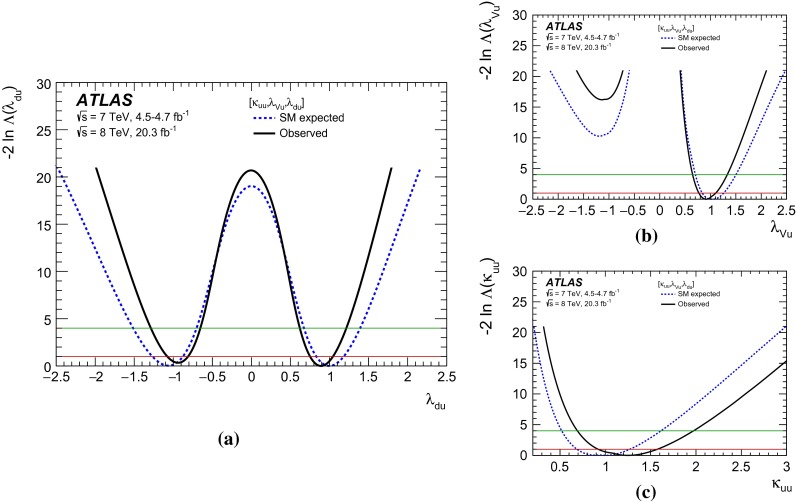
Results of fits for the benchmark model described in Sect. 5.3.1 that probes the ratio of scale factors between down- and up-type fermions: profile likelihood ratios as functions of the coupling-strength scale factor ratios **a** $\lambda _{du}$ ($\lambda _{Vu}$ and $\kappa _{uu}$ are profiled), **b** $\lambda _{Vu}$ ($\lambda _{du}$ and $\kappa _{uu}$ are profiled), and **c** the overall scale factor $\kappa _{uu}$ ($\lambda _{du}$ and $\lambda _{Vu}$ are profiled). The *dashed curves* show the SM expectations. The *red* (*green*) *horizontal line* indicates the value on the profile likelihood ratio corresponding to a 68 % (95 %) confidence interval for the parameter of interest, assuming the asymptotic $\chi ^2$ distribution for the test statistic

#### Probing the up- and down-type fermion symmetry

Many extensions of the SM contain different coupling strengths of the Higgs boson to up-type and down-type fermions. This is for instance the case for certain Two-Higgs-Doublet Models (2HDM) [117–119]. In this benchmark model the ratio $\lambda _{du}$ of down- and up-type fermions coupling-strength scale factors is probed, while vector boson coupling-strength scale factors are assumed to be unified and equal to $\kappa _{V}$. The indices *u*, *d* stand for all up- and down-type fermions, respectively. The free parameters are:

$$\begin{aligned} \lambda _{du}= & {} \kappa _{d} / \kappa _{u} \\ \lambda _{Vu}= & {} \kappa _{V} / \kappa _{u} \\ \kappa _{uu}= & {} \kappa _{u} \cdot \kappa _{u} / \kappa _{{ H}} . \end{aligned}$$

The up-type quark coupling-strength scale factor is mostly indirectly constrained through the $gg\rightarrow H$ production channel, from the Higgs boson to top-quark coupling strength, with an additional weak direct constraint from the $q\bar{q}/gg \rightarrow t\bar{t}H$ production channel, while the down-type coupling strength is constrained through the $H \rightarrow b\bar{b}$, $H \rightarrow \tau \tau$ and $H \rightarrow \mu \mu$ decays as well as weakly through the $b\bar{b}\rightarrow H$ production mode and the *b*-quark loop in the $gg\rightarrow H$ production mode.

**Fig. 16:**
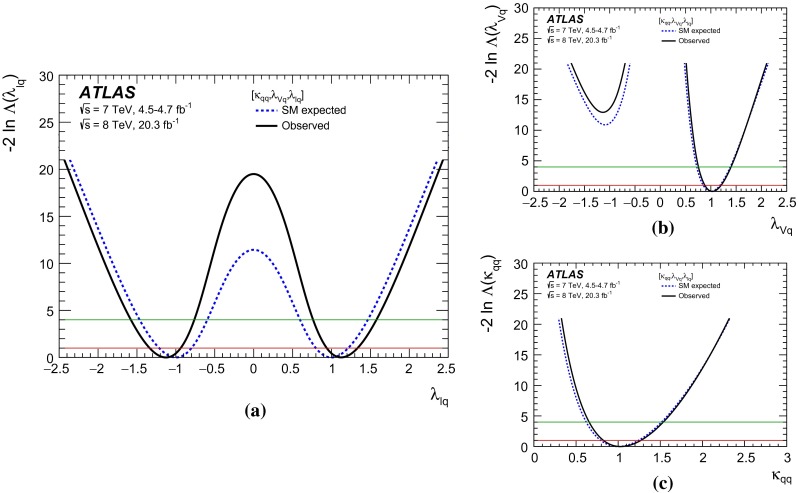
Results of fits for the benchmark model described in Sect. 5.3.2 that probes the symmetry between quarks and leptons: profile likelihood ratios as functions of the coupling-strength scale factor ratios **a** $\lambda _{\ell q}$ ($\lambda _{Vq}$ and $\kappa _{qq}$ are profiled), **b** $\lambda _{Vq}$ ($\lambda _{\ell q}$ and $\kappa _{qq}$ are profiled), and **c** the overall scale factor $\kappa _{qq}$ ($\lambda _{\ell q}$ and $\lambda _{Vq}$ are profiled). The *dashed curves* show the SM expectations. The *red* (*green*) *horizontal line* indicates the value of the profile likelihood ratio corresponding to a 68 % (95 %) confidence interval for the parameter of interest, assuming the asymptotic $\chi ^2$ distribution for the test statistic

The fit results for the parameters of interest in this benchmark model, when profiling the other parameters, are:

$$\begin{aligned} \lambda _{du}\in & {} [-1.08,-0.81] \cup [0.75,1.04]~(68~\%~ \mathrm {CL}) \\ \lambda _{Vu}= & {} 0.92^{+0.18}_{-0.16}\\ \kappa _{uu}= & {} 1.25^{+0.33}_{-0.33}. \end{aligned}$$

Near the SM prediction of $\lambda _{du}=1$, the best-fit value is $\lambda _{du} =0.90^{+0.14}_{-0.15}$. All parameters are measured to be consistent with their SM expectation and the three-dimensional compatibility of the SM hypothesis with the best-fit point is $51~\%$.

The likelihood curves corresponding to these measurements are shown in Fig. 15. The likelihood curve of Fig. 15a is nearly symmetric around $$\lambda _{du}=0$$ as the model is almost insensitive to the relative sign of $$\kappa _u$$ and $$\kappa _d$$. The interference of contributions from the *b*-quark and *t*-quark loops in the $$gg\rightarrow H$$ production induces an observed asymmetry of about $$0.6\sigma $$ (no significant asymmetry is expected with the present sensitivity). The profile likelihood ratio value at $$\lambda _{du}=0$$ provides $$4.5\sigma $$ evidence of the coupling of the Higgs boson to down-type fermions, mostly coming from the $$H \rightarrow \tau \tau $$ measurement and to a lesser extent from the $$H \rightarrow b\bar{b}$$ measurement. Vanishing coupling strengths of the Higgs boson to up-type fermions ($$\kappa _{uu} = 0$$) and vector bosons ($$\lambda _{Vu} = 0$$) are excluded at a level of $$>$$$$5\sigma $$.

#### Probing the quark and lepton symmetry

Extensions of the SM can also contain different coupling strengths of the Higgs boson to leptons and quarks, notably some variants of Two-Higgs-Doublet Models. In this benchmark model the ratio $\lambda _{\ell q}$ of coupling-strength scale factors to leptons and quarks is probed, while vector boson coupling-strength scale factors are assumed to be unified and equal to $\kappa _{V}$. The indices $\ell$, *q* stand for all leptons and quarks, respectively. The free parameters are:

$$\begin{aligned} \lambda _{\ell q}= & {} \kappa _{\ell } / \kappa _{q} \\ \lambda _{Vq}= & {} \kappa _{V} / \kappa _{q} \\ \kappa _{qq}= & {} \kappa _{q} \cdot \kappa _{q} / \kappa _{{ H}} . \end{aligned}$$

The lepton coupling strength is constrained through the $H \rightarrow \tau \tau$ and $H \rightarrow \mu \mu$ decays. The fit results for the parameters of interest of this benchmark model, when profiling the other parameters, are:

$$\begin{aligned} \lambda _{\ell q}\in & {} [-1.34, -0.94] \cup [0.94, 1.34]~(68~\%~ \mathrm {CL})\\ \lambda _{Vq}= & {} 1.03^{+0.18}_{-0.15}\\ \kappa _{qq}= & {} 1.03^{+0.24}_{-0.20}. \end{aligned}$$

Near the SM prediction of $\lambda _{\ell q}=1$, the best-fit value is $\lambda _{\ell q} = 1.12^{+0.22}_{-0.18}$. All parameters are measured to be consistent with their SM expectation and the three-dimensional compatibility of the SM hypothesis with the best-fit point is $53~\%$.

Figure 16 shows the likelihood curves corresponding to the fit results for this benchmark. Similar to the model of Sect. 5.3.1, the likelihood curve is nearly symmetric around $$\lambda _{\ell q}=0$$. A vanishing coupling strength of the Higgs boson to leptons, i.e. $$\lambda _{\ell q}=0$$, is excluded at the $$\sim \! 4.4\sigma $$ level due to the $$H \rightarrow \tau \tau $$ measurement. The profile likelihood ratio values at $$\kappa _{qq}=0$$ and $$\lambda _{Vq}=0$$ provide strong confirmation of Higgs boson couplings to quarks and vector bosons with both significances of $$>$$$$5\sigma $$.

### Probing beyond the SM contributions in loops and decays

In this section, contributions from new particles either in loops or in new final states are probed. For the $H{\rightarrow \,}\gamma \gamma$, $H \rightarrow Z \gamma$ and $gg\rightarrow H$ vertices, effective scale factors $\kappa _{{\gamma }}$, $\kappa _{{ Z}{\gamma }}$ and $\kappa _{{ g}}$ are introduced that allow for extra contributions from new particles. These effective scale factors are defined to be positive as there is by construction no sensitivity to the sign of these coupling strengths. The potential new particles contributing to these vertex loops may or may not contribute to the total width of the observed state through direct invisible or undetected decays. In the latter case the total width is parameterised in terms of the additional branching ratio $\mathrm {BR_{i.,u.}}$ into invisible or undetected particles.

#### Probing BSM contributions in loop vertices only

In the first benchmark model of this section, BSM contributions can modify the loop coupling strengths from their SM prediction, but it is assumed that there are no extra contributions to the total width caused by non-SM particles. Furthermore, all coupling-strength scale factors of known SM particles are assumed to be as predicted by the SM, i.e. $\kappa _{{ W}} = \kappa _{{ Z}} = \kappa _{{ t}} = \kappa _{{ b}} = \kappa _{{\tau }} = \kappa _{{\mu }} = 1$. The free parameters are thus $\kappa _{{ g}}$, $\kappa _{{\gamma }}$ and $\kappa _{{ Z}{\gamma }}$.

**Fig. 17:**
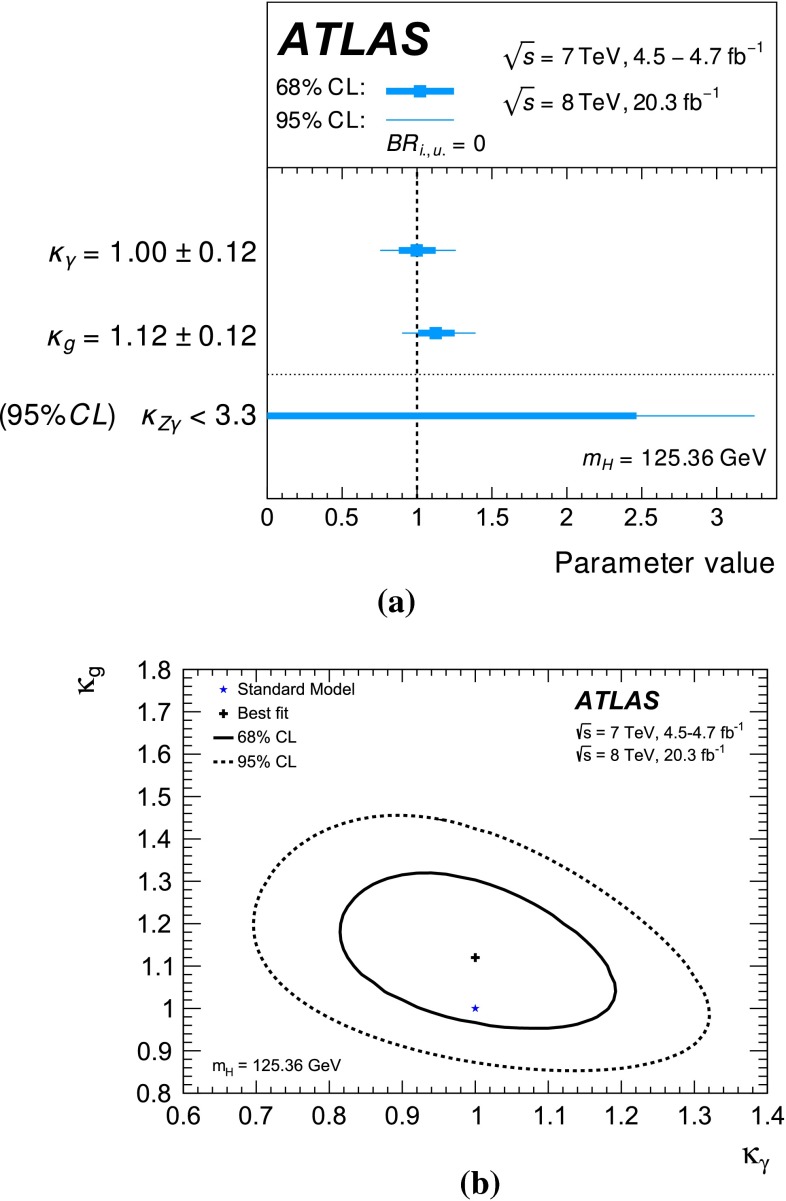
Results of fits for the benchmark model that probes for contributions from non-SM particles in the $H{\rightarrow \,}\gamma \gamma$, $H \rightarrow Z \gamma$ and $gg\rightarrow H$ loops, assuming no extra contributions to the total width: **a** overview of fitted parameters, where the *inner* and *outer bars* correspond to 68 and 95 % CL intervals, and **b** results of the two-dimensional fit to $\kappa _{{\gamma }}$ and $\kappa _{{ g}}$, including 68 and $95~\%$ CL contours ($\kappa _{{ Z}{\gamma }}$ is profiled)

Figure 17a shows the results of fits for this benchmark scenario and the best-fit values and uncertainties, when profiling the other parameters. The effective coupling-strength scale factors $\kappa _{{ g}}$ and $\kappa _{{\gamma }}$ are measured to be consistent with the SM expectation, whereas a limit is set on the effective coupling-strength scale factor $\kappa _{{ Z}{\gamma }}$. Figure 17b shows the two-dimensional likelihood contour for $\kappa _{{ g}}$ vs. $\kappa _{{\gamma }}$, where $\kappa _{{ Z}{\gamma }}$ is profiled. The three-dimensional compatibility of the SM hypothesis with the best-fit point is $69~\%$.

#### Probing BSM contributions in loop vertices and to the total width

The second benchmark model of this section removes the assumption of no invisible or undetected Higgs boson decays, introducing $\mathrm {BR_{i.,u.}}$ as additional model parameter. The free parameters of this benchmark model are thus $\kappa _{{ g}}$, $\kappa _{{\gamma }}$, $\kappa _{{ Z}{\gamma }}$ and $\mathrm {BR_{i.,u.}}$. The coupling-strength scale factors of known SM particles are still assumed to be at their SM values of 1. Due to this assumption, the parameterisation of Higgs boson channels that do not involve a loop process, e.g. VBF production of $H \rightarrow WW^{*}$ and associated production of $H \rightarrow b\bar{b}$, depends only on $\mathrm {BR_{i.,u.}}$ in this model, and not on $\kappa _{{ g}}$, $\kappa _{{\gamma }}$ or $\kappa _{{ Z}{\gamma }}$, and can hence constrain $\mathrm {BR_{i.,u.}}$ from the data. Thus no additional constraints, beyond those introduced in the benchmark model of Sect. 5.2.2, are necessary in this model.

**Fig. 18:**
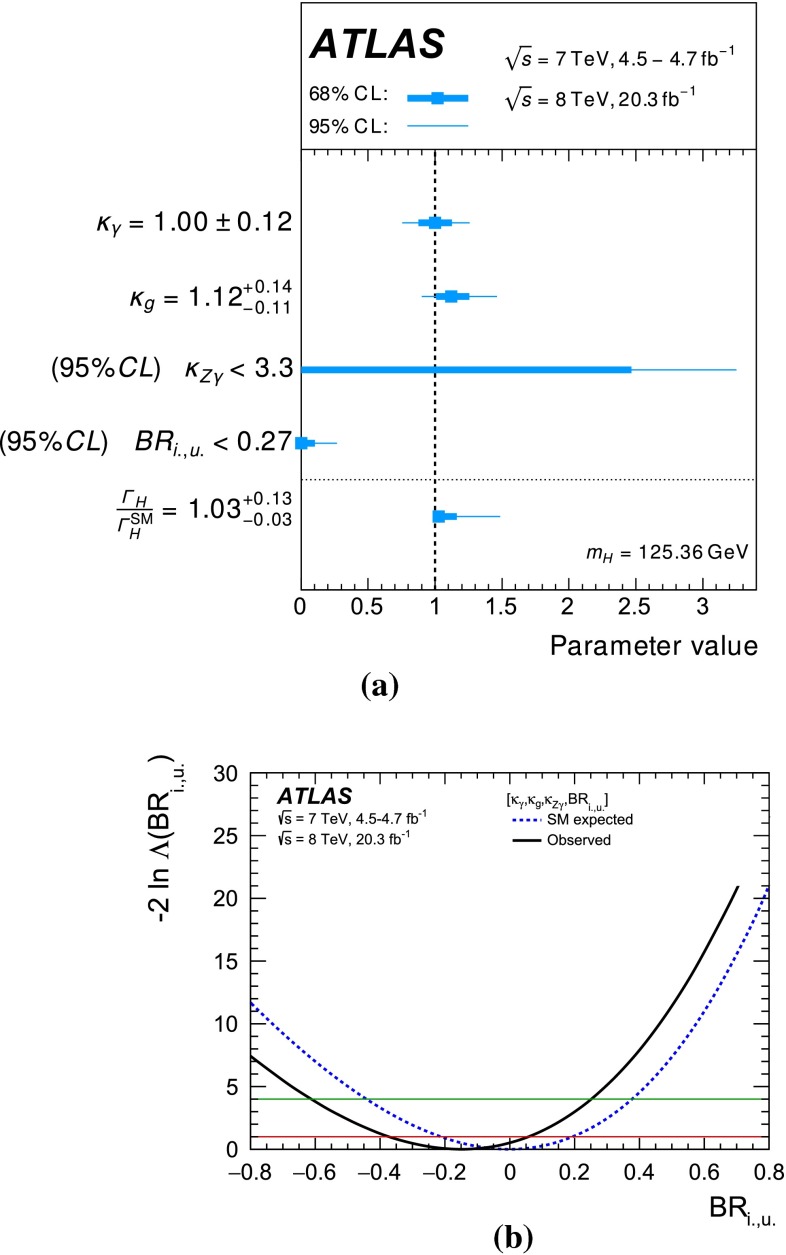
Results of fits for benchmark models that probe for contributions from non-SM particles in the $H{\rightarrow \,}\gamma \gamma$, $H \rightarrow Z \gamma$ and $gg\rightarrow H$ loops, while allowing for potential extra contributions to the total width: **a** overview of fitted parameters. The *inner* and *outer bars* correspond to 68 and 95 % CL intervals. The confidence intervals for $\mathrm {BR_{i.,u.}}$ are estimated with respect to the physical bounds as described in the text. **b** Profile likelihood ratio as a function of the branching fraction $\mathrm {BR_{i.,u.}}$ to invisible or undetected decay modes ($\kappa _{{\gamma }}$, $\kappa _{{ g}}$ and $\kappa _{{ Z}{\gamma }}$ are profiled). The *red* (*green*) *horizontal line* indicates the value of the profile likelihood ratio corresponding to a 68 % (95 %) confidence interval for the parameter of interest, assuming the asymptotic $\chi ^2$ distribution for the test statistic

**Fig. 19:**
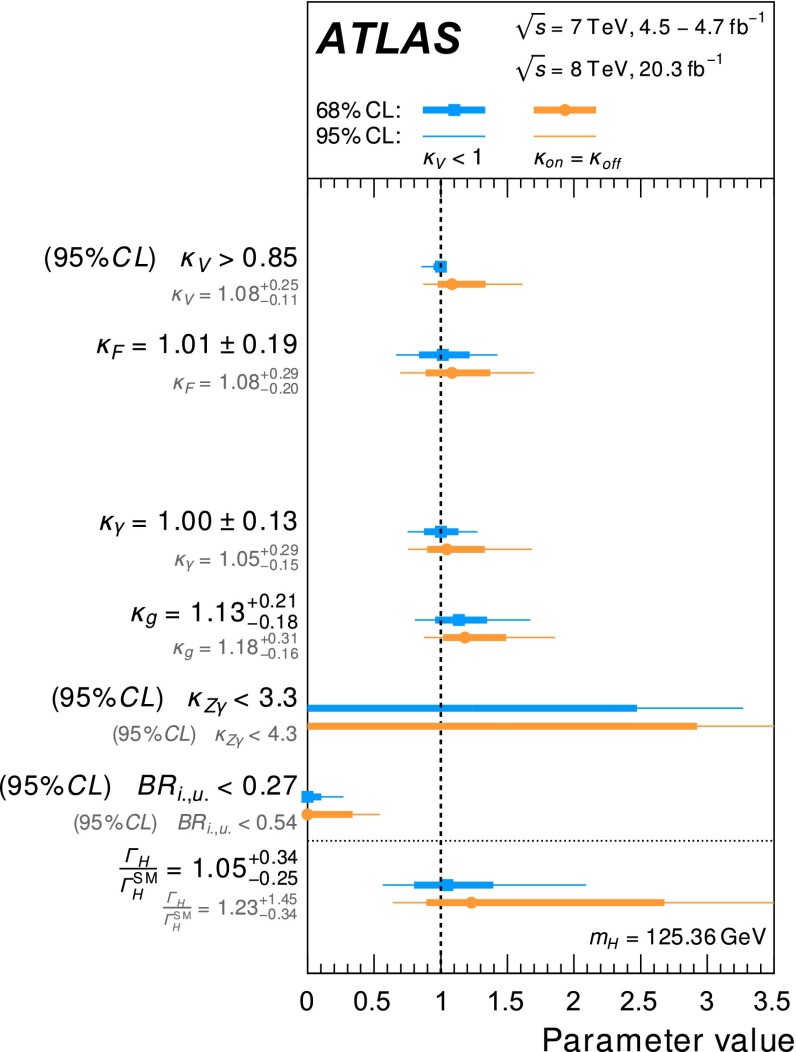
Results of fits for benchmark models that probe for contributions from non-SM particles in the $H{\rightarrow \,}\gamma \gamma$, $gg\rightarrow H$ and $H \rightarrow Z \gamma$ loops, with free gauge and fermion coupling-strength scale factors $\kappa _V,\kappa _F$, while allowing for potential extra contributions to the total width. The estimated values of each parameter under the constraint $\kappa _{V}<1$ or $\kappa _\mathrm{on}=\kappa _\mathrm{off}$ are shown with markers in the shape of a *box* or a *circle*, respectively. The *inner* and *outer bars* correspond to 68 and 95 % CL intervals. The confidence intervals of $\mathrm {BR_{i.,u.}}$ and, in the benchmark model with the constraint $\kappa _{V}<1$, also $\kappa _{V}$, are estimated with respect to their physical constraints as described in the text. The numerical values of the fit under the constraint $\kappa _{V}<1$ are shown on the *left*. Values for the alternative $\kappa _\mathrm{on}=\kappa _\mathrm{off}$ constraint are also shown (in a reduced font size due to space constraints)

**Fig. 20:**
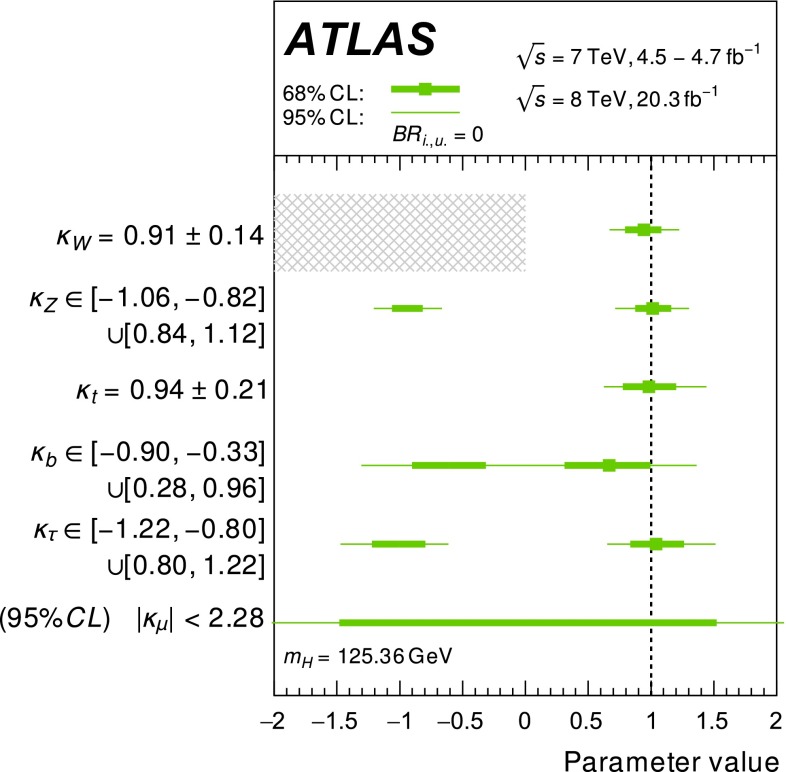
Overview of best-fit values of parameters with 68 and 95 % CL intervals for generic model 1 (see text). In this model only SM particles are considered in loops and no invisible or undetected Higgs boson decays are allowed. The sign of $\kappa _{{ W}}$ is assumed to be positive, as indicated by the *hatched area*, without loss of generality. The *inner* and *outer bars* correspond to 68 and 95 % CL intervals

The results of fits to this benchmark model are shown in Fig. 18, along with the uncertainty on the total width that this model allows, obtained in the same fashion as for the previous benchmark models. The effective coupling-strength scale factors $\kappa _{{ g}}$ and $\kappa _{{\gamma }}$ are measured to be consistent with the SM expectation, whereas limits are set on the effective coupling-strength scale factor $\kappa _{{ Z}{\gamma }}$ and the branching fraction $\mathrm {BR_{i.,u.}}$. By using the physical constraint $\mathrm {BR_{i.,u.}} >0$, the observed $95~\%$ CL upper limit is $\mathrm {BR_{i.,u.}} <0.27$ compared with the expected limit of $\mathrm {BR_{i.,u.}} <0.37$ under the SM hypothesis. The four-dimensional compatibility of the SM hypothesis with the best-fit point is $74~\%$. The best-fit values of the model parameters of interest and their uncertainties, when profiling the other parameters, are

$$\begin{aligned} \kappa _{{ g}}= & {} 1.12 ^{+0.14}_{-0.11} \left[ ^{+0.10}_{-0.08}(\mathrm{stat.})\,^{+0.05}_{-0.05}(\mathrm{syst.})\,^{+0.07}_{-0.07}(\mathrm{theo.})\right] \\ \kappa _{{\gamma }}= & {} 1.00 \pm 0.12 \left[ ^{+0.11}_{-0.11}(\mathrm{stat.})\,^{+0.05}_{-0.05}(\mathrm{syst.})\,^{+0.04}_{-0.03}(\mathrm{theo.})\right] \end{aligned}$$

In a variant of the fit where no limits are imposed on $\mathrm {BR_{i.,u.}}$ its best-fit value is

$$\begin{aligned} \mathrm {BR_{i.,u.}}= & {} -0.15 ^{+0.21}_{-0.22} \left[ ^{+0.17}_{-0.17}(\mathrm{stat.})\,^{+0.11}_{-0.11}(\mathrm{syst.})\,^{+0.06}_{-0.07}(\mathrm{theo.})\right] , \end{aligned}$$

corresponding to the likelihood curve shown in Fig. 18b. Without the condition $\mathrm {BR_{i.,u.}} \ge 0$, the best-fit value of $\mathrm {BR_{i.,u.}}$ assumes a small (unphysical) negative value that is consistent with zero within the uncertainty.

As the choice of free parameters in this model gives extra degrees of freedom to ggF production and $H{\rightarrow \,}\gamma \gamma$ and $H \rightarrow Z \gamma$ decays, the most precise measurements based on ggF production or $H{\rightarrow \,}\gamma \gamma$ decays (see Fig. 2) do not give a sizeable contribution to the determination of $\mathrm {BR_{i.,u.}}$. Instead $\mathrm {BR_{i.,u.}}$ is mostly constrained by channels sensitive to VBF and *VH* production, as the tree-level couplings involved in these production modes are fixed to their SM values within this model. The upward uncertainty on $\Gamma _{{ H}}/\Gamma _{{ H}}^\mathrm{SM}$ is notably increased with respect to that of the model in Sect. 5.4.1 due to the removing the constraint on $\mathrm {BR_{i.,u.}}$, whereas the downward uncertainty is identical due to the condition that $\mathrm {BR_{i.,u.}} \ge 0$.

#### Probing BSM contributions in loop vertices and to the total width allowing modified couplings to SM particles

The last benchmark model of this section removes the assumption of SM couplings of the Higgs boson for non-loop vertices used so far in this section, re-introducing the coupling-strength scale factors $\kappa _{F}$ and $\kappa _{V}$ defined in Sect. 5.2.1 to allow deviations of the coupling strength of the Higgs boson to fermions and gauge bosons, respectively. As the expression for $\kappa _{{ H}}$ is no longer strongly constrained due to the newly introduced degrees of freedom, the upper limit on $\Gamma _{{ H}}$ is no longer bounded, and an additional constraint on the total Higgs boson width must be introduced. Similar to the model of Sect. 5.2.2 the two choices of the constraints on the total width discussed in Sect. 5.1 are studied: $\kappa _{V}<1$ and $\kappa _\mathrm{on}=\kappa _\mathrm{off}$. The free parameters of this model are $\kappa _F$, $\kappa _V$, $\kappa _{{ g}}$, $\kappa _{{\gamma }}$, $\kappa _{{ Z}{\gamma }}$ and $\mathrm {BR_{i.,u.}}$.

**Fig. 21:**
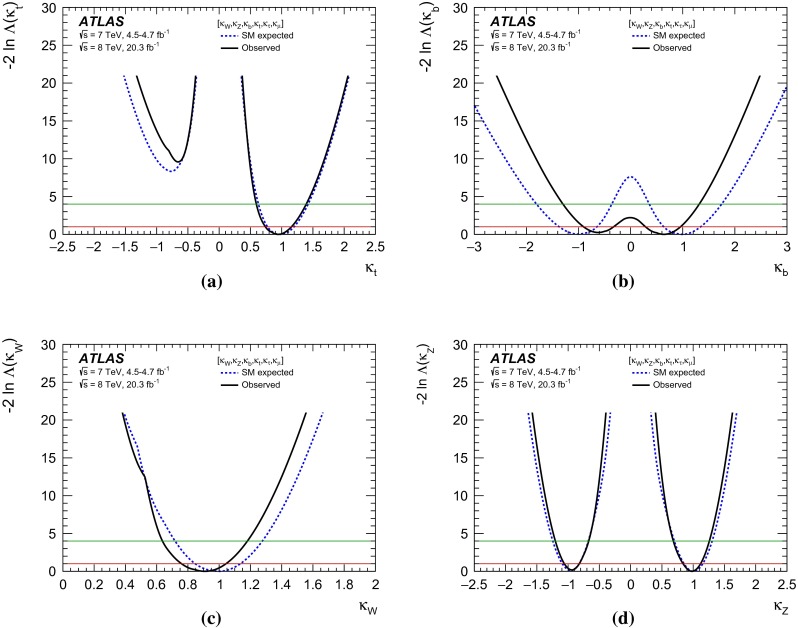
Results of fits for generic model 1 (see text): profile likelihood ratios as functions of the coupling-strength scale factors **a** $\kappa _{{ t}}$, **b** $\kappa _{{ b}}$, **c** $\kappa _{{ W}}$, and **d** $\kappa _{{ Z}}$. For each measurement, the other coupling-strength scale factors are profiled. The kinks in the curves of **a** and **c** are caused by transitions in solutions chosen by the profile likelihood for the relative sign between profiled couplings. The *dashed curves* show the SM expectations. The *red* (*green*) *horizontal line* indicates the value of the profile likelihood ratio corresponding to a 68 % (95 %) confidence interval for the parameter of interest, assuming the asymptotic $\chi ^2$ distribution for the test statistic

Figure 19 shows the best-fit values and their uncertainties. The coupling-strength scale factors $$\kappa _{{ g}}$$, $$\kappa _{{\gamma }}$$, $$\kappa _{V}$$ and $$\kappa _{F}$$ are measured to be consistent with their SM expectation, while limits are set on the coupling-strength scale factor $$\kappa _{{ Z}{\gamma }}$$ and the branching fraction $$\mathrm {BR_{i.,u.}}$$ to invisible or undetected decays. By using the physical constraint $$\mathrm {BR_{i.,u.}} \ge 0$$, the $$95~\%$$ CL upper limit is $$\mathrm {BR_{i.,u.}} < 0.27$$ ($$0.54$$) when applying the constraint $$\kappa _V<1$$ ($$\kappa _\mathrm{on}=\kappa _\mathrm{off}$$). The expected limit in case of the SM hypothesis is $$\mathrm {BR_{i.,u.}} < 0.39$$ ($$0.72$$). The six-dimensional compatibility of the SM hypothesis with the best-fit point is $$96~\%$$$$(64~\%)$$ when applying the $$\kappa _V<1$$ ($$\kappa _\mathrm{on}=\kappa _\mathrm{off}$$) constraint, respectively. The uncertainty on $$\Gamma _{{ H}}/\Gamma _{{ H}}^\mathrm{SM}$$ is significantly increased compared with models in Sects. 5.4.1 and 5.4.2 due to the further relaxed coupling constraints, in particular both the 68 and 95 % CL intervals of $$\Gamma _{{ H}}/\Gamma _{{ H}}^\mathrm{SM}$$ extend below 1.

**Fig. 22:**
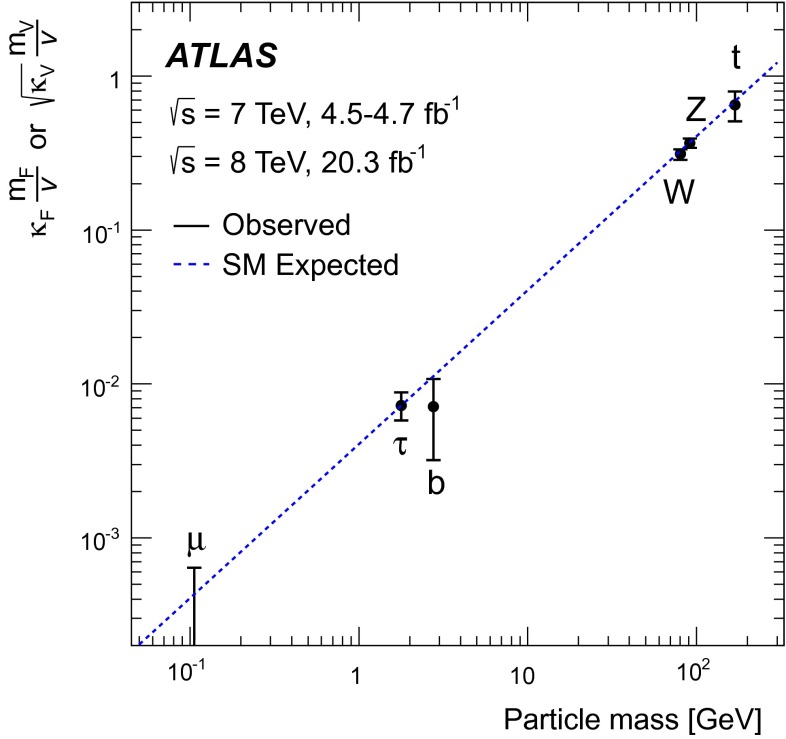
Fit results for the reduced coupling-strength scale factors $y_{V,i} = \sqrt{ \kappa _{V,i} \frac{g_{V,i}}{2v} } = \sqrt{\kappa _{V,i}}\frac{m_{V,i}}{v}$ for weak bosons and $y_{F,i} = \kappa _{F,i}\frac{g_{F,i}}{\sqrt{2}} = \kappa _{F,i}\frac{m_{F,i}}{v}$ for fermions as a function of the particle mass, assuming a SM Higgs boson with a mass of 125.36 GeV. The *dashed line* indicates the predicted mass dependence for the SM Higgs boson

**Fig. 23:**
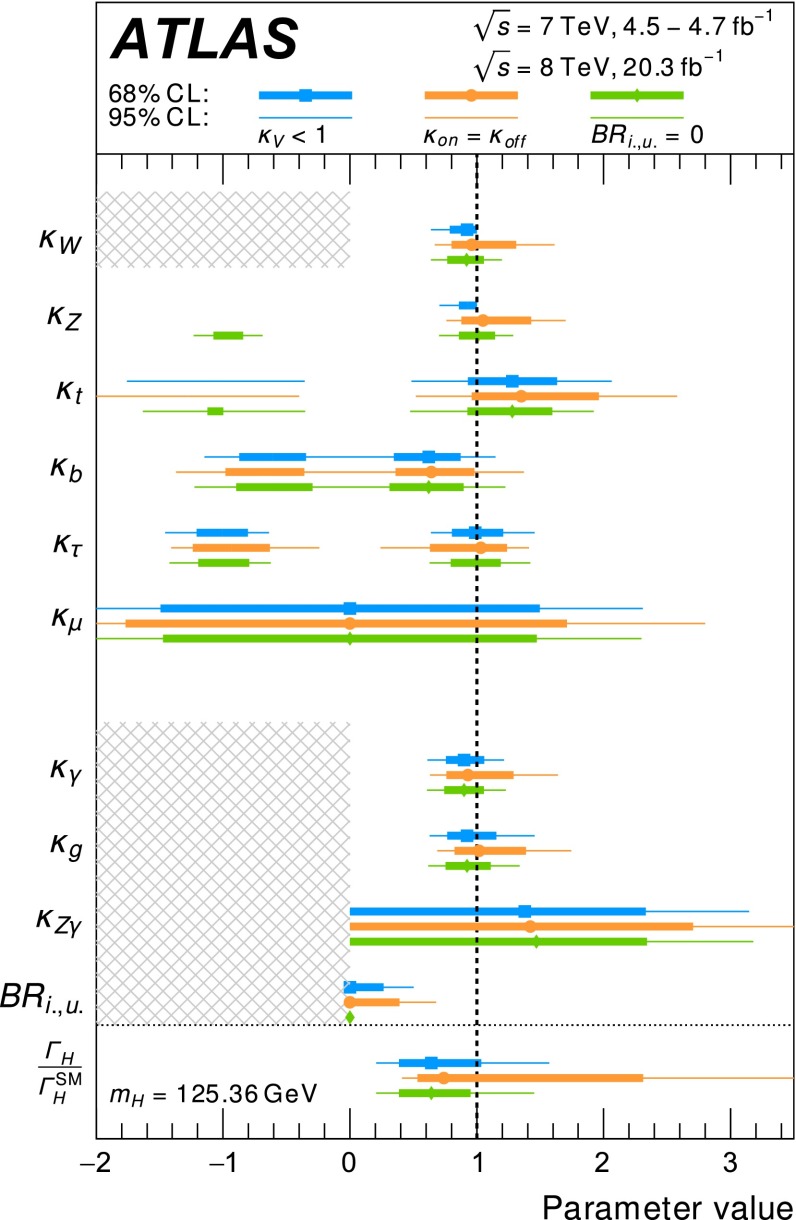
Results of fits for generic model 2 (see text): the estimated values of each parameter under the constraint $\kappa _{V}<1$, $\kappa _\mathrm{on}=\kappa _\mathrm{off}$ or $\mathrm {BR_{i.,u.}} =0$ are shown with markers in the shape of a *box*, a *circle*, or a *diamond*, respectively. The *hatched area* indicates regions that are outside the defined parameter boundaries. The *inner* and *outer bars* correspond to 68 and 95 % CL intervals. The confidence intervals of $\mathrm {BR_{i.,u.}}$ and, in the benchmark model with the constraints $\kappa _{W}<1$ and $|\kappa _{Z}|<1$, also $\kappa _{W}$ and $\kappa _{Z}$, are estimated with respect to their physical bounds as described in the text. Numerical results are shown in Table 11

### Generic models

In the benchmark models studied in Sects. 5.2, 5.3 and 5.4, specific aspects of the Higgs sector are tested by combining coupling-strength scale factors into a minimum number of parameters under certain assumptions, thereby maximising the sensitivity to the scenarios under study. In generic models the scale factors for the coupling strengths to *W*, *Z*, *t*, *b*, $\tau$ and $\mu$ are treated independently, while for the loop vertices and the total width $\Gamma _{{ H}}$, either the SM particle content is assumed (Sect. 5.5.1) or no such assumption is made (Sects. 5.5.2 and 5.5.3).

#### Generic model 1: no new particles in loops and in decays

In the first generic benchmark model all coupling-strength scale factors to SM particles, relevant to the measured modes, are fitted independently. The free parameters are: $\kappa _{{ W}}$, $\kappa _{{ Z}}$, $\kappa _{{ t}}$, $\kappa _{{ b}}$, $\kappa _{{\tau }}$, and $\kappa _{{\mu }}$. It is assumed that only SM particles contribute to Higgs boson vertices involving loops, and modifications of the coupling-strength scale factors for fermions and vector bosons are propagated through the loop calculations. No invisible or undetected Higgs boson decays are assumed to exist. Only the *W* coupling-strength scale factor is assumed to be positive without loss of generality: due to interference terms, the fit is sensitive to the relative sign of the *W* and *t* couplings (through the *tH*, $H{\rightarrow \,}\gamma \gamma$, $H \rightarrow Z \gamma$ processes) and the relative sign of the *Z* and *t* coupling (through the $gg\rightarrow ZH$ process), providing indirect sensitivity to the relative sign of the *W* and *Z* coupling. Furthermore, the model has some sensitivity to the relative sign of the *t* and *b* coupling (through the ggF process).

Figure 20 summarises the results of the fits for this benchmark scenario. All measured coupling-strength scale factors in this generic model are found to be compatible with their SM expectation, and the six-dimensional compatibility of the SM hypothesis with the best-fit point is $57~\%$. Illustrative likelihoods of the measurements summarised in Fig. 20 are shown in Fig. 21. As shown in Fig. 21a, b, the negative solution of $\kappa _{{ t}}$ is strongly disfavoured at $3.1\sigma$ ($2.9\sigma$ expected), while the negative minimum of $\kappa _{{ b}}$ is slightly disfavoured at $0.5\sigma$ (no sensitivity expected).

For the measurements in this generic model, it should be noted that the low fitted value of $\kappa _{{ b}}$ causes a reduction of the total width $\Gamma _{{ H}}$ by about 30 % compared to the SM expectation (see Table 9), which in turn induces a reduction of all other $\kappa$-values by about 20 %.

**Table 11:** Numerical results of the fits to generic model 2 : effective coupling-strength scale factors for loop processes allowing non-SM contributions with various assumptions on the total Higgs boson width. These results are illustrated in Fig. 23. The confidence interval of $\mathrm {BR_{i.,u.}}$ in the benchmark model with the constraints $\kappa _{{ W}}<1$ and $|\kappa _{{ Z}}|<1$, and the confidence intervals $\kappa _{{ W}}$ and $\kappa _{{ Z}}$, are estimated with respect to their physical bounds, as described in the text. Shown in square brackets are uncertainty components from different sources for the case of $\mathrm {BR_{i.,u.}} =0$ as an illustration. For $\kappa _{{ Z}}$ and $\kappa _{{ t}}$, the uncertainty breakdowns are provided for the preferred positive solutions. Also shown is the uncertainty on the total width that the model variants allow, expressed as the ratio $\Gamma _{{ H}}/\Gamma _{{ H}}^\mathrm{SM}$. These estimates for the width are obtained from alternative parameterisations of these benchmark models where the effective coupling-strength scale factor $\kappa _{{ g}}$ is replaced by the expression that results from solving Eq. (8) for $\kappa _{{ g}}$, introducing $\Gamma _{{ H}}/\Gamma _{{ H}}^\mathrm{SM}$ as a parameter of the model

Parameter	$\kappa _V <1$	$\kappa _\mathrm{on}=\kappa _\mathrm{off}$	$\mathrm {BR_{i.,u.}} = 0$	
			Fitted Value	Uncertainty breakdown
$\kappa _{{ W}}$	$>$0.64 (95 % CL)	$=0.96\pm ^{0.35}_{0.16}$	$=0.92^{+0.14}_{-0.15}$	$\left[ ^{+0.11}_{-0.11}(\mathrm{stat.})\,^{+0.07}_{-0.08}(\mathrm{syst.})\,^{+0.03}_{-0.03}(\mathrm{theo.})\right]$
$$\kappa _{{ Z}}$$	$$> $$0.71 (95 % CL)	$$=1.05\pm ^{0.38}_{0.17}$$	$$\in [-1.08,-0.84]\cup [0.86,1.14]$$	$$\left[ ^{+0.13}_{-0.13}(\mathrm{stat.})\,^{+0.05}_{-0.07}(\mathrm{syst.})\,^{+0.03}_{-0.02}(\mathrm{theo.})\right] $$
$\kappa _{{ t}}$	$=$ $1.28^{+0.32}_{-0.35}$	$=1.35^{+0.61}_{-0.39}$	$\in [-1.12,-1.00]\cup [0.93,1.60]$	$\left[ ^{+0.20}_{-0.22}(\mathrm{stat.})\,^{+0.22}_{-0.26}(\mathrm{syst.})\,^{+0.12}_{-0.06}(\mathrm{theo.})\right]$
$|\kappa _{{ b}}|$	$=$ $0.62 \pm 0.28$	$0.64^{+0.34}_{-0.28}$	$0.62^{+0.31}_{-0.27}$	$\left[ ^{+0.21}_{-0.20}(\mathrm{stat.})\,^{+0.17}_{-0.18}(\mathrm{syst.})\,^{+0.06}_{-0.03}(\mathrm{theo.})\right]$
$|\kappa _\tau |$	$=$ $0.99^{+0.22}_{-0.18}$	$1.03^{+0.21}_{-0.40}$	$1.00\pm 0.20$	$\left[ ^{+0.15}_{-0.14}(\mathrm{stat.})\,^{+0.12}_{-0.11}(\mathrm{syst.})\,^{+0.06}_{-0.04}(\mathrm{theo.})\right]$
$|\kappa _\mu |$	$<$ $2.3$ (95 % CL)	2.8 (95 % CL)	2.3 (95 % CL)	
$\kappa _{{\gamma }}$	$=$ $0.90^{+0.16}_{-0.14}$	$=0.93\pm ^{0.36}_{0.17}$	$0.90 \pm 0.15$	$\left[ ^{+0.13}_{-0.12}(\mathrm{stat.})\,^{+0.07}_{-0.07}(\mathrm{syst.})\,^{+0.04}_{-0.03}(\mathrm{theo.})\right]$
$\kappa _{{ g}}$	$=$ $0.92^{+0.23}_{-0.16}$	$1.02\pm ^{0.37}_{0.19}$	$0.92 \pm 0.17$	$\left[ ^{+0.14}_{-0.12}(\mathrm{stat.})\,^{+0.10}_{-0.09}(\mathrm{syst.})\,^{+0.07}_{-0.05}(\mathrm{theo.})\right]$
$\kappa _{{ Z}{\gamma }}$	$<$ $3.15$ (95 % CL)	4.03 (95 % CL)	3.18 (95 % CL)	
$\mathrm {BR_{i.,u.}}$	$<$ $0.49$ (95 % CL)	0.68 (95 % CL)	–	
$\Gamma _H/\Gamma _H^\mathrm{SM}$	$=$ $0.64^{+0.40}_{-0.25}$	$0.74^{+1.57}_{-0.21}$	$0.64 ^{+0.31}_{-0.25}$	$\left[ ^{+0.24}_{-0.21}(\mathrm{stat.})\,^{+0.19}_{-0.15}(\mathrm{syst.})\,^{+0.06}_{-0.05}(\mathrm{theo.})\right]$

**Fig. 24:**
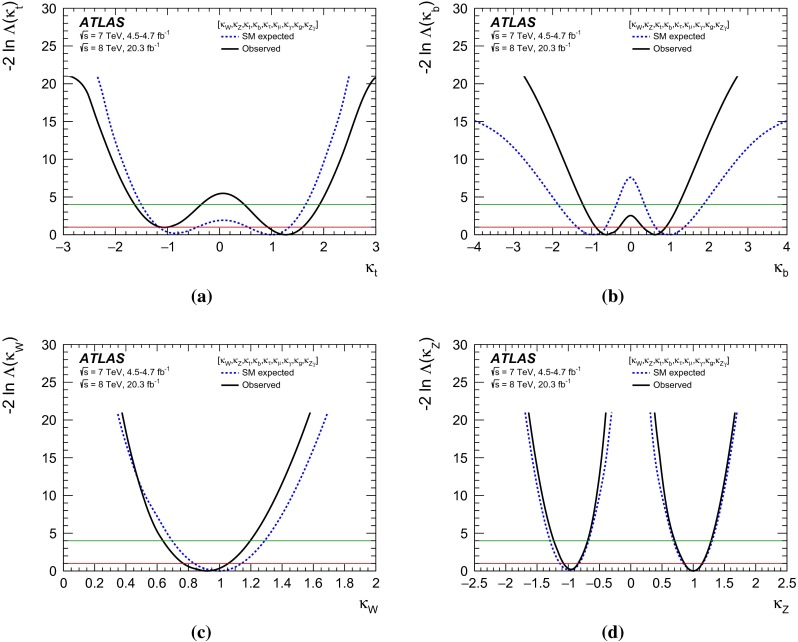
Results of fits for generic model 2 (see text): profile likelihood ratios as functions of the coupling-strength scale factors **a** $\kappa _{{ t}}$, **b** $\kappa _{{ b}}$, **c** $\kappa _{{ W}}$, and **d** $\kappa _{{ Z}}$. For each measurement, the other coupling-strength scale factors are profiled. The *red* (*green*) *horizontal line* indicates the value of the profile likelihood ratio corresponding to a 68 % (95 %) confidence interval for the parameter of interest, assuming the asymptotic $\chi ^2$ distribution for the test statistic

Figure 22 shows the results of the fit for generic model 1 as reduced coupling-strength scale factors

10 $$\begin{aligned} y_{V,i} = \sqrt{ \kappa _{V,i} \frac{g_{V,i}}{2v} } = \sqrt{\kappa _{V,i}}\frac{m_{V,i}}{v} \end{aligned}$$

for weak bosons with a mass $m_{V}$, where $g_{V,i}$ is the absolute Higgs boson coupling strength, *v* is the vacuum expectation value of the Higgs field and

11 $$\begin{aligned} y_{F,i} = \kappa _{F,i}\frac{g_{F,i}}{\sqrt{2}} = \kappa _{F,i}\frac{m_{F,i}}{v} \end{aligned}$$

for fermions as a function of the particle mass $m_{F}$, assuming a SM Higgs boson with a mass of 125.36 GeV. For the *b*-quark mass in Fig. 22 the $\overline{MS}$ running mass evaluated at a scale of 125.36 GeV is assumed.

#### Generic model 2: allow new particles in loops and in decay

In the second generic benchmark model the six free parameters from the first generic model are retained but the assumptions on the absence of BSM contributions in loops and to the total width are dropped. Effective coupling-strength scale factors for loop vertices are introduced, and optionally a branching ratio $\mathrm {BR_{i.,u.}}$ to new non-SM decays that might yield invisible or undetected final states is introduced, resulting in a total of 9 (10) free parameters. In the variant where $\mathrm {BR_{i.,u.}}$ is not fixed to zero, either the constraint $\kappa _{V}<1$ is imposed, or the constraint on the total width from off-shell measurements is included.

Figure 23 summarises the results of the fits for this benchmark scenario. The numerical results are shown in Table 11. As an illustration of contributions from different sources, the uncertainty components are shown for the case of $\mathrm {BR_{i.,u.}} =0$. All fundamental coupling-strength scale factors, as well as the loop-coupling scale factors $\kappa _{{ g}}$ and $\kappa _{{\gamma }}$ are measured to be compatible with their SM expectation under all explored assumptions, while limits are set on the loop-coupling scale factor $\kappa _{{ Z}{\gamma }}$ and the fraction of Higgs boson decays to invisible or undetected decays. When imposing the physical constraint $\mathrm {BR_{i.,u.}} \ge 0$ in the inference on $\mathrm {BR_{i.,u.}}$, the $95\%$ CL upper limit is $\mathrm {BR_{i.,u.}} < 0.49$ ($0.68$) under the constraint $\kappa _{V}<1$ ($\kappa _\mathrm{on}=\kappa _\mathrm{off}$) on the Higgs boson total width. The nine-dimensional compatibility of the SM hypothesis with the best-fit point is $73~\%$ when $\mathrm {BR_{i.,u.}}$ is fixed to zero. The compatibilities for the fits with the conditions $\kappa _{V}<1$ and $\kappa _\mathrm{on}=\kappa _\mathrm{off}$ imposed are 80 and $57~\%$, respectively.

Similar to the results of the benchmark model in Sect. 5.2.2 the upper bound of the 68 % CL interval for the scenario $\kappa _\mathrm{on}=\kappa _\mathrm{off}$ should be considered to be only approximate due to deviations of the test-statistic distribution from its asymptotic form. The deviation of the asymptotic distribution was shown to be negligible for off-shell signal strengths corresponding to the upper end of the 95 % asymptotic confidence interval (Table 11).

Also shown in Fig 23 are the resulting ranges of the total width of the Higgs boson, expressed as the ratio $\Gamma _{{ H}}/\Gamma _{{ H}}^\mathrm{SM}$. These estimates are obtained from alternative parameterisations of these benchmark models, where the effective coupling-strength scale factor $\kappa _{g}$ is replaced by the expression that results from solving Eq. (8) for $\kappa _{g}$, introducing $\Gamma _{{ H}}/\Gamma _{{ H}}^\mathrm{SM}$ as a parameter of the model. The figure shows that the upper bound on the Higgs boson width from the assumption $\kappa _\mathrm{on}=\kappa _\mathrm{off}$ is substantially weaker than the bound from the assumption $\kappa _{V}<1$. These results on $\Gamma _{{ H}}/\Gamma _{{ H}}^\mathrm{SM}$ represent the most model-independent measurements of the Higgs boson total width presented in this paper.

Figure 24 shows profile likelihood ratios as a function of selected coupling-strength scale factors. In Fig. 24a, the negative minimum of $\kappa _{{ t}}$ is shown to be disfavoured at $1.0\sigma$. The minimum corresponding to the positive solution is found at $\kappa _{{ t}} = 1.28^{+0.32}_{-0.35}$. The sensitivity to disfavour the negative solution of $\kappa _{{ t}}$ is reduced with respect to generic model 1 as the interference in loop couplings can no longer be exploited because effective coupling-strength scale factors were introduced. The observed residual sensitivity to the sign of $\kappa _{{ t}}$ is exclusively due to the tree-level interference effect of the *tH* background in the *ttH* channel.

**Fig. 25:**
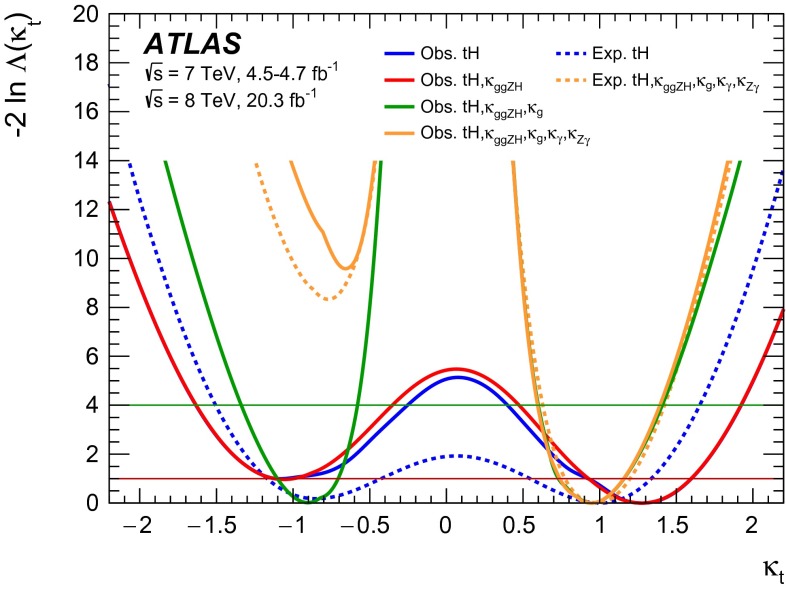
Profile likelihood ratio as a function of $\kappa _{{ t}}$ for models with and without resolved loop processes: shown are measurements of $\kappa _{{ t}}$ with no loop processes resolved (*blue*), only $gg\rightarrow ZH$ resolved (*red*, generic model 2), $gg\rightarrow H$ additionally resolved (*green*), and $H{\rightarrow \,}\gamma \gamma$ and $H \rightarrow Z \gamma$ additionally resolved (*orange*, generic model 1). The *dashed blue* and *orange curves* correspond to the expected sensitivity for the no-loop and all-loop models. All profile likelihood curves are drawn for the full range of $\kappa _{{ t}}$, however some curves are partially obscured when overlapping with another nearly identical curve. The *red* (*green*) *horizontal line* indicates the value of the profile likelihood ratio corresponding to a 68 % (95 %) confidence interval for the parameter of interest, assuming the asymptotic $\chi ^2$ distribution for the test statistic

The power of individual loop processes to measure the magnitude of $\kappa _{{ t}}$ and resolve the sign of $\kappa _{{ t}}$ relative to $\kappa _{{ W}}$ is illustrated in more detail in Fig. 25. The blue curve shows the profile likelihood ratio as a function of $\kappa _{{ t}}$ for a model with the least sensitivity to the sign of $\kappa _{{ t}}$: all loop processes are described with effective coupling parameters, including the $gg\rightarrow ZH$ loop process. Subsequently the red, green and orange curves represent the profile likelihood ratios for models that incrementally include information from loop processes by resolving the $gg\rightarrow ZH$, ggF and $H\rightarrow \gamma \gamma ,\,Z\gamma$ loop processes into their expected SM content. Here the red curve corresponds to the configuration of generic model 2, and the orange curve corresponds to the configuration of generic model 1. As expected, resolving $gg\rightarrow ZH$ process adds little information on $\kappa _{{ t}}$. Additionally resolving the ggF loop process into its SM content greatly improves the precision on $\kappa _{{ t}}$ (green curve), but reduces the sensitivity to the relative sign of $\kappa _{{ t}}$ and $\kappa _{{ W}}$. This reduction happens because on one hand the ggF process yields no new information on this relative sign, as it is dominated by *t*–*b* interference, and on the other hand because it decreases the observed magnitude of $\kappa _{{ t}}$ to a more SM-compatible level, thereby reducing the sensitivity of the *tH* process to the relative sign. Further resolving the $H{\rightarrow \,}\gamma \gamma$ and $H \rightarrow Z \gamma$ loop processes, which are dominated by *W*–*t* interference, greatly improves the measurement of the relative sign of $\kappa _{{ W}}$ and $\kappa _{{ t}}$ (orange curve), but does not significantly contribute to the precision of the magnitude of $\kappa _{{ t}}$.

**Table 12:** Numerical results of the fits for generic model 3: measurements of ratios of coupling-strength scale factors in which assumptions on the Higgs boson total width cancel. These results are also shown in Fig. 26. Shown in square brackets are uncertainty components from different sources. For $\lambda _{{ W}{ Z}}$ and $\lambda _{{ t}{ g}}$, the uncertainty breakdowns are provided for the preferred positive solutions

Parameter		Measurement	Uncertainty breakdown
$\kappa _{{ g}{ Z}}$	$=$	$1.18 \pm 0.16$	$\left[ ^{+0.14}_{-0.14}(\mathrm{stat.})\,^{+0.04}_{-0.04}(\mathrm{syst.})\,^{+0.08}_{-0.06}(\mathrm{theo.})\right]$
$\lambda _{{ Z}{ g}}$	$=$	$1.09^{+0.26}_{-0.22}$	$\left[ ^{+0.21}_{-0.20}(\mathrm{stat.})\,^{+0.12}_{-0.10}(\mathrm{syst.})\,^{+0.08}_{-0.06}(\mathrm{theo.})\right]$
$\lambda _{{ W}{ Z}}$	$\in$	$[-1.04,-0.81] \cup [0.80,1.06]$	$\left[ ^{+0.13}_{-0.11}(\mathrm{stat.})\,^{+0.05}_{-0.05}(\mathrm{syst.})\,^{+0.02}_{-0.02}(\mathrm{theo.})\right]$
$\kappa _{{ g}{ Z}}$	$=$	$1.18 \pm 0.16$	$\left[ ^{+0.14}_{-0.14}(\mathrm{stat.})\,^{+0.04}_{-0.04}(\mathrm{syst.})\,^{+0.08}_{-0.06}(\mathrm{theo.})\right]$
$\lambda _{{ Z}{ g}}$	$=$	$1.09^{+0.26}_{-0.22}$	$\left[ ^{+0.21}_{-0.20}(\mathrm{stat.})\,^{+0.12}_{-0.10}(\mathrm{syst.})\,^{+0.08}_{-0.06}(\mathrm{theo.})\right]$
$\lambda _{{ W}{ Z}}$	$\in$	$[-1.04,-0.81] \cup [0.80,1.06]$	$\left[ ^{+0.13}_{-0.11}(\mathrm{stat.})\,^{+0.05}_{-0.05}(\mathrm{syst.})\,^{+0.02}_{-0.02}(\mathrm{theo.})\right]$
$\lambda _{{ t}{ g}}$	$\in$	$[-1.70, -1.07] \cup [1.03,1.73]$	$\left[ ^{+0.26}_{-0.25}(\mathrm{stat.})\,^{+0.20}_{-0.24}(\mathrm{syst.})\,^{+0.14}_{-0.08}(\mathrm{theo.})\right]$
$\lambda _{{ b}{ Z}}$	$=$	$0.60 \pm 0.27$	$\left[ ^{+0.21}_{-0.19}(\mathrm{stat.})\,^{+0.14}_{-0.16}(\mathrm{syst.})\,^{+0.05}_{-0.03}(\mathrm{theo.})\right]$
$\lambda _{{\tau }{ Z}}$	$=$	$0.99^{+0.23}_{-0.19}$	$\left[ ^{+0.19}_{-0.16}(\mathrm{stat.})\,^{+0.11}_{-0.09}(\mathrm{syst.})\,^{+0.06}_{-0.04}(\mathrm{theo.})\right]$
$|\lambda _{{\mu }{ Z}}|$	$<$	2.3 (95 % CL)	
$\lambda _{{\gamma }{ Z}}$	$=$	$0.90 \pm 0.15$	$\left[ ^{+0.15}_{-0.13}(\mathrm{stat.})\,^{+0.05}_{-0.04}(\mathrm{syst.})\,^{+0.03}_{-0.03}(\mathrm{theo.})\right]$
$|\lambda _{({ Z}{\gamma }){ Z}}|$	$<$	3.2 (95 % CL)	

#### Generic model 3: allow new particles in loops, no assumptions on the total width

In the final benchmark model of this section, the six absolute coupling-strength scale factors and three effective loop-coupling scale factors of generic model 2 are expressed as ratios of scale factors that can be measured independent of any assumptions on the Higgs boson total width. The free parameters are chosen as:

$$\begin{aligned} \kappa _{{ g}{ Z}}= & {} \kappa _{{ g}}\cdot \kappa _{{ Z}} / \kappa _{{ H}} \\ \lambda _{{ Z}{ g}}= & {} \kappa _{{ Z}} / \kappa _{{ g}} \\ \lambda _{{ W}{ Z}}= & {} \kappa _{{ W}} / \kappa _{{ Z}} \\ \lambda _{{ t}{ g}}= & {} \kappa _{{ t}} / \kappa _{{ g}} \\ \lambda _{{ b}{ Z}}= & {} \kappa _{{ b}} / \kappa _{{ Z}} \\ \lambda _{{\tau }{ Z}}= & {} \kappa _{\tau } / \kappa _{{ Z}} \\ \lambda _{{\mu }{ Z}}= & {} \kappa _{\mu } / \kappa _{{ Z}} \\ \lambda _{{\gamma }{ Z}}= & {} \kappa _{{\gamma }} / \kappa _{{ Z}} \\ \lambda _{({ Z}{\gamma }){ Z}}= & {} \kappa _{{ Z}{\gamma }} / \kappa _{{ Z}} . \end{aligned}$$

**Fig. 26:**
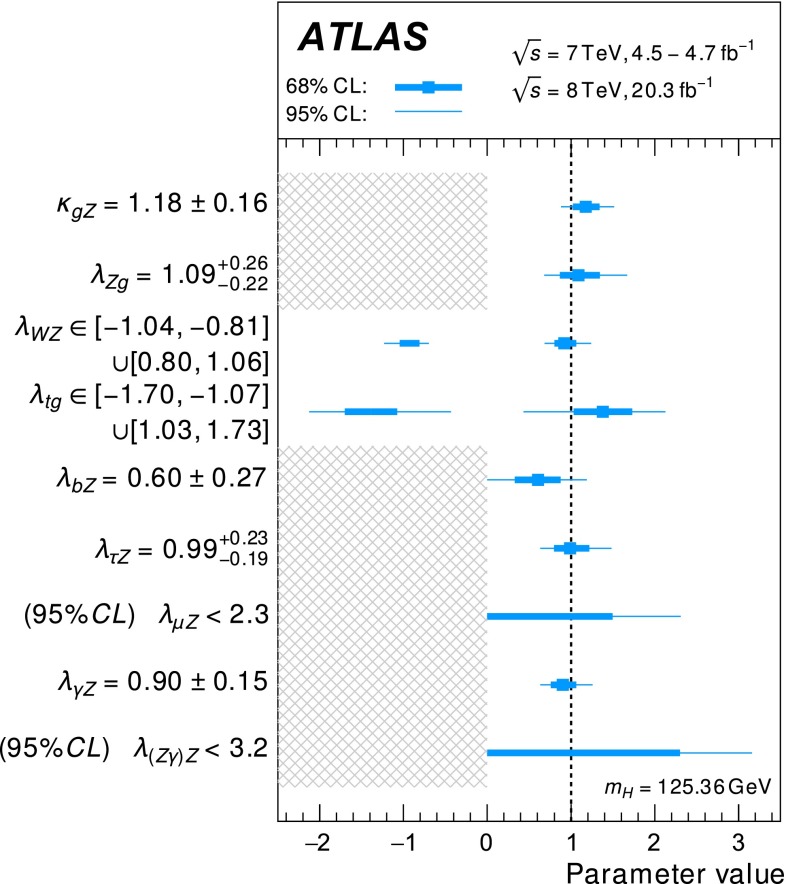
Results of fits for generic model 3 (see text): allowing deviations in vertex loop-coupling scale factors and in the total width. Overview of best-fit values of parameters, where the *inner* and *outer bars* correspond to 68 and 95 % CL intervals. The *hatched areas* indicate regions that are outside the defined parameter boundaries

Figure 26 shows the full set of results obtained from the fit to this benchmark model. The fitted values and their uncertainties are also shown in Table 12. As the loop-induced processes are expressed by effective coupling-strength scale factors, there is little sensitivity to the relative sign of coupling-strength scale factors due to *tH* and $gg\rightarrow ZH$ processes only. Hence only positive values for all $\kappa$-factors except $\kappa _{{ t}}$ are shown without loss of generality. The parameter $\kappa _{{ g}{ Z}},\lambda _{{ Z}{ g}},\lambda _{{ W}{ Z}},\lambda _{{ t}{ g}},\lambda _{{ b}{ Z}},\lambda _{{\tau }{ Z}}$ and $\lambda _{{\gamma }{ Z}}$ are all measured to be compatible with their SM expectation, while limits are set on the parameters $\lambda _{{\mu }{ Z}}$ and $\lambda _{({ Z}{\gamma }){ Z}}$. The nine-dimensional compatibility of the SM hypothesis with the best-fit point is $73~\%$.

**Fig. 27:**
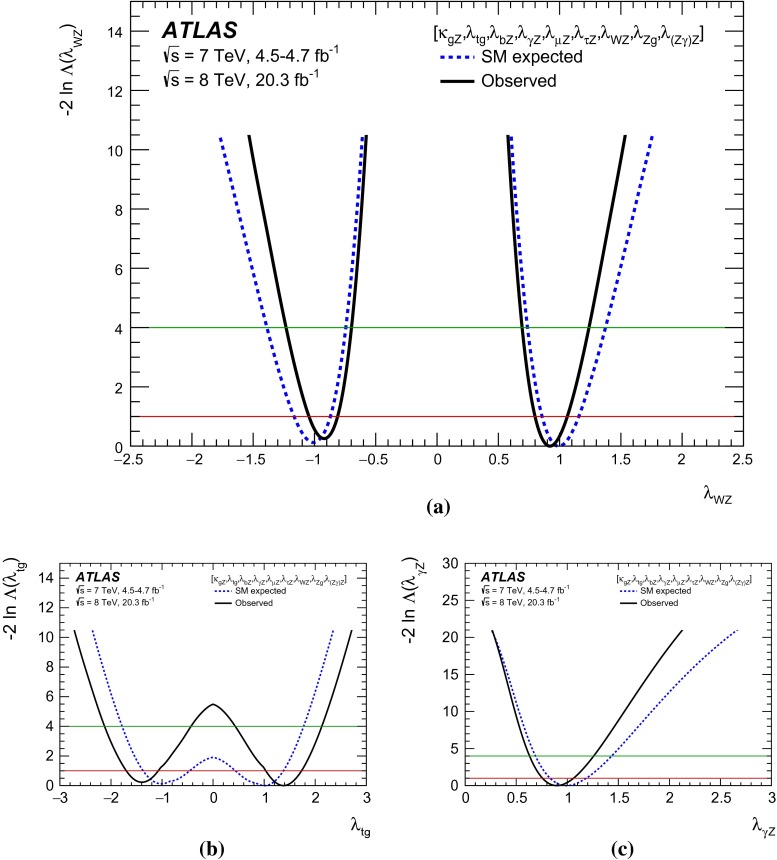
Results of fits for generic model 3 (see text): profile likelihood ratios as functions of the coupling-strength scale factor ratios **a** $\lambda _{{ W}{ Z}}$, **b** $\lambda _{{ t}{ g}}$ and **c** $\lambda _{{\gamma }{ Z}}$. In all cases, the other parameters are profiled. The *dashed curves* show the SM expectations. The *red* (*green*) *horizontal line* indicates the cutoff value of the profile likelihood ratio corresponding to a 68 % (95 %) confidence interval for the parameter of interest, assuming the asymptotic $\chi ^2$ distribution for the test statistic

The parameter $\lambda _{{ W}{ Z}} =\kappa _{{ W}}/\kappa _{{ Z}}$ in this model is of particular interest: identical coupling-strength scale factors for the *W* and *Z* bosons are required within tight bounds by the $\mathrm {SU(2)}$ custodial symmetry and the $\rho$ parameter measurements at LEP and at the Tevatron [120]. This custodial constraint is directly probed in the Higgs sector through the parameter $\lambda _{{ W}{ Z}}$. The measured ratio $\lambda _{{ W}{ Z}}$ is in part directly constrained by the decays in the $H{\rightarrow \,}WW^{*}{\rightarrow \,}\ell \nu \ell \nu$ and $H{\rightarrow \,}ZZ^{*}{\rightarrow \,}4\ell$ channels and the *WH* and *ZH* production processes. It is also indirectly constrained by the VBF production process, which in the SM is $74~\%$*W* fusion-mediated and $26~\%$*Z* fusion-mediated (see Table 9). Figure 27a shows the profile likelihood ratio as a function of the coupling-strength scale factor ratio $\lambda _{{ W}{ Z}}$. Due to the interference terms, the fit is sensitive to the relative sign of the *W* and *t* coupling (*tH*) and the relative sign of the *Z* and *t* coupling ($gg\rightarrow ZH$), providing indirect sensitivity to the sign of $\lambda _{{ W}{ Z}}$. The negative solution is disfavoured at $0.5\sigma$ ($0.3\sigma$ expected). The minimum corresponding to the positive solution is found at $\lambda _{{ W}{ Z}} =0.92^{+0.14}_{-0.12}$, in excellent agreement with the prediction of $\mathrm {SU(2)}$ custodial symmetry.

Also shown in Fig. 27b, c are the ratios $\lambda _{{\gamma }{ Z}}$ and $\lambda _{{ t}{ g}}$. The ratio $\lambda _{{\gamma }{ Z}}$ is sensitive to new charged particles contributing to the $H{\rightarrow \,}\gamma \gamma$ loop in comparison to $H \rightarrow ZZ^{*}$ decays. Similarly, the ratio $\lambda _{{ t}{ g}}$ is sensitive to new coloured particles contributing through the $gg\rightarrow H$ loop as compared to *ttH*. The minimum corresponding to the positive solution is found at $\lambda _{{ t}{ g}} = 1.38 \pm 0.35$. Both are observed to be compatible with the SM expectation.

The fit in the third generic benchmark model uses only the basic assumptions, as stated at the beginning of this section, and hence represents the most model-independent determination of coupling-strength scale factors that is currently possible.

## Conclusion

The Higgs boson production and decay properties are studied using proton–proton collision data collected by the ATLAS experiment at the Large Hadron Collider corresponding to integrated luminosities of up to 4.7 $\mathrm{fb}^{-1}$ at $\sqrt{s}=7$ TeV and 20.3 $\mathrm{fb}^{-1}$ at $\sqrt{s}=8$ TeV. The study combines specific analyses of the $H\rightarrow \gamma \gamma ,\,ZZ^*,\,WW^*,\,Z\gamma ,\,b\bar{b},\,\tau \tau \,$ and $\mu \mu$ decay channels, as well as searches for *ttH* production and measurements of off-shell Higgs boson production. It significantly extends a previous combination of the $H\rightarrow \gamma \gamma ,\,ZZ^*$ and $WW^*$ decays [23]. In particular, the addition of the fermionic decays of the Higgs boson in the combinations allows for direct tests of the Yukawa interactions of the Higgs boson with fermions.

The measured Higgs boson signal yields are compared with the SM expectations at the fixed Higgs boson mass of $m_H=125.36$ GeV. The combined yield relative to its SM prediction is determined to be $1.18 \pm 0.10\,(\mathrm{stat.})\pm 0.07\,(\mathrm{syst.})\,^{+0.08}_{-0.07}\,(\mathrm{theo.})$. The combined analysis provides unequivocal confirmation of gluon fusion production of the Higgs boson with a significance exceeding $5\sigma$ and strong evidence of vector-boson fusion production with a significance of $4.3\sigma$. Furthermore, it supports the SM predictions of Higgs boson production in association with a vector boson or a pair of top quarks. Values for the total cross sections can be obtained from the signal strength of each production process within the uncertainties related to the modelling of Higgs boson production and decay kinematics and assuming SM decay branching ratios. The total cross sections at $\sqrt{s}=7$ and 8 TeV are $22.1\,^{+6.7}_{-5.3}\,(\mathrm{stat.})\,^{+2.7}_{-2.3}\,(\mathrm{syst.})\,^{+1.9}_{-1.4}\,(\mathrm{theo.})$ pb and $27.7\pm 3.0\, (\mathrm{stat.})\,^{+2.0}_{-1.7}\,(\mathrm{syst.})\,^{+1.2}_{-0.9}\,(\mathrm{theo.})$ pb, respectively.

The observed Higgs boson production and decay rates are also interpreted in a leading-order coupling framework, exploring a wide range of benchmark coupling models both with and without assumptions about the Higgs boson width and the SM particle content of loop processes. Higgs boson couplings to up-type fermions and vector bosons are found with both significances above $5\sigma$ and to down-type fermions with a significance of $4.5\sigma$, under the assumption of unified coupling scale factors, one for each type of particles. In a different model with separate unified coupling scale factors for leptons, quarks and vector bosons, Higgs boson couplings to leptons are found with a significance of $\sim \! 4.4\sigma$.

The Higgs boson coupling strengths to fermions and bosons are measured with a precision of $\pm$16 and $\pm$7 % respectively, when assuming the SM Higgs boson width, and are observed to be compatible with the SM expectations. Coupling strengths of loop processes are measured with a precision of $\pm$12 % when assuming the SM expectations for non-loop Higgs boson coupling strengths and the Higgs boson total width, increasing to about $\pm$20 % when these assumptions are removed. No significant deviations from the SM expectations of Higgs boson coupling strengths in loop processes are observed.

Measurements of coupling strengths to $\mu ,\,\tau$ leptons, $b,\,t$ quarks and $W,\,Z$ bosons, or ratios of these coupling strengths are presented in the context of generic Higgs boson coupling models. They can constrain the ratio of *W* and *Z* coupling strengths, a probe of custodial symmetry, with a precision of $\pm$13 %. For benchmark models that measure absolute coupling strengths, a variety of physics-motivated constraints on the Higgs boson total width have been explored. The measured Higgs boson coupling strengths and their precision are found to depend only weakly on the choice of these constraints. A third generic benchmark model uses only the most basic assumptions and hence represents the most model-independent determination of the coupling strength scale factors that is currently possible. In this model ratios of couplings are constrained with a precision of 15–40 %.

The *p*-values expressing compatibility of the SM hypothesis with the best-fit point range between 29 and 99 % for all considered benchmark models. The observed data are thus very compatible with the SM expectation under a wide range of assumptions.

**Table 13:** Best-fit values of $gg\rightarrow H\rightarrow WW^*$ signal strength $\mu _\mathrm{ggF}^{WW^*}$, ratios of cross sections $R_{i/\mathrm{ggF}}$ and of branching ratios $\rho _{f/WW^*}$. All $R_{i/\mathrm{ggF}}$ and $\rho _{f/WW^*}$ are measured relative to their SM values for $m_H=125.36$ GeV from the combined analysis of the $\sqrt{s}=7$ and 8 TeV data. Shown in square brackets are uncertainty components: statistical (first), systematic (second) and signal theoretical (third) uncertainties

Parameter	Best-fit value
$\mu _\mathrm{ggF}^{WW^*}$	$1.15\,^{+0.28}_{-0.24}$ $\left[ {^{+0.18}_{-0.18}}\, {^{+0.12}_{-0.11}}\, {^{+0.17}_{-0.12}}\right]$
$R_{\mathrm{VBF}/\mathrm{ggF}}$	$0.99\,^{+0.46}_{-0.33}$ $\left[ {^{+0.37}_{-0.29}}\, {^{+0.20}_{-0.12}}\, {^{+0.18}_{-0.10}}\right]$
$R_{WH/\mathrm{ggF}}$	$1.47\,^{+1.06}_{-0.74}$ $\left[ {^{+0.87}_{-0.65}}\, {^{+0.49}_{-0.32}}\, {^{+0.34}_{-0.15}}\right]$
$R_{ZH/\mathrm{ggF}}$	$0.60\,^{+1.39}_{-0.66}$ $\left[ {^{+0.99}_{-0.60}}\, {^{+0.93}_{-0.25}}\, {^{+0.30}_{-0.07}}\right]$
$R_{ttH/\mathrm{ggF}}$	$1.81\,^{+1.10}_{-0.81}$ $\left[ {^{+0.79}_{-0.64}}\, {^{+0.61}_{-0.48}}\, {^{+0.46}_{-0.17}}\right]$
$\rho _{\gamma \gamma /WW^*}$	$0.97\,^{+0.32}_{-0.25}$ $\left[ {^{+0.26}_{-0.22}}\, {^{+0.15}_{-0.10}}\, {^{+0.10}_{-0.06}}\right]$
$\rho _{ZZ^*/WW^*}$	$1.24\,^{+0.42}_{-0.31}$ $\left[ {^{+0.37}_{-0.29}}\, {^{+0.18}_{-0.10}}\, {^{+0.07}_{-0.04}}\right]$
$\rho _{\tau \tau /WW^*}$	$1.20\,^{+0.52}_{-0.38}$ $\left[ {^{+0.40}_{-0.32}}\, {^{+0.29}_{-0.18}}\, {^{+0.17}_{-0.09}}\right]$
$\rho _{bb/WW^*}$	$0.59\,^{+0.63}_{-0.37}$ $\left[ {^{+0.45}_{-0.27}}\, {^{+0.43}_{-0.24}}\, {^{+0.12}_{-0.05}}\right]$

## Appendix A: Alternative parameterisation of ratios of cross sections and of branching ratios

An alternative to the parameterisation of Sect. 4.4 is to normalise the ratios of cross sections and of branching ratios to their SM values. Compared with Eq. (6), the yield of the production and decay $i\rightarrow H\rightarrow f$ can be parameterised as

12 $$\begin{aligned} \sigma _i\cdot \mathrm{BR}_f= & {} \mu _i^f \times \left[ \sigma _i\cdot \mathrm{BR}_f\right] _\mathrm{SM}\nonumber \\ {}= & {} \left( \mu _\mathrm{ggF}^{WW^*}\cdot R_{i/\mathrm{ggF}}\cdot \rho _{f/WW^*}\right) \times \left[ \sigma _i\cdot \mathrm{BR}_f\right] _\mathrm{SM}\,.\nonumber \\ \end{aligned}$$

Here *R* and $\rho$ are ratios of cross sections and branching ratios relative to their SM expectations, respectively:

13 $$\begin{aligned}&R_{i/\mathrm{ggF}} = \frac{\sigma _i/\sigma _\mathrm{ggF}}{\left[ \sigma _i/\sigma _\mathrm{ggF}\right] _\mathrm{SM}} ~\mathrm{and}\nonumber \\&\quad \rho _{f/WW^*} = \frac{\mathrm{BR}_f/\mathrm{BR}_{WW^*}}{\left[ \mathrm{BR}_f/\mathrm{BR}_{WW^*}\right] _\mathrm{SM}}. \end{aligned}$$

The data are fitted with $\mu _\mathrm{ggF}^{WW^*}$, four ratios of production cross sections and one ratio of branching ratios for each decay channel other than the $H\rightarrow WW^*$ decay. The results shown in Table 13 are nearly identical to the best-fit values relative to their SM predictions shown in Table 7. The small differences are expected from the inclusion of additional nuisance parameters of the SM predictions and from the precision of the fits. One clear advantage of the parameterisation of Sect. 4.4 is that the results are independent of the SM predictions and are, therefore, not affected by the theoretical uncertainties of the predictions. Consequently, the fitted values of the ratios of cross sections and of partial decay widths shown in Table 7 have significantly smaller theoretical uncertainties than their counterparts ($R_{i/\mathrm{ggF}}$ and $\rho _{r/WW^*}$) in Table 13. The former is only affected by the theoretical uncertainties in the modelling of Higgs boson production whereas the latter suffer from both the modelling uncertainties and the uncertainties of the SM predictions.

## Footnotes

1 For events with two leptons, a requirement on the invariant mass of the $\tau \tau$ system reconstructed via the collinear approximation also ensures orthogonality with the $H\rightarrow WW^*\rightarrow \ell \nu \ell \nu$ analysis.

2 Whenever probabilities are translated into the number of Gaussian standard deviations the two-sided convention is chosen.

3 The *p*-value is defined as the probability to obtain a value of the test statistic that is at least as high as the observed value, under the hypothesis that is being tested.

4 The measurement of the $q\bar{q}/gg \rightarrow t\bar{t}H$ signal strength in the multiple-lepton decay mode contributes to all final states with leptons in Fig. 2, according to the prediction of MC simulation, i.e. predominantly to the $H\rightarrow WW^*$ and $H\rightarrow ZZ^*$ final states.

5 Invisible final states can be directly searched for through the $E_{\text {T}}^{\text {miss}}$ signature [28]. An example of an undetected mode would be a decay mode to multiple light jets, which presently cannot be distinguished from multijet backgrounds.

6 The *ZH* production cross section quoted in Table 1 comprises both the $q\bar{q}\rightarrow ZH$ and $gg\rightarrow ZH$ processes.
